# Meta-analysis of QTL reveals the genetic control of yield-related traits and seed protein content in pea

**DOI:** 10.1038/s41598-020-72548-9

**Published:** 2020-09-28

**Authors:** Anthony Klein, Hervé Houtin, Céline Rond-Coissieux, Myriam Naudet-Huart, Michael Touratier, Pascal Marget, Judith Burstin

**Affiliations:** 1 Agroécologie, INRAE, AgroSup Dijon, Univ. Bourgogne, Univ. Bourgogne Franche-Comté, 21000 Dijon, France; 2 INRAE, U2E, Unité Expérimentale du Domaine d’Epoisses, Centre de Recherches Bourgogne Franche-Comté, 21110 Breteniere, France

**Keywords:** Plant breeding, Plant genetics

## Abstract

Pea is one of the most important grain legume crops in temperate regions worldwide. Improving pea yield is a critical breeding target. Nine inter-connected pea recombinant inbred line populations were evaluated in nine environments at INRAE Dijon, France and genotyped using the GenoPea 13.2 K SNP array. Each population has been evaluated in two to four environments. A multi-population Quantitative Trait Loci (QTL) analysis for seed weight per plant (SW), seed number per plant (SN), thousand seed weight (TSW) and seed protein content (SPC) was done. QTL were then projected on the multi-population consensus map and a meta-analysis of QTL was performed. This analysis identified 17 QTL for SW, 16 QTL for SN, 35 QTL for TSW and 21 QTL for SPC, shedding light on trait relationships. These QTL were resolved into 27 metaQTL. Some of them showed small confidence intervals of less than 2 cM encompassing less than one hundred underlying candidate genes. The precision of metaQTL and the potential candidate genes reported in this study enable their use for marker-assisted selection and provide a foundation towards map-based identification of causal polymorphisms.

## Introduction

Grain legumes play a central role in sustainable agriculture and food security. They produce protein-rich seeds and thanks to their special nitrogen nutrition largely based on a symbiosis with nitrogen-fixing soil bacteria, they allow for the reduction of nitrogen fertilizer use in cropping systems thus lowering agriculture energy costs and greenhouse gas production^1,2^. Pea (*Pisum sativum* L.) is one of the most cultivated grain legumes in temperate areas^3^. It has a high nutritional value and is used for human food and animal feed. With a demand for plant proteins rising worldwide, increasing pea seed yield and protein content are important breeding targets.

Seed yield and seed protein content are complex traits and highly quantitative. Numerous loci controlling seed yield and seed quality have been identified in pea^4–14^. The r and rb loci encoding Starch-Branching Enzyme 1^15^ and ADP glucose-pyrophosphorylase^16^, respectively, and which control the wrinkled seed phenotype in pea have long been known to impact seed development, yield and seed protein content^17,18^. Burstin et al.^7^ suggested that seed yield and protein content QTL corresponded either to (i) genes controlling the plant source capacity to produce and fill seeds, or (ii) genes controlling seed sink strength such as genes involved in seed formation and storage products’ synthesis. Genes controlling plant architecture such as *Le* which determines inter-node length or *Afila* (*Af)* which determines leaf type would more likely correspond to source capacity loci while *rugosus* genes or a gene encoding a subtilase shown to be associated with a seed size QTL^19^ would correspond to sink strength loci. Bourgeois et al.^8^ have further shown that most protein quality QTL were co-localized with genes encoding major storage proteins.

Identifying causal polymorphisms for these traits has been challenging in pea. Most QTL-based research studies have considered a few bi-parental populations. As a result, the QTL detection was limited to phenotypic variability of both parents and the QTL confidence intervals were generally large, probably due to the limited population size or low-density linkage maps. To narrow QTL confidence intervals and provide a foundation to marker-assisted breeding for yield components and seed protein content in pea, we investigated these traits in nine inter-connected recombinant inbred line populations (RIL) derived from parents showing contrasted phenotypes^20^ providing a wide phenotypic variability. We used a high-density single nucleotide polymorphism (SNP) based genotyping platform, namely GenoPea 13.2 K SNP array^20^, to ensure high-quality dense genetic maps. We performed QTL mapping taking advantage of the multi-cross design^21^ to define QTL locations and genotypic effects. We then integrated all QTL results through a meta-analysis approach^22^. The wide phenotypic variability and the high-density linkage map allowed the identification of metaQTL for seed weight per plant (SW), seed number per plant (SN), thousand seed weight (TSW) and seed protein content (SPC). This refined confidence intervals and pinpointed some candidate genes thanks to the recently published pea genome sequence^23^.

## Results

### Phenotypic variation for SW, SN, TSW and SPC in multiple populations

SW, SN, TSW and SPC measured in the field and the glasshouse for 1213 RIL from nine bi-parental populations revealed highly-significant line, year, and population effects (Supplementary Table S1). The traits exhibited continuous distributions in the nine populations indicating a polygenic inheritance (Supplementary Fig. S1). Transgressive lines were observed for all traits but TSW in Pop11, indicating that favourable alleles are brought by both parents in most cases. Highly-significant genotype effects (*P* < 0.0001) and high heritabilities were detected for the four traits in almost all populations and environments, except for Pop3, Pop4 and Pop5 in the field experiment in 2004 due to the impact of diseases on plants and Pop11 in the glasshouse in 2008 related to the F_3_ state of the population (Supplementary Table S1). The range of the data of the multi-population lines phenotyped in field environment was extensive: in spring sowing, SW varied from 1.7 to 47.9 g per plant; SN from 9.9 to 300.0, TSW from 78.6 to 317.3 g per plant, and seed protein content from 18.3 to 32.2% of seed dry weight. In winter sowing (i.e. Pop9 in 2008, 2009 and 2010), SW varied from 5.1 to 119.1 g per plant; SN from 23.8 to 586.4, TSW from 101.4 to 258.7 g per plant, and seed protein content from 19.3 to 30.3% of seed dry weight. The environment showed a highly significant effect on all traits in all populations, except SPC in Pop9. Genotype-by-environment (GxE) interactions were significant for Pop3, Pop5, and Pop9 (Supplementary Table S2) but not for Pop4. These effects were partly associated with different responses to environmental conditions such as diseases impacting plants in 2004 or the sowing period for Pop9 (Supplementary Table S2 and Table S1). However, a major determinant of these interactions was that the population composition was changed in Pop3, Pop5, and Pop9 in 2011 as compared to other years since tall plants carrying the *Le* allele were discarded for this trial (see “Material and methods”; Supplementary Fig. S3). In the glasshouse, SN and SW were significantly lower than in the field, especially for Pop10, unlike TSW and SPC that were stable across environments (Supplementary Table S1). Several parents of RIL populations were chosen for their high seed protein content i.e. Caméor, VavD265 and China. Progenies of the crosses involving two of these parents (i.e. Pop3, Pop9) showed high range of SPC up to > 30%. Transgressive segregants were notable for SPC, even in progenies where parents were not chosen for their high SPC such as in the Pop8 ‘Kazar’ x ‘Melrose’ where some of the RILs show SPC values up to 29% (Supplementary Table S1 and supplementary Fig. S1).

### Correlations among yield components and seed protein content

**Figure 1:**
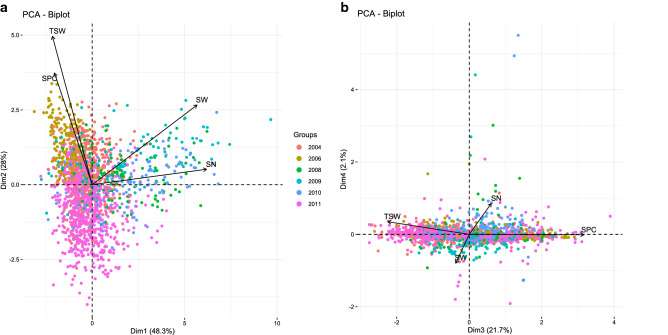
(**a**, **b**) Principal component analysis of phenotypic traits of Pop3 to Pop10 observed on the field trials between 2004 and 2011 at INRAE Dijon (**a** axis 1&2, **b** axis 3&4). *SN* seed number per plant, *SW* seed weight per plant (g), *TSW* thousand seed weight (g) and *SPC* seed protein content (% of seed dry weight).

**Figure 2:**
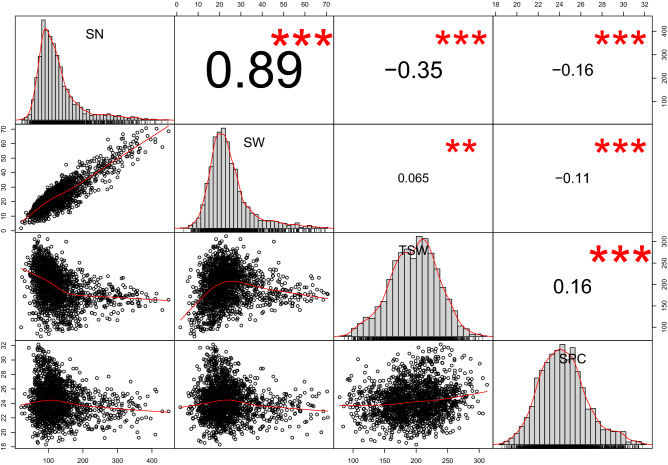
Pearson correlation coefficients between phenotypic traits recorded from 2004 to 2011 in Pop3 to Pop10 on the field trials at INRAE Dijon. *SN* seed number per plant, *SW* seed weight per plant (g), *TSW* thousand seed weight (g) and *SPC* seed protein content (% seed dry weight). *, ** and *** significant correlation at the *P* < 0.05, *P* < 0.01 and *P* < 0.001 probability level, respectively.

A principal component analysis of mean values for each line in each environment highlighted the wide phenotypic variability among the pea populations (Fig. 1). Axis 1 explained 48.3% of the phenotypic variance based mainly on SN and SW, and in a lesser extent, on TSW and SPC. Axis 2 explained 28% of the phenotypic variance and represented mainly TSW and SPC variability (Fig. 1a), and SW in a lesser extent. Axis 3 explained 21.7% of the phenotypic variance and opposed SPC and TSW (Fig. 1b). Accordingly, the overall correlation coefficients between SW, SN and TSW revealed that SW was highly correlated with SN (Pearson r = 0.89). Besides, TSW was significantly negatively correlated with SN (r = − 0.35) (Fig. 2). However, correlations between SW, SN and TSW slightly differed among populations and environments: SN was in all cases highly positively correlated with SW; TSW was positively correlated with SW in most cases except for Pop9 for which the correlation was negative in 2009 in autumn sowing, not significant in 2008 and 2010, and positive in 2011 in spring sowing, like the other populations. In Pop9, seed weight and seed number were lower in spring sowing than in autumn sowings (Supplementary Table S1). In most cases, SN and TSW were negatively correlated except in Pop8 in 2011 (Supplementary Fig. S2). The correlation between SPC and SW was slightly negative overall (Pearson r = − 0.11) (Fig. 2) but inconsistent across populations and environments. It ranged from significantly negative for Pop9 in 2008 (r = − 0.47) to significantly positive for Pop3 in 2011 and Pop10 in 2008 (r = 0.33). Likewise, SPC was significantly positively correlated with TSW (r = 0.16) except for Pop6, Pop8, and Pop9 in 2011 (Supplementary Fig. S2). TSW were lower in these populations (i.e. Pop6, Pop8 and Pop9) in 2011. In general, SPC was significantly negatively correlated with SN (r = − 0.16), except for Pop9 in 2011 and Pop10 in 2009.

### Multi-population QTL and metaQTL identification

**Figure 3:**
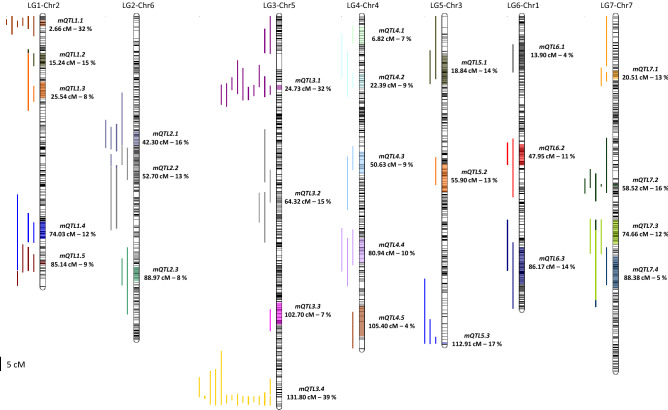
Mapping of 27 metaQTL detected for seed weight (SW), seed number (SN), thousand seed weight (TSW) and seed protein content (SPC). Position (cM) and the maximum of phenotypic variance explained for each metaQTL (mQTL) are indicated to the right of the linkage groupe (LG). Confidances intervals are represented by the color on the linkage group. The lines on the left on the linkage group indicate the each QTL position on the consensus map. Color of the line indicates membership to the metaQTL.

A total of eighty-nine multi-population QTL were identified: 17 QTL for SW explaining from 4 to 30% of SW variance, 16 QTL for SN explaining from 4 to 39% of SN variance, 35 QTL for TSW explaining from 5 to 32% of TSW variance and 21 QTL of SPC explaining from 4 to 22% of SPC variance (Supplementary Table S3). For all traits, alleles having positive effects had different parent origin, no parent bringing all positive effect for a given trait. The meta-analysis of these QTL defined a number of metaQTL regions encompassing from one to fifteen multi-population QTL and reducing their confidence intervals: 27 metaQTL were identified for yield-related traits and seed protein content (Table 1, Fig. 3 and supplementary Table S4). 4 out of 7 metaQTL detected for SW were consistently detected in two to five environments (mQTL1.5, mQTL2.1, mQTL3.1, mQTL3.4), 10 out of 16 for TSW in two to five environments (mQTL1.1, mQTL1.3, mQTL1.4, mQTL2.2, mQTL3.1, mQTL3.4, mQTL4.4, mQTL5.1, mQTL5.3, mQTL6.3, mQTL7.2), 6 out of 12 for SPC in two to four environments (mQTL1.1, mQTL3.2, mQTL3.4, mQTL4.2, mQTL4.3, mQTL7.1) and only 2 out of 10 for SN in three to five environments (mQTL3.1, mQTL3.4). Two metaQTL (mQTL3.1 and mQTL3.4) controlling all the traits were consistently detected across environments. mQTL3.1 mapped at 24.73 cM on LG3-Chr5 with a confidence interval (CI) of 1.69 cM controlled the phenotypic variation of SN, SW, TSW and SPC in five environments. On the same chromosome, mQTL3.4 at 131.8 cM with a CI of 0.03 cM controlled SN, SW, TSW and SPC in five environments. MetaQTL mQTL1.4, mQTL1.5, mQTL2.1, mQTL2.3 and mQTL7.2 controlled yield and its components. Six metaQTL controlled a specific yield trait and the seed protein content: mQTL4.2, mQTL4.4, mQTL5.3, mQTL6.2 controlled TSW and SPC, mQTL3.2 controlled SW and SPC and mQTL7.1 controlled SN and SPC. Four metaQTL specific of TSW were detected in two to three environments: mQTL2.2 on the LG2-Chr6 at 52.70 cM (CI = 9.45 cM) in three environments, mQTL1.3 (LG1-Chr2 at 25.54 cM, CI = 5.66 cM), mQTL5.1 (LG5-Chr3 at 18.84 cM, CI = 10.29 cM) and mQTL6.3 (LG6-Chr1 at 86.17 cM, CI = 12.84 cM) in two environments. Conversely, mQTL4.3 (LG4-Chr4 at 50.63 cM, CI = 7.64 cM) and mQTL4.5 (LG4-Chr4 at 105.40 cM, CI = 10.50 cM) controlled exclusively SPC in two and one environments respectively.

**Table 1:** MetaQTL parameters detected for seed weight (SW), seed number (SN), thousand seed weight (TSW) and seed protein content (SPC) from Pop3 to Pop11 between 2004 and 2011 in the fields environments at INRAE Dijon. For each metaQTL (mQTL), position and confidence interval on the genetic map (Tayeh et *al*.)^20^ and on the physical map (Kreplak et *al*.)^23^ are indicated. The number, the potential candidates genes and the annotation are listed.

Linkage group	Chromosome	metaQTL name	Predicted metaQTL position (cM)	Meta QTL 95% genetic confidence interval (cM)	Flanking markers (Tayeh *et al*.—Table S10)^20^	Position start–gene at start (Kreplak *et al.*)^23^		Position end–gene at end(Kreplak *et al.*)^23^		Peak position–peak gene(Kreplak *et al.*)^23^		Annotation (Kreplak *et al.*)^23^
LG1	Chr2	mQTL1.1	2.66	2.19	PsCam016921_10518_200 ; PsCam042632_26683_1732	454,467	*Psat2g000640*	5,774,049	*Psat2g006680*	2,980,750	*Psat2g004160*	Protein kinase domain
LG1	Chr2	mQTL1.2	15.24	4.92	PsCam044143_28060_1723 ; PsCam000692_604_684	14,937,233	*Psat2g016080*	31,547,380	*Psat2g026280*	23,203,491	*Psat2g021920*	Unknown gene
LG1	Chr2	mQTL1.3	25.54	5.66	PsCam001049_894_1330 ; PsCam040389_25143_344	32,549,163	*Psat2g027160*	80,822,825	*Psat2g046440*	56,661,835	*Psat2g037640*	LIM domain
LG1	Chr2	mQTL1.4	74.03	6.08	PsCam036267_21415_2674 ; PsCam046088_29624_710	372,862,478	*Psat2g145000*	401,022,163	*Psat2g166320*	387,065,449	*Psat2g156040*	Pre-rRNA-processing protein TSR2
LG1	Chr2	mQTL1.5	85.14	1.47	PsCam049764_32384_3938 ; PsCam047748_30645_563	409,802,804	*Psat2g171720*	413,236,825	*Psat2g174040*	411,495,507	*Psat2g172600*	Amino-transferase class IV
LG2	Chr6	mQTL2.1	42.30	5.06	PsCam045557_29214_1582 ; PsCam037747_22816_1252	71,913,567	*Psat6g064200*	157,742,624	*Psat6g097360*	114,863,224	*Psat6g082080*	Unknown gene
LG2	Chr6	mQTL2.2	52.70	9.45	PsCam034889_20236_728 ; PsCam050914_33467_786	88,985,653	*Psat6g070840*	290,738,342	*Psat6g148120*	189,681,633	*Psat6g111440*	Unknown gene
LG2	Chr6	mQTL2.3	88.97	4.98	PsCam050254_32847_1148 ; PsCam053661_35501_2019	369,699,084	*Psat6g183960*	404,311,257	*Psat6g203560*	388,681,088	*Psat6g193240*	Plant organelle RNA recognition domain
LG3	Chr5	mQTL3.1	24.73	1.69	PsCam001506_1254_232 ; PsCam039566_24482_378	56,764,094	*Psat5g030080*	66,228,363	*Psat5g035840*	61,462,435	*Psat5g032160*	MIR domain
LG3	Chr5	mQTL3.2	64.32	6.60	PsCam036453_21593_1599 ; PsCam038194_23240_124	191,691,654	*Psat5g107640*	239,064,211	*Psat5g133080*	215,141,118	*Psat5g121200*	Unknown gene
LG3	Chr5	mQTL3.3	102.70	7.70	PsCam006936_5161_789 ; PsCam038944_23937_299	422,221,988	*Psat5g207040*	511,578,964	*Psat5g257360*	467,013,019	*Psat5g233240*	Unknown gene
LG3	Chr5	mQTL3.4	131.80	0.03	PsCam034779_20141_1638 ; PsCam053662_35502_3991	566,380,665	*Psat5g299280*	568,296,029	*Psat5g299920*	567,365,719	*Psat5g299720*	2OG-Fe(II) oxygenase superfamily
LG4	Chr4	mQTL4.1	6.82	6.30	PsCam056984_37672_88 ; PsCam008438_5952_431	1,743,557	*Psat4g001960*	20,433,499	*Psat4g014520*	11,038,786	*Psat4g009760*	Intracellular non-membrane-bounded organelle
LG4	Chr4	mQTL4.2	22.39	4.80	PsCam005173_3921_366 ; PsCam038342_23380_122	30,049,059	*Psat4g021160*	54,024,236	*Psat4g036960*	42,061,945	*Psat4g028480*	Xanthine/uracil/vitamin C permease
LG4	Chr4	mQTL4.3	50.63	7.64	PsCam043089_27118_711 ; PsCam059345_39539_779	117,446,584	*Psat4g071040*	178,101,781	*Psat4g094840*	147,602,594	*Psat4g084600*	2OG-Fe(II) oxygenase superfamily
LG4	Chr4	mQTL4.4	80.94	8.36	PsCam044314_28211_1843 ; PsCam035946_21100_283	229,359,999	*Psat4g123400*	315,404,846	*Psat4g161720*	271,800,566	*Psat4g139360*	SRP54-type protein + GTPase domain
LG4	Chr4	mQTL4.5	105.40	10.50	PsCam001458_1216_3409 ; PsCam046293_29776_939	320,544,838	*Psat4g164800*	417,776,284	*Psat4g205800*	369,227,093	*Psat4g181120*	BURP domain
LG5	Chr3	mQTL5.1	18.84	10.29	PsCam037375_22461_2049 ; PsCam044994_28731_4247	13,496,546	*Psat3g004680*	67,064,585	*Psat3g031920*	40,364,479	*Psat3g017840*	Unknown gene
LG5	Chr3	mQTL5.2	55.90	9.59	PsCam034798_20158_236 ; PsCam012541_8505_720	144,455,569	*Psat3g068320*	208,140,583	*Psat3g105520*	176,232,116	*Psat3g085120*	MatE
LG5	Chr3	mQTL5.3	112.91	0.37	PsCam009698_6488_1702 ; PsCam004880_3686_1478	434,277,272	*Psat3g205320*	436,339,177	*Psat3g207360*	435,352,873	*Psat3g206400*	Alpha/beta hydrolase fold
LG6	Chr1	mQTL6.1	13.90	10.41	PsCam042987_27022_1291 ; PsCam023473_13286_428	3,521,432	*Psat1g002480*	63,895,910	*Psat1g041160*	33,786,851	*Psat1g024160*	Ammonium Transporter Family
LG6	Chr1	mQTL6.2	47.95	7.36	PsCam035272_20467_4972 ; PsCam043936_27889_1794	118,331,306	*Psat1g074560*	240,558,639	*Psat1g122920*	179,602,535	*Psat1g101120*	AP2 domain
LG6	Chr1	mQTL6.3	86.17	12.84	PsCam050644_33215_1066 ; PsCam037643_22718_391	311,313,139	*Psat1g162520*	367,259,508	*Psat1g218360*	339,253,643	*Psat1g187800*	Glutaredoxin
LG7	Chr7	mQTL7.1	20.51	2.87	PsCam020840_11620_1382 ; PsCam044103_28028_519	51,210,245	*Psat7g031600*	69,196,947	*Psat7g040040*	60,542,282	*Psat7g036360*	SBP domain
LG7	Chr7	mQTL7.2	58.52	0.96	PsCam051665_34135_1538 ; PsCam000487_426_1043	202,181,919	*Psat7g122680*	211,451,196	*Psat7g127960*	206,761,590	*Psat7g125120*	PLAC8 family
LG7	Chr7	mQTL7.3	74.66	8.38	PsCam056507_37317_287 ; PsCam027452_16087_1460	319,631,535	*Psat7g166760*	371,178,765	*Psat7g195480*	345,409,046	*Psat7g184840*	Probable lipid transfer
LG7	Chr7	mQTL7.4	88.38	10.93	PsCam050926_33478_1100 ; PsCam042627_26678_74	359,853,588	*Psat7g190640*	459,632,407	*Psat7g229640*	409,725,211	*Psat7g206160*	PPR repeat

Linkage group	Chromosome	metaQTL name	Number of genes in confidence interval (Kreplak *et al.*)^23^	Candidates genes in confidence interval (Kreplak *et al.*)^23^	Initial number of QTL	*P* value range of initial QTL	*R*² range of initial QTL	Max position range of initial QTL (cM)	QTL assignment to metaQTL (Caméor allele effect sign)			
LG1	Chr2	mQTL1.1	141	Agps2 ; Subtilase family	6	5.38–38.44	0.08–0.32	0.00–4.90	SN04+, SPC04-, SPC06-, TSW04-, TSW06-, TSW11-			
LG1	Chr2	mQTL1.2	235		1	9.57	0.15	15.20	TSW06-			
LG1	Chr2	mQTL1.3	445	GlutamineSynthetase	2	4.85–5.77	0.05–0.08	25.50–26.10	TSW04-, TSW08-			
LG1	Chr2	mQTL1.4	448		3	4.40–6.80	0.06–0.12	69.60–75.40	SN09+, TSW08-, TSW09-			
LG1	Chr2	mQTL1.5	56	Afila	3	5.53–9.34	0.08–0.09	82.70–85.90	SN04+, SW04+, SW06+			
LG2	Chr6	mQTL2.1	751	GlutamineSynthetase	4	4.66–10.39	0.04–0.16	39.20–44.60	SN06-, SW04-, SW06-, SW11-			
LG2	Chr6	mQTL2.2	1761	Cwi1	3	6.90–8.37	0.08–0.13	51.50–57.40	TSW04-, TSW08-, TSW09-			
LG2	Chr6	mQTL2.3	457	Ppgm	2	4.01–9.45	0.04–0.08	87.40–98.40	SW11+, TSW11-			
LG3	Chr5	mQTL3.1	135		12	7.02–29.81	0.06–0.32	3.50–27.80	SN08-, SN09-, SN11-, SPC11-, SW08-, SW09-, SW11-, TSW04+, TSW06+, TSW08+, TSW09+, TSW11+			
LG3	Chr5	mQTL3.2	583	PA2 ; RubiscoActivase	4	4.60–7.03	0.08–0.15	56.00–73.30	SPC06+, SPC08+, SPC10+, SW06-			
LG3	Chr5	mQTL3.3	1172	PepC	1	7.69	0.07	102.70	TSW04-			
LG3	Chr5	mQTL3.4	17	AUX/IAA family	15	5.59–37.90	0.07–0.39	127.90–133.50	SN04-, SN06-, SN08-, SN10-, SN11-, SPC06+, SPC010+, SPC11+, SW04-, SW06-, SW08-, SW10-, SW11-, TSW04-, TSW08+			
LG4	Chr4	mQTL4.1	290		1	4.84	0.07	6.50	SW08+			
LG4	Chr4	mQTL4.2	364		3	4.64–6.21	0.04–0.09	18.40–22.80	SPC04-, SPC11-, TSW06-			
LG4	Chr4	mQTL4.3	546		2	5.78–7.64	0.07–0.09	49.30–57.20	SPC04+, SPC06+			
LG4	Chr4	mQTL4.4	876	Leg	3	5.97–9.99	0.07–0.10	77.40–84.10	SPC06-, TSW04+, TSW11-			
LG4	Chr4	mQTL4.5	922		1	4.34	0.04	105.40	SPC11+			
LG5	Chr3	mQTL5.1	640	SucroseSynthase	2	5.88–8.57	0.06–0.14	10.40–21.10	TSW04-, TSW06-			
LG5	Chr3	mQTL5.2	855	Leg	1	8.13	0.13	55.90	SPC06+			
LG5	Chr3	mQTL5.3	51	Rubisco	3	6.68–11.17	0.06–0.17	97.20–113.30	SPC06-, TSW04-, TSW06-			
LG6	Chr1	mQTL6.1	884		1	4.05	0.04	13.90	SN11+			
LG6	Chr1	mQTL6.2	1079	Gbsts2	2	3.64–7.97	0.07–0.11	47.70–49.60	SPC10-, TSW11+			
LG6	Chr1	mQTL6.3	1292	GlutamineSynthetase	2	4.31–5.81	0.10–0.14	81.30–94.20	TSW06-, TSW10-			
LG7	Chr7	mQTL7.1	199		3	4.25–6.83	0.07–0.13	10.10–20.80	SN08-, SPC08+, SPC10+			
LG7	Chr7	mQTL7.2	126	Ptrans	5	4.78–17.24	0.07–0.16	45.20–58.60	SN06-, TSW04+, TSW06+, TSW09+, TSW11+			
LG7	Chr7	mQTL7.3	668		3	7.01–9.97	0.07–0.12	71.40-77.80	SPC08-, SN11+, SW11+			
LG7	Chr7	mQTL7.4	884		1	4.89	0.05	88.60	TSW04-

MetaQTL were located on all linkage groups (Fig. 3). Taking advantage of the newly published genome sequence of pea^23^ we could identify the position and the genes underlying metaQTL and highlight potential candidate genes. For example, 17 genes were identified in the confidence interval of mQTL3.4 (CI = 0.03 cM), 51 genes in the confidence interval of mQTL5.3 (CI = 0.37 cM) and 56 genes in the confidence interval of mQTL1.5 (CI = 1.47 cM) (Table 1). However, even for the shortest confidence intervals, the number of underlying genes is still high: 126 genes were identified in the confidence interval of mQTL7.2 (CI = 0.96 cM), 135 genes for mQTL3.1 (CI = 1.69 cM), 141 genes are underlying for mQTL1.1 (CI = 2.19 cM) and 199 genes for mQTL7.1 (CI = 2.87 cM).

## Discussion

### Diversity and correlation of seed traits in pea

Our study shows a large range of genetic variation for seed productivity and quality traits carried by cultivated pea varieties and their progenies, suggesting avenues for breeding (Supplementary Table S1). Our results confirm that the seed weight per plant is highly positively correlated with the seed number per plant, and generally positively correlated with the thousand seed weight (Fig. 2, Supplementary Fig. S2). The relationship between seed weight per plant and seed protein content is varied: for example, the correlation was slightly positive within Pop4 and was negative within Pop9. Our study reveals that in pea, by contrast to cereals^24,25^, the relationship between seed yield per plant and seed protein content is not necessarily negative but depends on the environment and the genetic background.

### Genetic control of traits

QTL detection is impacted by many factors, including the genetic background, the population size, the quality and density of the genetic map and the statistical method used for detection. Meta-analysis of QTL initially developed by Goffinet and Gerber^26^ and implemented by Veyrieras et al*.*^22^ and Sosnowski et al*.*^27^ is an efficient approach to identify consensus QTL positions among experiments and reduce their confidence intervals. The method has been used in a wide range of species^28–35^ and was applied in the present study for a large set of phenotypic data obtained from nine RIL populations and seven environments (Table 2). A total of 89 QTL explaining a part of phenotypic variation were detected across the seven pea chromosomes (Table 1).

**Table 2:** RIL populations for QTL meta-analysis. For each population, the population size (number of individuals (ind.)), the generation, the number of lines genotyped and phenotyped in each environment at INRAE Dijon are indicated.

Population	Cross	Population size and generation	Number of genotyped lines	Number of mapped SNP markers on the framework map (Tayeh et al*.*)^20^	Number of phenotyped lines									Main QTL	References
					2004 in field	2006 in field	2008 in field	2008 in glasshouse	2009 in field	2009 in glasshouse	2010 in field	2011 in field	2011 in glasshouse		
Pop3	VavD265 x Cameor	210 ind.—F_6:8_	176	1176	175	152	–	–	–	–	–	84*	–	Seed quality and productivity, other agronomic traits	Bourgeois et al.^8^, Tayeh et al*.*^20^, Bordat et al*.*^47^
Pop4	Ballet x Cameor	210 ind.—F_6:8_	159	484	159	134	–	–	–	–	–	73	–	Seed quality and productivity, other agronomic traits, root development	Bourgeois et al*.* ^8^, Tayeh et al*.*^20^, Bordat et al*.*^47^, Bourion et al*.*^50^
Pop5	VavD265 x Ballet	210 ind.—F_6:8_/F_6:9_	168	937	155	–	–	–	–	–	–	71*	–	Seed quality and productivity, other agronomic traits	Bourgeois et al.^8^, Tayeh et al.^20^, Bordat et al*.*^47^
Pop6	Cameor x Melrose	283 ind.—F_8_	120	856	–	–	–	–	–	–	–	182	–	Seed quality, frost tolerance, *M. pinodes* resistance	Tayeh et al*.*^20^
Pop7	Kazar x Cameor	280 ind.—F_8_	84	411	–	–	–	–	–	–	–	90	–	Seed quality, frost tolerance, *M. pinodes* resistance	Tayeh et al*.*^20^
Pop8	Kazar x Melrose	220 ind.—F_8:10_	118	496	–	–	–	–	–	–	–	142	–	Seed quality, frost tolerance, *M. pinodes* resistance	Tayeh et al*.*^20^
Pop9	China x Cameor	129 ind.—F_6:8_	124	913	–	–	114	–	93	–	114	61*	–	Seed quality and productivity, frost tolerance	Klein et al.^10^, Tayeh et al*.*^20^, Deulvot et al.^48^, Duarte et al*.*^49^
Pop10	Cameor x Sommette	146 ind.—F_6_	144	653	–	–	–	143	–	142	–	85	–	Nitrogen nutrition	Tayeh et al*.*^20^
Pop11	Cameor x Cerise	120 ind.—F_3_	120	231	–	–	–	115	–	–		–	112	Seed size, seed quality	Tayeh et al*.*^20^
		TOTAL	1213	1869 markers on the consensus map—794.9 cM	489	286	114	258	93	142	114	788	112		

*Only short lines carrying the le allele were selected from the population to be tested in this trial.

The meta-analysis of these QTL revealed 27 consensus QTL, or metaQTL; each metaQTL corresponding to one to 15 initial QTL (Table 1). Most metaQTL were consistently detected in different environments, in spite of significant environmental and GxE effects. Fifteen metaQTL controlled more than one trait. When different traits were controlled by the same metaQTL, allelic effects were of the same sign for SN and SW QTL (5 metaQTL out of 5) and of opposite sign for SN or SW and TSW (5 metaQTL out of 6), similarly to the correlation study of these traits. Allelic effects of SPC QTL were of the same sign with SN and SW QTL in one case out of 5.

A survey of the literature allowed, when common markers where used or when markers’ genomic positions were known, to identify likely correspondence between the metaQTL from this study and QTL previously detected for seed yield, seed yield components and seed protein content in different pea populations and in different environments (supplementary Table S5). For example, mQTL1.1 was shown to control SPC and TSW in several environments in the present study, and was also detected by Moreau et al*.*^12^ as a locus controlling SW and by Gali et al.^13^ as a region controlling SPC; mQTL2.1 controlled SW in 3 environments in the present study as well as in Tar’an et al*.*^5^; mQTL3.1 controlled TSW in 5 environments in the present study as well as in Klein et al.^8^, Burstin et al.^7^ and Gali et al.^13^; mQTL3.2. controlled SPC in three environments in the present study as well as in Gali et al.^13^; mQTL3.4 controlled SW in 5 environments in the present study and also in Klein et al.^8^, Burstin et al.^7^ and Gali et al.^13^; Some metaQTL only controlled one trait in one environment in the present study but were also detected in other studies, such as mQTL5.2, a QTL of SPC also in Burstin et al.^7^ and Gali et al.^13^ (Table S4). Because the genetics of seed yield and seed protein content have been widely studied in soybean, we also searched for any related QTL to the pea metaQTL. Orthologous genes for the ones harbouring the peak markers of the metaQTL were identified and QTL within 50 kb around these genes were searched. This further reinforced the interest of the SW QTL mQTL2.1 and mQTL4.1, of the TSW QTL mQTL6.2 and mQTL2.2 and the SPC QTL mQTL4.3 and mQTL4.5 which corresponded to QTL of the same traits in soybean^34,36–40^ (Supplementary Table S5).

### Candidate genes

The meta-analysis method allowed to locate with more confidence QTL regions associated with seed productivity and quality traits. In some cases, the number of the genes underlying the metaQTL confidence intervals was narrow. The confidence intervals of metaQTL mQTL3.4, mQTL5.3, mQTL1.5, mQTL7.2, mQTL3.1 and mQTL1.1 include 17, 51, 56, 126, 135, 141 genes, respectively. mQTL3.4 and mQTL1.5 encompass, respectively, the *Le* and *Afila* regions previously described as controlling a number of traits in pea^7^. *Le* is *Psat5g299720* and encodes a 2OG-Fe(II) oxygenase involved in Gibberrelin biosynthesis. Selecting short plants for Pop3, 5, and 9 in 2011 did not prevent to detect this QTL because this gene also segregates in Pop6 and Pop8. The region also includes *Psat5g299400*, a gene belonging to the AUX/IAA family putatively involved in early response to auxin. The mQTL1.5 region also contains several genes encoding transcription factors expressed in flowers (*Psat2g173120*, *Psat2g173160*) or seeds (*Psat2g173360, Psat2g173520, Psat2g173880*) and genes encoding an aminotransferase (*Psat2g172800*) and a malate transporter (*Psat2g173480*) that could be relevant candidates for this metaQTL. The mQTL5.3 region includes two genes encoding phosphatidylethanolamine-binding proteins which expression peaks in the upper leaves as revealed by the Pea Gene Atlas^41^. The phosphatidylethanolamine-binding proteins in soybean and Arabidopsis thaliana are involved in flowering time, plant architecture and seed germination^42^. The mQTL1.1 region includes the locus AGPS2, a gene encoding ADP-glucose pyrophosphorylase (*Psat2g005160*) previously reported to be associated with seed size QTL in pea^16^, as well as a gene encoding a subtilase (*Psat2g005680)* expressed in flowers and pods^41^ different to subtilase gene (*Psat0s1712g0120*) in D’Erfurth et al.^19^. The mQTL3.1 region contains a gene encoding a Phosphoenolpyruvate carboxylase (*Psat5g031640*) expressed in above ground vegetative and reproductive tissues^41^ that could impact C assimilation and partitioning in the plant^43,44^. The mQTL3.1 region encompass two tandem genes (*Psat7g127600, Psat7g127680*) putatively encoding Kelch motif proteins. Interestingly, Kelch Motif-containing serine/threonine protein phosphatase was associated with a seed size QTL in rice^45^.

The present study pinpointed several robust metaQTL of seed yield and seed protein content in pea and proposed some candidate genes. This useful knowledge for marker assisted breeding, highlights the position and function of underlying genes to discover the causal polymorphisms of QTL in pea.

## Materials and methods

### Plant material and field experiments

A total of 1213 recombinant inbred lines (RIL) derived from nine mapping populations (Pop3 to Pop11) were developed by single seed descent (SSD). Pop3 to Pop10 are eight advanced inter-connected biparental RIL populations with six of them having Cameor as a common parent. Pop11 is an F_3_ population obtained from a cross between Cameor and Cerise. Passport data and phenotypic information relative to the parental lines of mapping population are described in Table S6 and in Tayeh et al.^20^. The population size, the generation, the number of lines genotyped and phenotyped used for QTL meta-analysis are indicated in Table 2. Phenotypic variability of morphological traits of RIL populations are given in supplementary Fig. S3. Pop3 to Pop10 and parental lines were evaluated in six field environments at INRAE Dijon, Domaine d’Epoisses, Bretenière, France (47°14′N, 5°05′E, altitude 210 m) between 2004 and 2011 in spring sowing, except for Pop9 in 2008, 2009, 2010 evaluated in winter sowing (Table 2). In 2011, a subset of the populations was sown. In Pop3, Pop5, and Pop9 where the *Le* gene controlling internode length segregates, only short lines were sampled for the trial in order to limit the shading of tall plant plots on their neighbours (supplementary Fig. S3). In Pop6 and Pop8, the *Le* gene also segregates but the lines *Le* type information was lacking before sowing. Furthermore, Burstin et al.^7^ have shown the major effect of this gene in the variation of seed traits and sampling only short plants intended to improve the detection of other QTL regions. Field experiments were carried out using a randomized complete block design. Each plot consisted of twenty-five seeds sown in a row of two meters long, with one meter spacing between two adjacent rows. Plants were grown against trellises. Weeds, insects and diseases were controlled chemically. At maturity, a sample of ten plants per line was harvested and Seed Number per plant (SN), Seed Weight per plant (SW, gram), Thousand Seed Weight (TSW, gram) were measured and Seed Protein Content (SPC, percentage of seed dry weight) was analysed by near-infrared spectrometry as described in Burstin et al.^7^.

Pop10 in 2008–2009 and Pop11 in 2008, 2009 and 2011 were phenotyped in glasshouses at INRAE Dijon, France (47° 32′ N, 5°07′ E, altitude 245 m) (Table 2). Two replicates of two plants per RIL were grown in 4-L pots filled with a 1:1 (v/v) mixture of sterilized atapulgite and clay balls (2–6 mm diameter) with a nitrate content (10 mM). The temperature and minimal day length were controlled (22° C/16 °C, 16-h photoperiod). SN, SW and TSW were measured at maturity per plant.

### Statistical analysis

Statistical analysis of each environment dataset was carried out using R software v3.3 and v3.6^46^. ANOVA were performed using the “aov” function in R, to determine the significance levels of the genotype and replication effects. The statistical model was: Yijk = µ + gi + rj + bk/j + eijk where Yijk is the value of the trait for genotype i in block k of the replicate j, µ the general mean, gi the genotypic effect, rj the replicate effect, bk/j the block k effect in the replicate j and eijk the residual. In the case of the populations phenotyped in several environments (Table 2), environment and genotype-by-environment interaction effects were added to the linear model of ANOVA. Broad sense heritability (h^2^) was estimated from ANOVA by h^2^ = σ^2^g / [σ^2^g + (σ^2^e/n)] with σ^2^g the genetic variance, σ^2^e the residual variance and n the number of replicates. Normality of residuals and homogeneity of variances were checked using Shapiro–Wilk and Bartlett’s test (*P* ≥ 0.05). RILs’ adjusted means calculated using the “lsmeans” library were used for QTL analysis. Pearson correlation coefficients between the traits for all environments were calculated from RILs adjusted means using “chart.correlation” function and “PerformanceAnalytics” library. Frequency distributions from RILs adjusted means using “car” library and the “hist” function. Principal component analysis from RILs adjusted means was performed using “fviz_pca” function and “factoextra” library.

### Genotyping and QTL analyses

The 1213 RIL derived from nine mapping populations (Pop3 to Pop11) were previously genotyped using the GenoPea 13.2 K SNP Array and used for the construction of individual genetic maps and a consensus map as described in Tayeh et al.^20^. The framework consensus map included 1869 markers and had a total length of 794.9 cM Haldane. The references of these population^8,10,20,47–50^ are listed in Table 2.

QTL composite interval mapping was carried out using the iterative QTL mapping method (iQTLm) of the MCQTL software v5.2.4^21^. Cofactor selection and QTL detection *P*value thresholds were determined after 1000 permutation tests on all traits, for a global genome-wide type I risk of 10% for cofactor selection, and 5% for QTL detection. Cofactors were searched by forward regression, using a threshold of *P* value = 3.50. QTL were searched by iQTLm, using a threshold of *P* value = 3.80. For each environment, MultiPop detection was performed using the genotyping from GenoPea 13.2 K SNP Array and the consensus genetic map (1869 markers—794.9 cM) developed by Tayeh et al.^20^. Model additive and interpop connected was used in Multipop function for the populations in the same environment. The *P*value, global R^2^, individual R^2^, confidence interval and allelic effect at each QTL were estimated for each trait and used for metaQTL analyses.

### MetaQTL analyses

Meta-analyses were performed using BioMercator version 4.2 software^27^. Meta-analysis was implemented on each chromosome to estimate the number, the position, the probability of individual QTL belonging to the metaQTL and 95% confidence interval (CI) of the each metaQTL. QTLProj command enabled the homothetic projection of the positions and the confidence intervals of the individual QTL onto the consensus map. QTLClust command performed the clustering of the projected QTL referring to the same trait on a given chromosome into all possible numbers of hypothetic clusters. This command determined the best clustering model based on the following criteria^22^: AIC (Akaike Information Criterion), AICc, AIC3, BIC (Bayesian information criterion) and AWE (Average Weight of Evidence). The best QTL model was selected when values of the model selection criteria were the lowest in at least three of the five models. It corresponds to the optimal number of clusters that best explain the observed QTL distribution along the consensus chromosome map. Finally, the QTLClustInfo command provided the number of metaQTL for each chromosome, the better position, the confidence interval and the contribution of each individual QTL^20^. The metaQTL map was drawn using BioMercator software^27^.

### Candidate genes, functional annotation and expression

QTL flanking markers were positioned on ‘Cameor’ genome sequence^23^ through BLAST search and annotated genes in the QTL interval were retrieved and listed. QTL and orthologous genes in soybean were identified using USDA-ARS Soybean Genetics Database, SoyBase^36^ (https://www.soybase.org). Pea gene expression was obtained from Pea RNA-seq Gene Atlas^41^ (https://bios.dijon.inra.fr/FATAL/cgi/pscam.cgi).

## Supplementary information

Supplementary Figure 1.

Supplementary Figure 2.

Supplementary Figure 3.

Supplementary Table 1.

Supplementary Table 2.

Supplementary Table 3.

Supplementary Table 4.

Supplementary Table 5.

Supplementary Table 6.

Supplementary Legends.

## Data Availability

The datasets generated and analysed during the current study are available from the corresponding author on reasonable request.
