# Supplementary material for: Meta-analysis of QTL reveals the genetic control of yield-related traits and seed protein content in pea
Source: Sci Rep. 2020 Sep 28;10:15925. doi: 10.1038/s41598-020-72548-9 (PMC7522997; doi:10.1038/s41598-020-72548-9)
Supplement: Supplementary file 1 — Supplementary Figure 1. [file 41598_2020_72548_MOESM1_ESM.pdf]

Meta-analysis of QTL reveals the genetic control of yield-related traits and seed protein content in pea

Anthony Klein[1*], Hervé Houtin[1], Céline Rond-Coissieux[1], Myriam Naudet-Huart[1], Michael Touratier[1], Pascal Marget[2,1] and Judith Burstin[1]

[1] Agroécologie, AgroSup Dijon, INRAE, Univ. Bourgogne, Univ. Bourgogne Franche-Comté, F-21000 Dijon, France

[2] INRAE, UE 0115 DIJ Domaine Expérimental d'Epoisses. Centre de recherche Bourgogne-Franche-Comté, F-21110 Breteniere, France

*** Correspondence:**
anthony.klein@inrae.fr

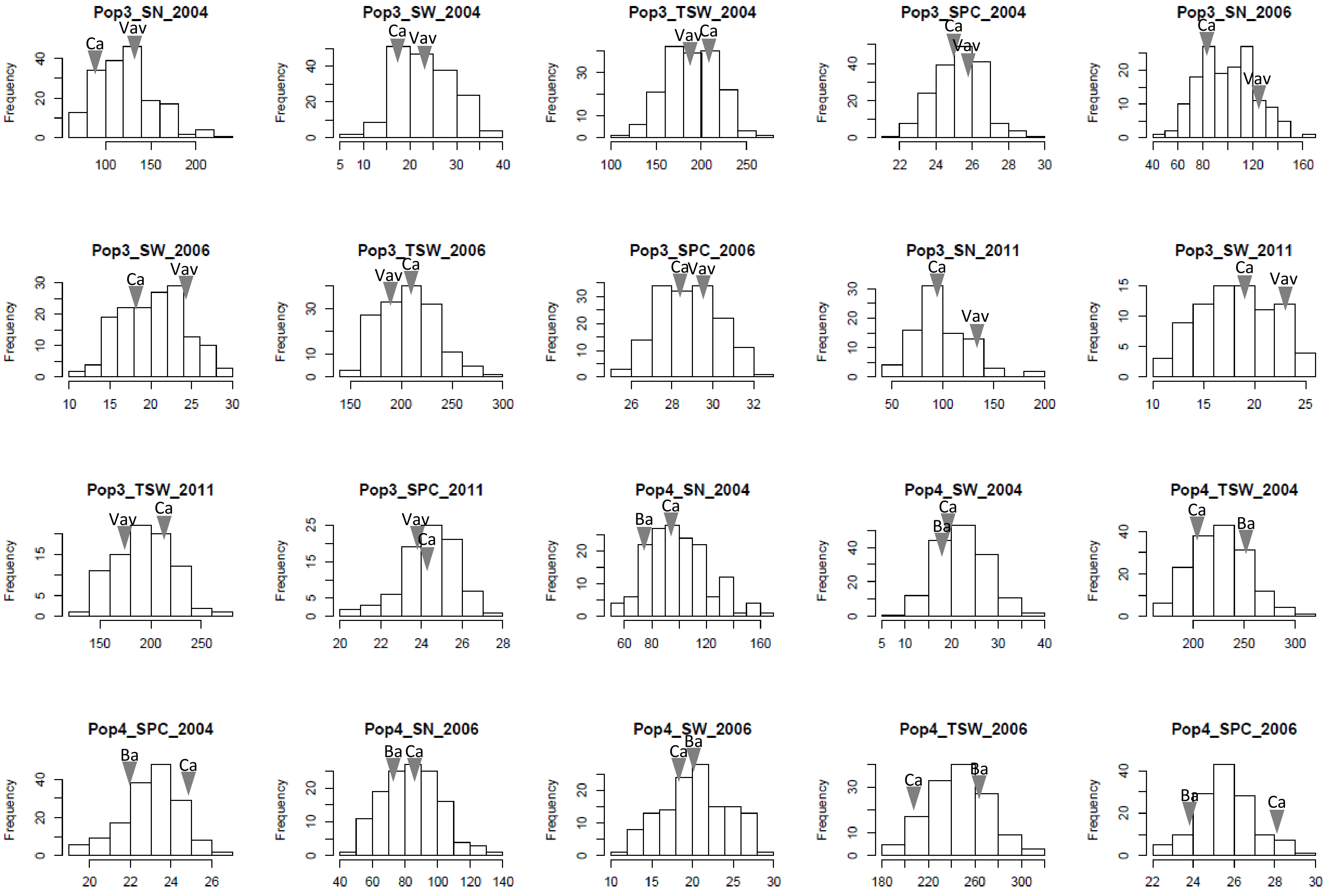
Pop3_SN_2004
Ca
Vav
Pop3_SW_2004
Ca
Vav
Pop3_TSW_2004
Vav
Ca
Pop3_SPC_2004
Ca
Vav
Pop3_SN_2006
Ca
Vav
Pop3_SW_2006
Ca
Vav
Pop3_TSW_2006
Vav
Ca
Pop3_SPC_2006
Ca
Vav
Pop3_SN_2011
Ca
Vav
Pop3_SW_2011
Ca
Vav
Pop3_TSW_2011
Vav
Ca
Pop3_SPC_2011
Vav
Ca
Pop4_SN_2004
Ba
Ca
Pop4_SW_2004
Ca
Ba
Pop4_TSW_2004
Ca
Ba
Pop4_SPC_2004
Ba
Ca
Pop4_SN_2006
Ba
Ca
Pop4_SW_2006
Ca
Ba
Pop4_TSW_2006
Ca
Ba
Pop4_SPC_2006
Ba
Ca
Frequency

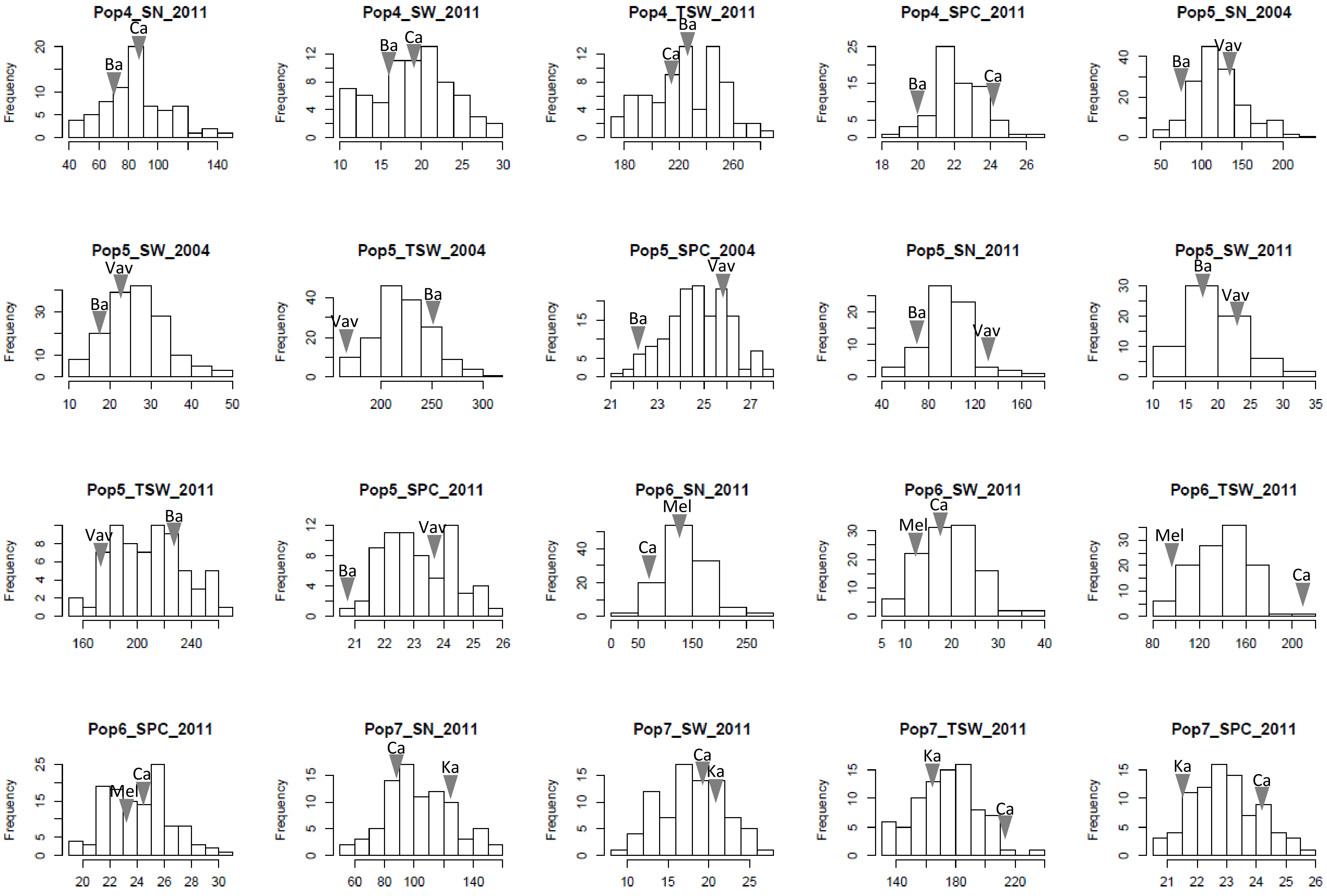
Pop4_SN_2011
Ba
Ca
Pop4_SW_2011
Ba
Ca
Pop4_TSW_2011
Ca
Ba
Pop4_SPC_2011
Ba
Ca
Pop5_SN_2004
Ba
Vav
Pop5_SW_2004
Ba
Vav
Pop5_TSW_2004
Vav
Ba
Pop5_SPC_2004
Ba
Vav
Pop5_SN_2011
Ba
Vav
Pop5_SW_2011
Ba
Vav
Pop5_TSW_2011
Vav
Ba
Pop5_SPC_2011
Ba
Vav
Pop6_SN_2011
Ca
Mel
Pop6_SW_2011
Mel
Ca
Pop6_TSW_2011
Mel
Ca
Pop6_SPC_2011
Mel
Ca
Pop7_SN_2011
Ca
Ka
Pop7_SW_2011
Ca
Ka
Pop7_TSW_2011
Ka
Ca
Pop7_SPC_2011
Ka
Ca
Frequency

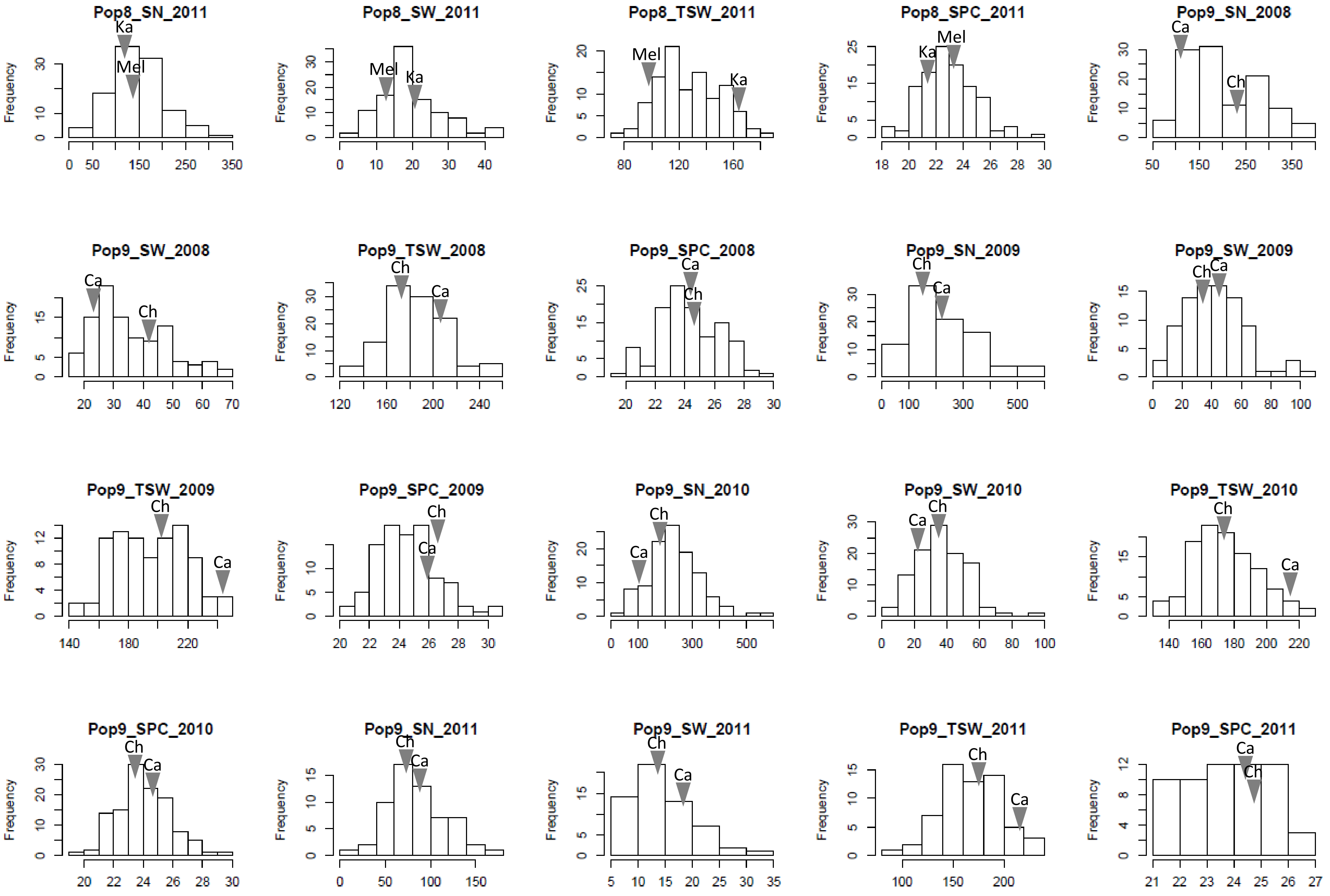
Pop8_SN_2011
Ka
Mel
Pop8_SW_2011
Mel
Ka
Pop8_TSW_2011
Mel
Ka
Pop8_SPC_2011
Ka
Mel
Pop9_SN_2008
Ca
Ch
Pop9_SW_2008
Ca
Ch
Pop9_TSW_2008
Ch
Ca
Pop9_SPC_2008
Ca
Ch
Pop9_SN_2009
Ch
Ca
Pop9_SW_2009
Ch
Ca
Pop9_TSW_2009
Ch
Ca
Pop9_SPC_2009
Ch
Ca
Pop9_SN_2010
Ch
Ca
Pop9_SW_2010
Ca
Ch
Pop9_TSW_2010
Ch
Ca
Pop9_SPC_2010
Ch
Ca
Pop9_SN_2011
Ch
Ca
Pop9_SW_2011
Ch
Ca
Pop9_TSW_2011
Ch
Ca
Pop9_SPC_2011
Ca
Ch
Frequency

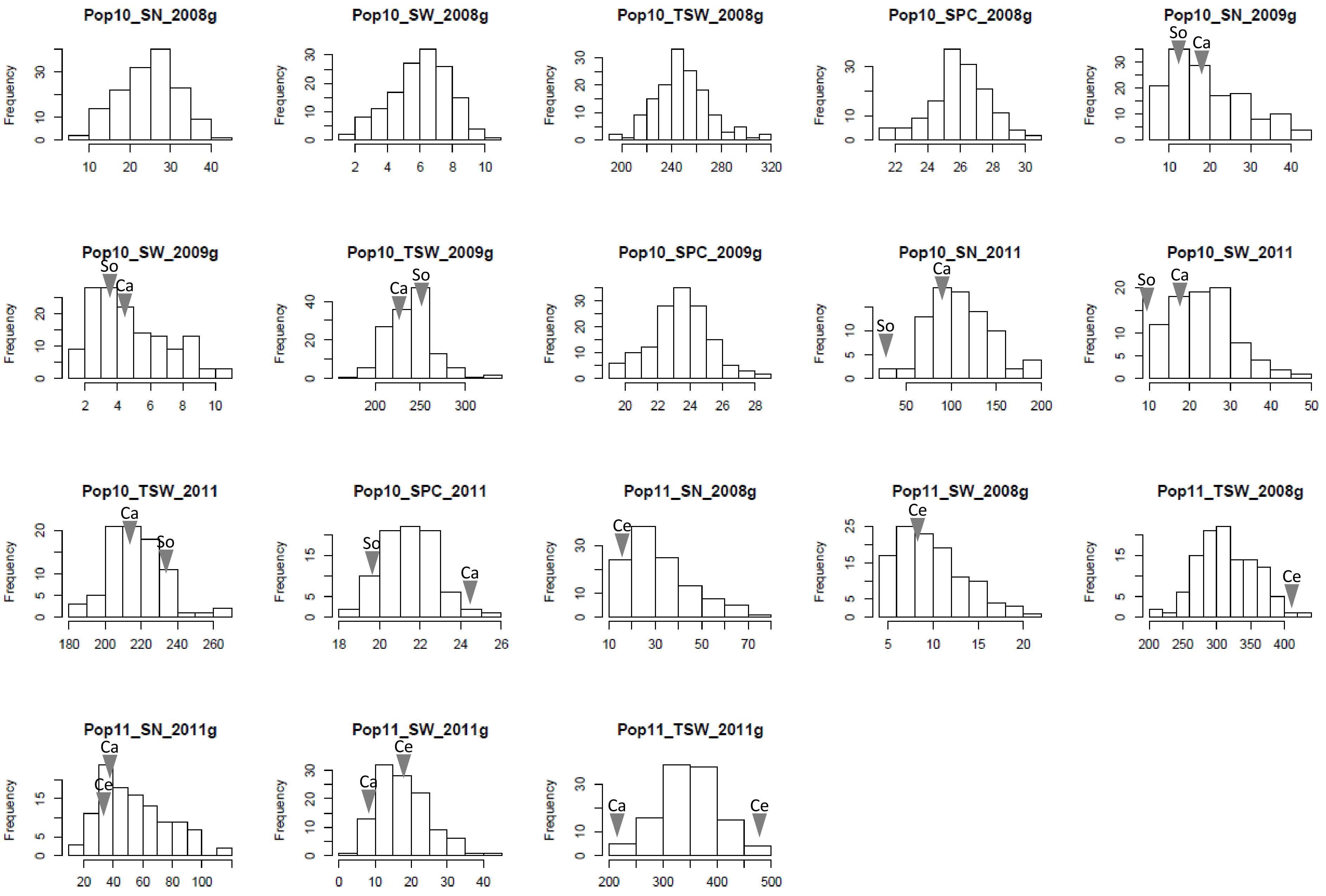
Pop10_SN_2008g
Pop10_SW_2008g
Pop10_TSW_2008g
Pop10_SPC_2008g
Pop10_SN_2009g
So
Ca
Pop10_SW_2009g
So
Ca
Pop10_TSW_2009g
Ca
So
Pop10_SPC_2009g
Pop10_SN_2011
Ca
So
Pop10_SW_2011
So
Ca
Pop10_TSW_2011
Ca
So
Pop10_SPC_2011
So
Ca
Pop11_SN_2008g
Ce
Pop11_SW_2008g
Ce
Pop11_TSW_2008g
Ce
Pop11_SN_2011g
Ca
Ce
Pop11_SW_2011g
Ca
Ce
Pop11_TSW_2011g
Ca
Ce
Frequency
