# Supplementary material for: Meta-analysis of QTL reveals the genetic control of yield-related traits and seed protein content in pea
Source: Sci Rep. 2020 Sep 28;10:15925. doi: 10.1038/s41598-020-72548-9 (PMC7522997; doi:10.1038/s41598-020-72548-9)
Supplement: Supplementary file 2 — Supplementary Figure 2. [file 41598_2020_72548_MOESM2_ESM.pdf]

# Meta-analysis of QTL reveals the genetic control of yield-related traits and seed protein content in pea

**Anthony Klein[1*], Hervé Houtin[1], Céline Rond-Coissieux[1], Myriam Naudet-Huart[1], Michael Touratier[1], Pascal Marget[2,1] and Judith Burstin[1]**

[1] Agroécologie, AgroSup Dijon, INRAE, Univ. Bourgogne, Univ. Bourgogne Franche-Comté, F-21000 Dijon, France

[2] INRAE, UE 0115 DIJ Domaine Expérimental d'Epoisses. Centre de recherche Bourgogne-Franche-Comté, F-21110 Breteniere, France

*** Correspondence:**
anthony.klein@inrae.fr

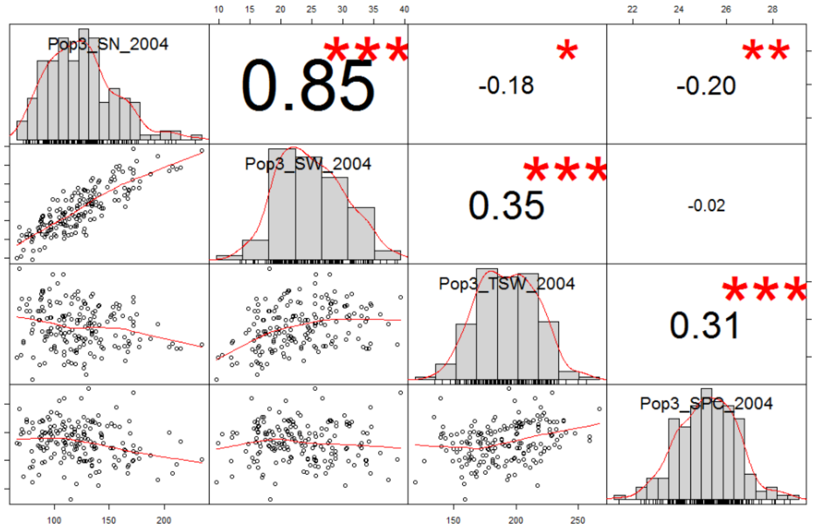
Pop3_SN_2004
0.85
-0.18
-0.20
Pop3_SW_2004
0.35
-0.02
Pop3_TSW_2004
0.31
Pop3_SPC_2004

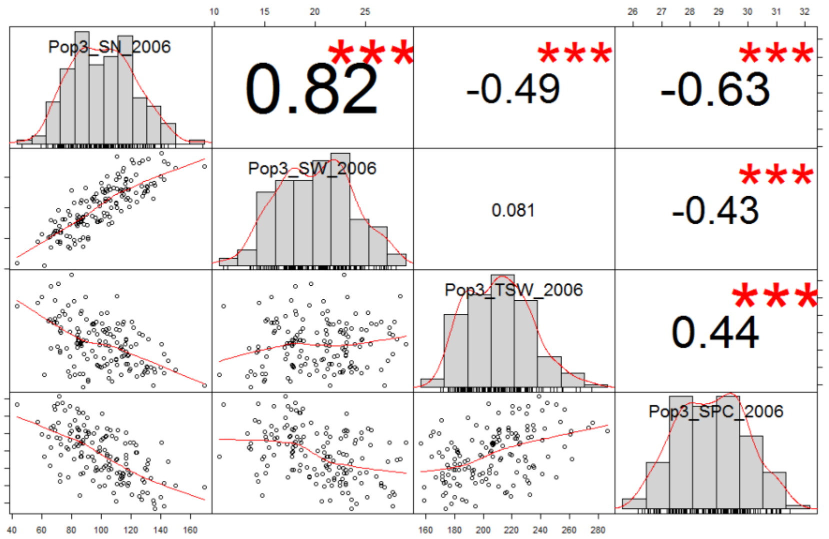
Pop3_SN_2006
0.82
-0.49
-0.63
Pop3_SW_2006
0.081
-0.43
Pop3_TSW_2006
0.44
Pop3_SPC_2006

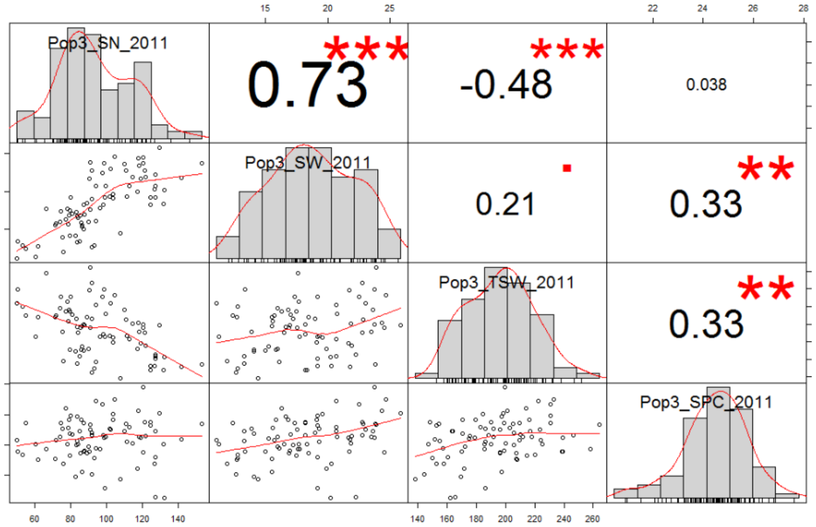
Pop3_SN_2011
0.73
-0.48
0.038
Pop3_SW_2011
0.21
0.33
Pop3_TSW_2011
0.33
Pop3_SPC_2011

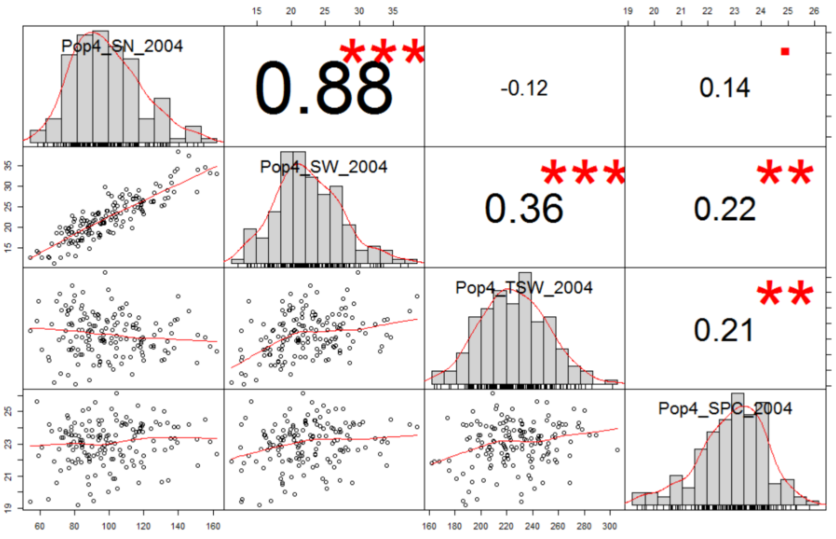
Pop4_SN_2004
0.88
-0.12
0.14
Pop4_SW_2004
0.36
0.22
Pop4_TSW_2004
0.21
Pop4_SPC_2004

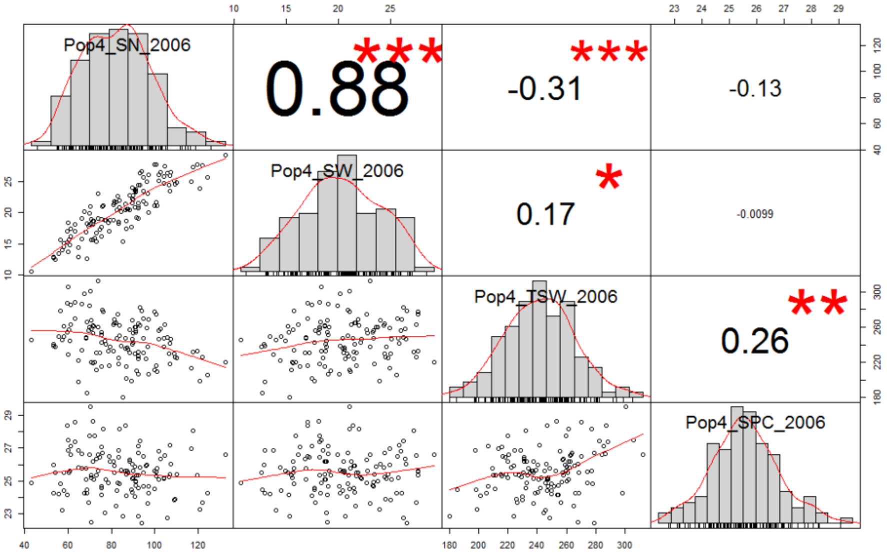
Pop4_SN_2006
0.88
-0.31
-0.13
Pop4_SW_2006
0.17
-0.0099
Pop4_TSW_2006
0.26
Pop4_SPC_2006

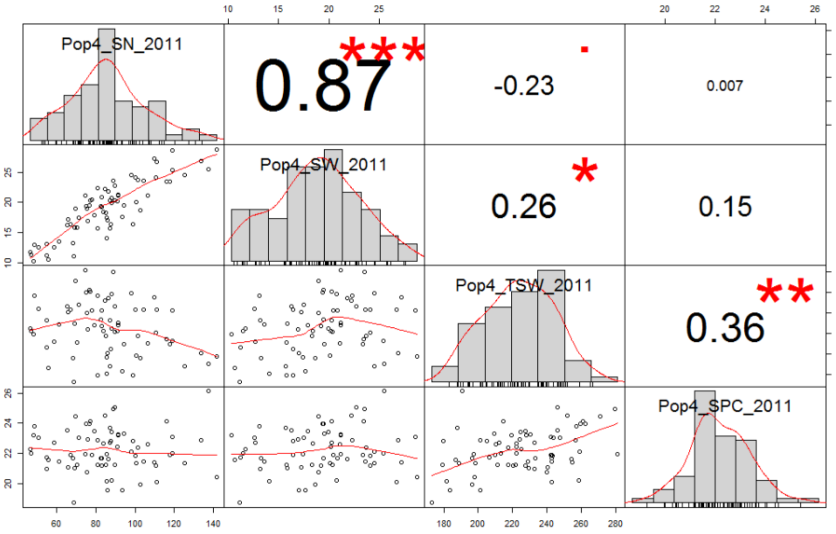
Pop4_SN_2011
0.87
-0.23
0.007
Pop4_SW_2011
0.26
0.15
Pop4_TSW_2011
0.36
Pop4_SPC_2011

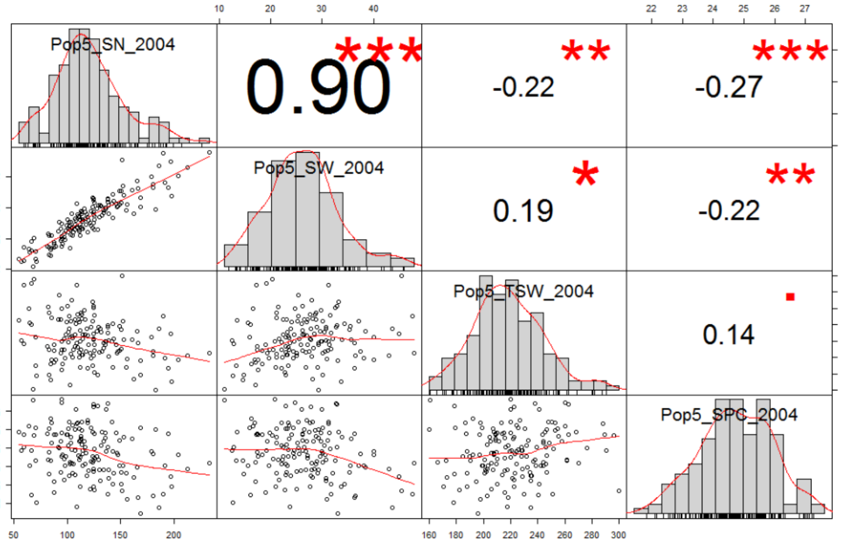
Pop5_SN_2004
Pop5_SW_2004
Pop5_TSW_2004
Pop5_SPC_2004
0.90 ***
-0.22 **
-0.27 ***
0.19 *
-0.22 **
0.14

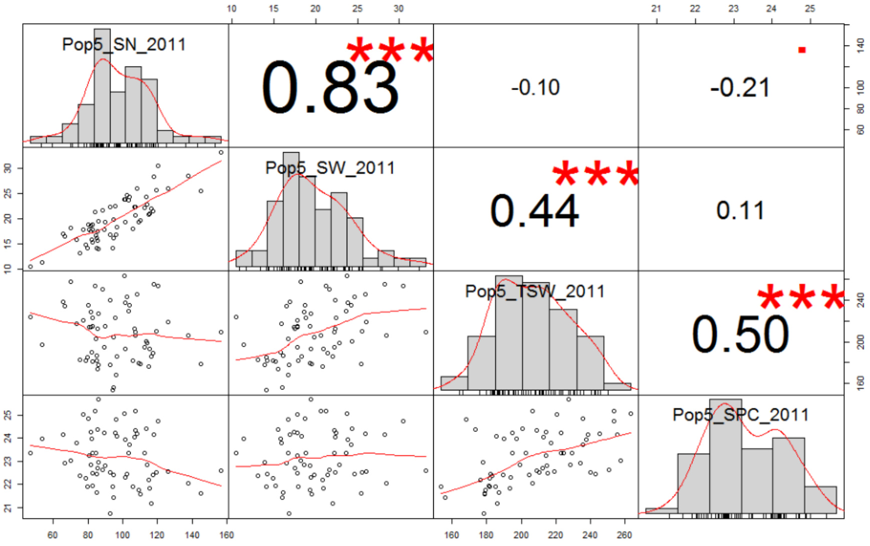
Pop5_SN_2011
Pop5_SW_2011
Pop5_TSW_2011
Pop5_SPC_2011
0.83 ***
-0.10
-0.21
0.44 ***
0.11
0.50 ***

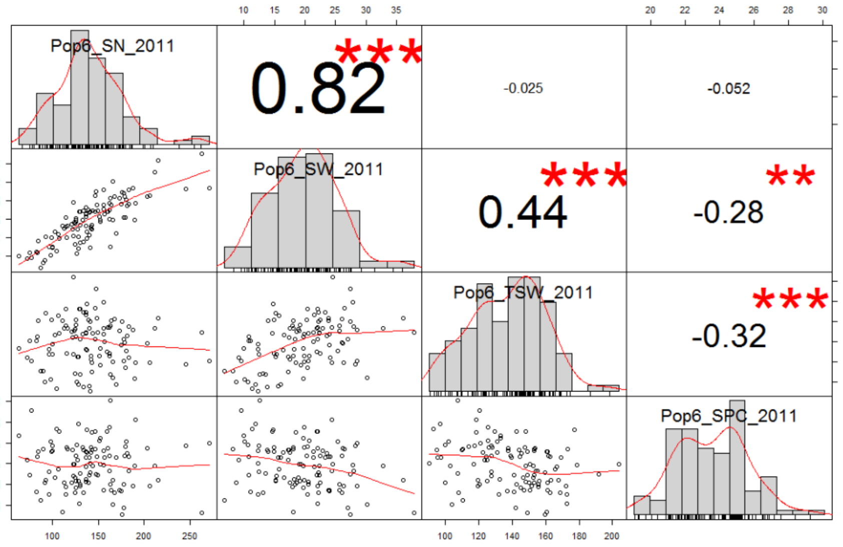
Pop6_SN_2011
Pop6_SW_2011
Pop6_TSW_2011
Pop6_SPC_2011
0.82 ***
-0.025
-0.052
0.44 ***
-0.28 **
-0.32 ***

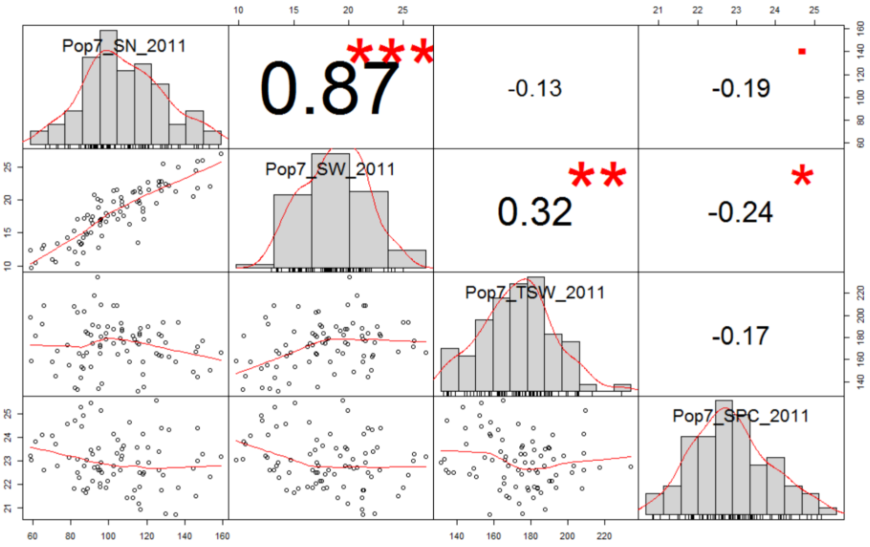
Pop7_SN_2011
Pop7_SW_2011
Pop7_TSW_2011
Pop7_SPC_2011
0.87 ***
-0.13
-0.19
0.32 **
-0.24 *
-0.17

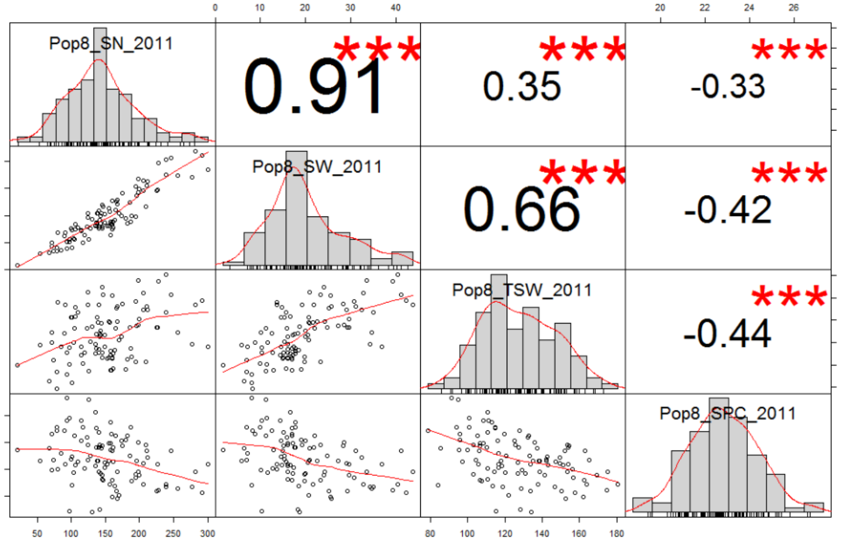
Pop8_SN_2011
Pop8_SW_2011
Pop8_TSW_2011
Pop8_SPC_2011
0.91 ***
0.35 ***
-0.33 ***
0.66 ***
-0.42 ***
-0.44 ***

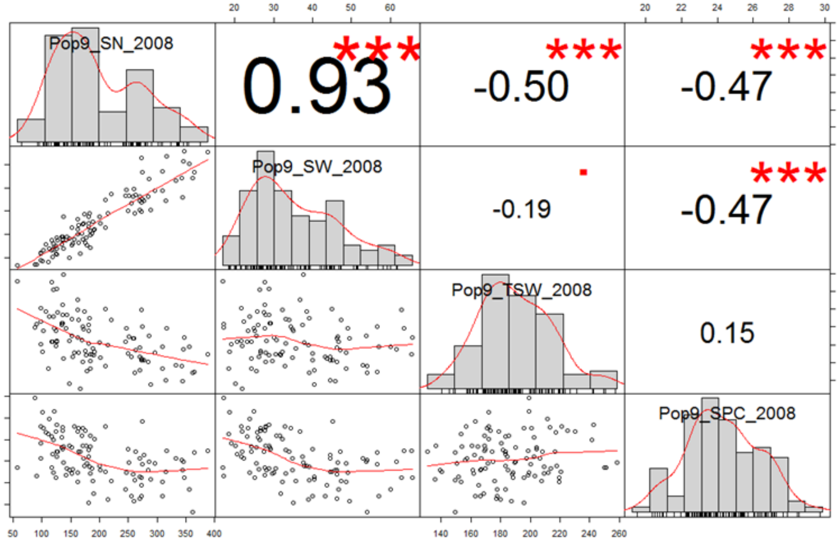
Pop9_SN_2008
Pop9_SW_2008
Pop9_TSW_2008
Pop9_SPC_2008
0.93 ***
-0.50 ***
-0.47 ***
-0.19 .
-0.47 ***
0.15

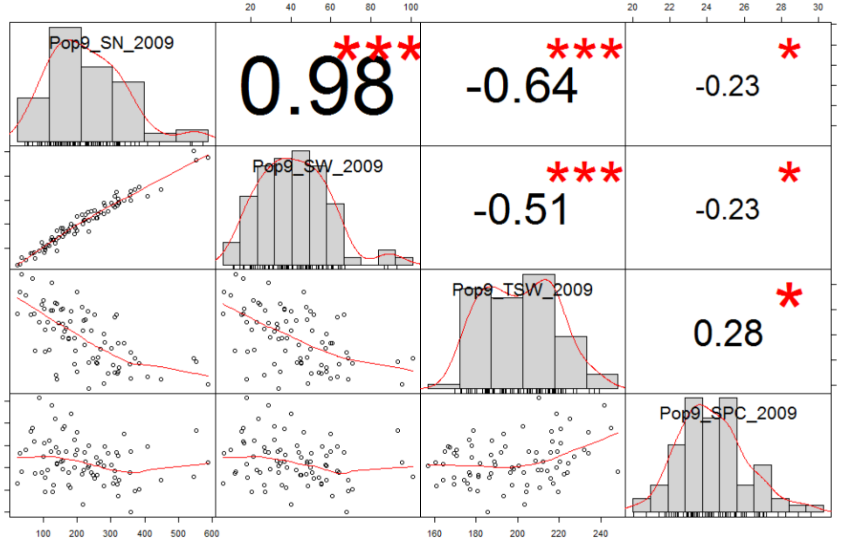
Pop9_SN_2009
Pop9_SW_2009
Pop9_TSW_2009
Pop9_SPC_2009
0.98 ***
-0.64 ***
-0.23 *
-0.51 ***
-0.23 *
0.28 *

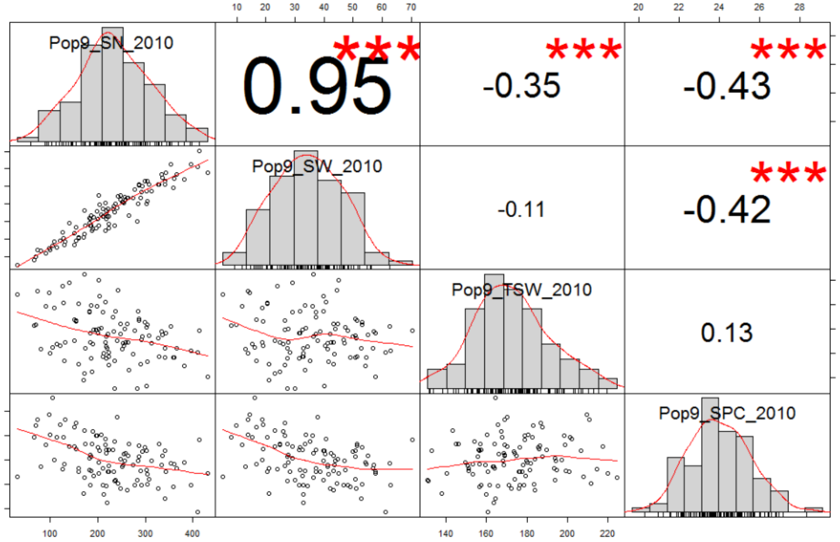
Pop9_SN_2010
Pop9_SW_2010
Pop9_TSW_2010
Pop9_SPC_2010
0.95 ***
-0.35 ***
-0.43 ***
-0.11
-0.42 ***
0.13

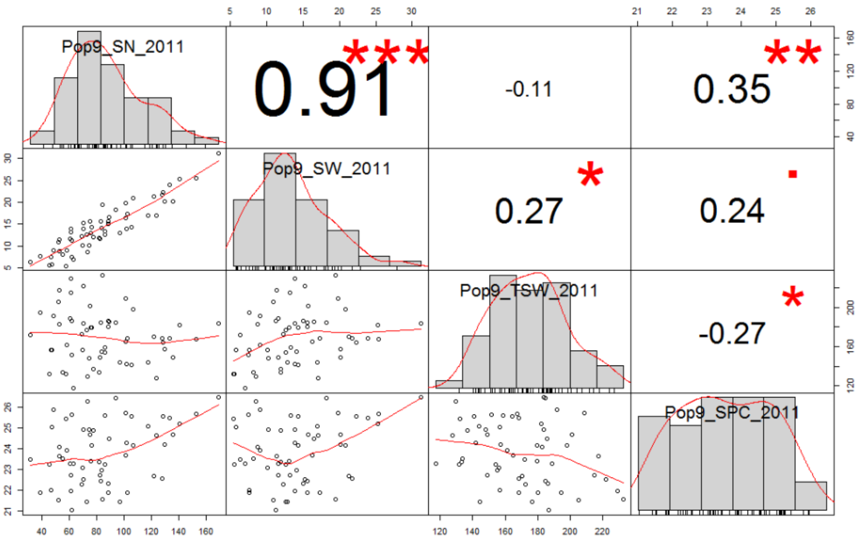
Pop9_SN_2011
0.91
-0.11
0.35
Pop9_SW_2011
0.27
0.24
Pop9_TSW_2011
-0.27
Pop9_SPC_2011

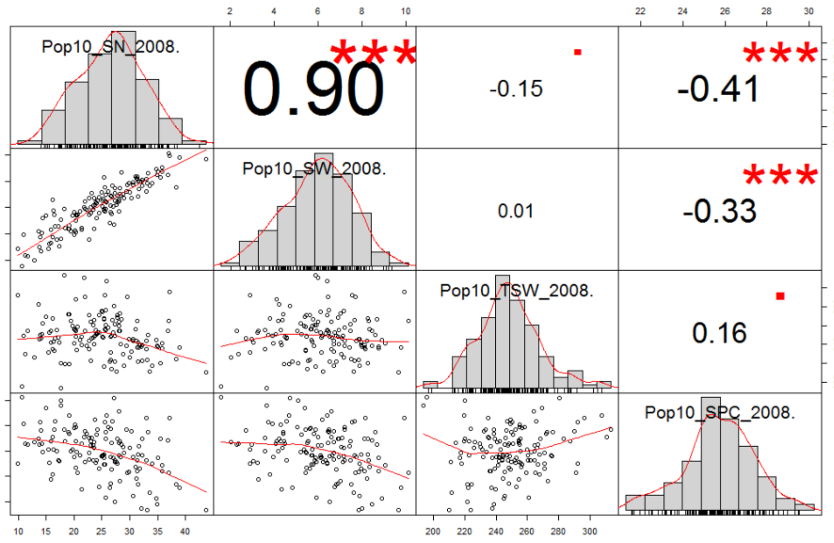
Pop10_SN_2008.
0.90
-0.15
-0.41
Pop10_SW_2008.
0.01
-0.33
Pop10_TSW_2008.
0.16
Pop10_SPC_2008.

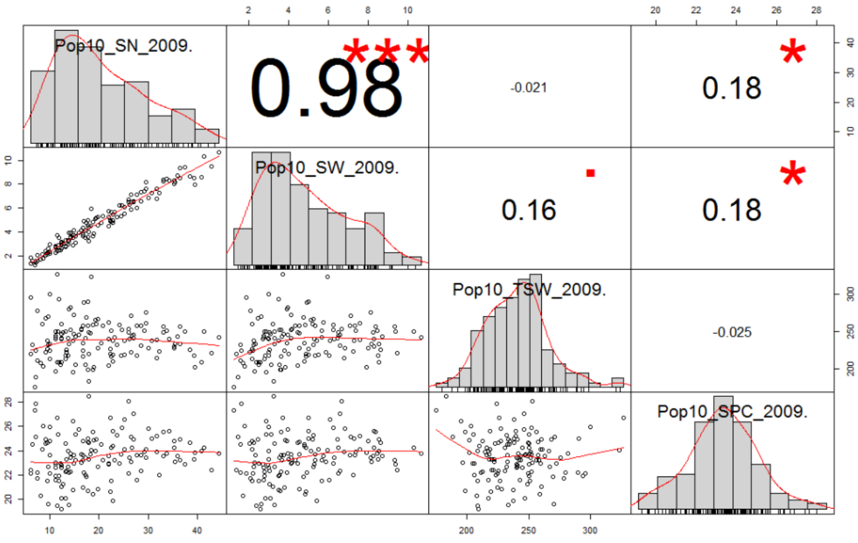
Pop10_SN_2009.
0.98
-0.021
0.18
Pop10_SW_2009.
0.16
0.18
Pop10_TSW_2009.
-0.025
Pop10_SPC_2009.

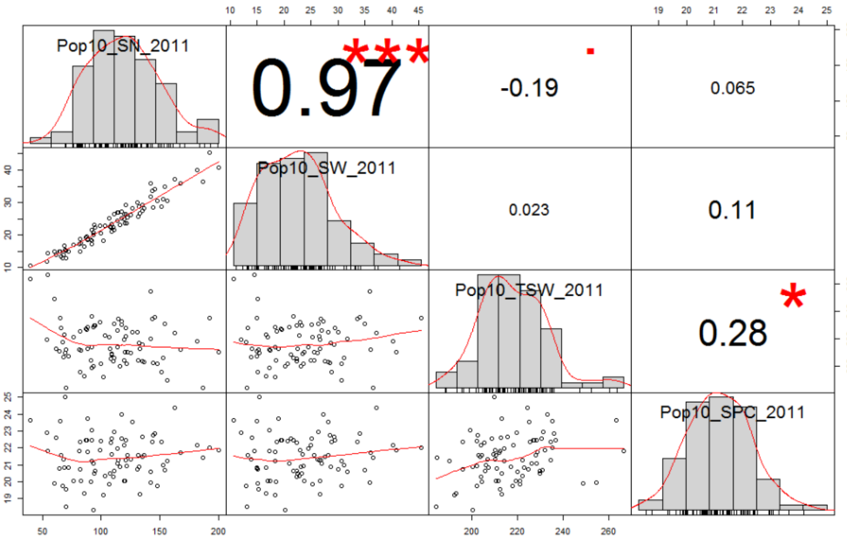
Pop10_SN_2011
0.97
-0.19
0.065
Pop10_SW_2011
0.023
0.11
Pop10_TSW_2011
0.28
Pop10_SPC_2011

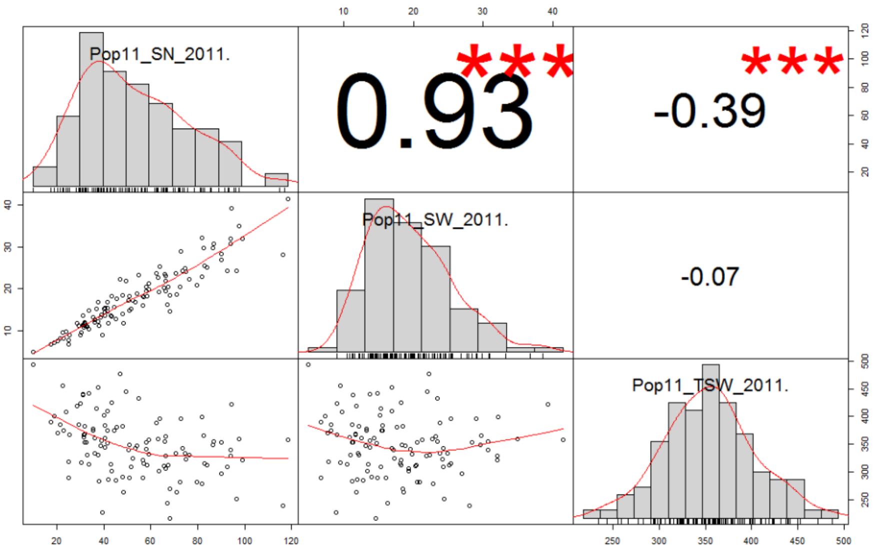
Pop11_SN_2011.
0.93
***
-0.39
***
Pop11_SW_2011.
-0.07
Pop11_TSW_2011.

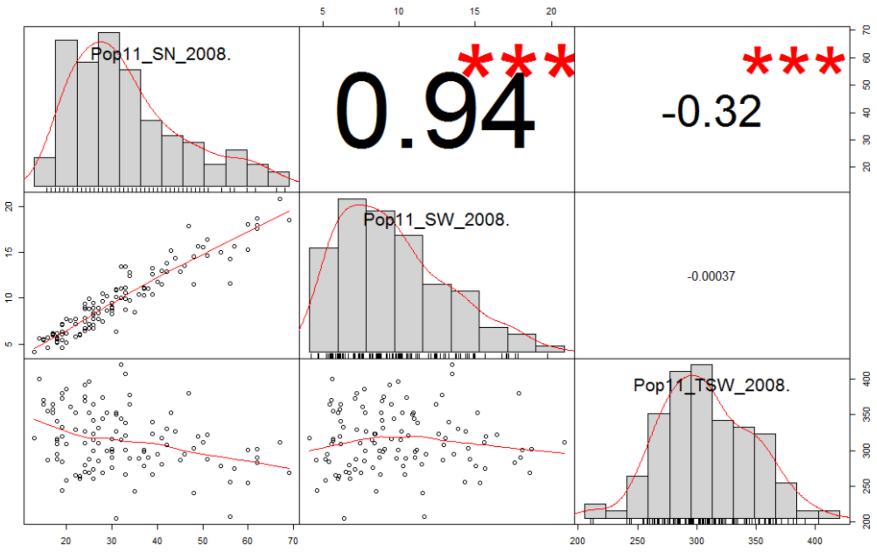
Pop11_SN_2008.
0.94
***
-0.32
***
Pop11_SW_2008.
-0.00037
Pop11_TSW_2008.
