# Supplementary material for: Meta-analysis of QTL reveals the genetic control of yield-related traits and seed protein content in pea
Source: Sci Rep. 2020 Sep 28;10:15925. doi: 10.1038/s41598-020-72548-9 (PMC7522997; doi:10.1038/s41598-020-72548-9)

Meta-analysis of QTL reveals the genetic control of yield-related traits and seed protein content in pea

Anthony Klein[1*], Hervé Houtin[1], Céline Rond-Coissieux[1], Myriam Naudet-Huart[1], Michael Touratier[1], Pascal Marget[2,1] and Judith Burstin[1]

[1] Agroécologie, AgroSup Dijon, INRAE, Univ. Bourgogne, Univ. Bourgogne Franche-Comté, F-21000 Dijon, France

[2] INRAE, UE 0115 DIJ Domaine Expérimental d'Epoisses. Centre de recherche Bourgogne-Franche-Comté, F-21110 Breteniere, France

*** Correspondence:**
anthony.klein@inrae.fr

Foliage type

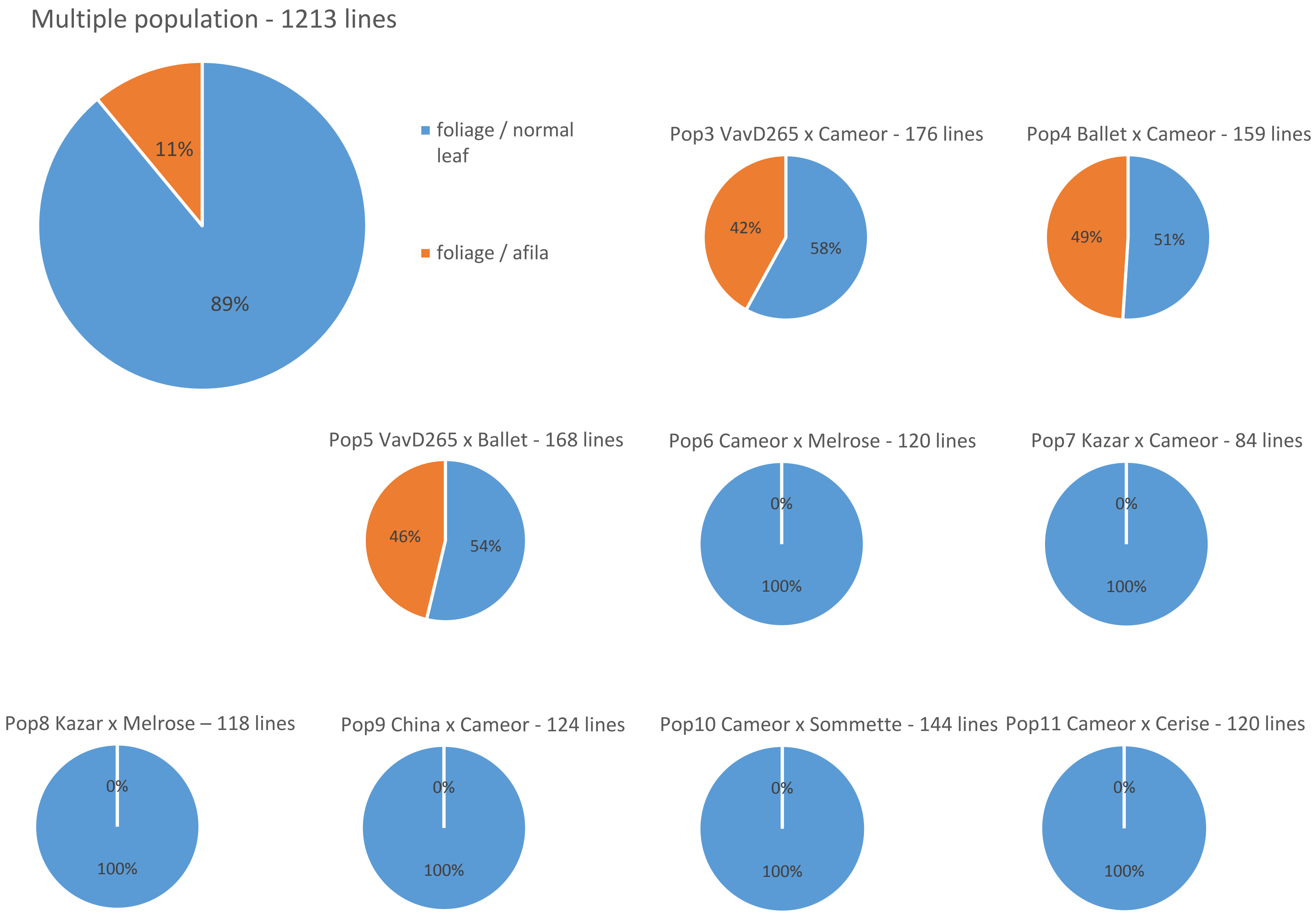

Flower color

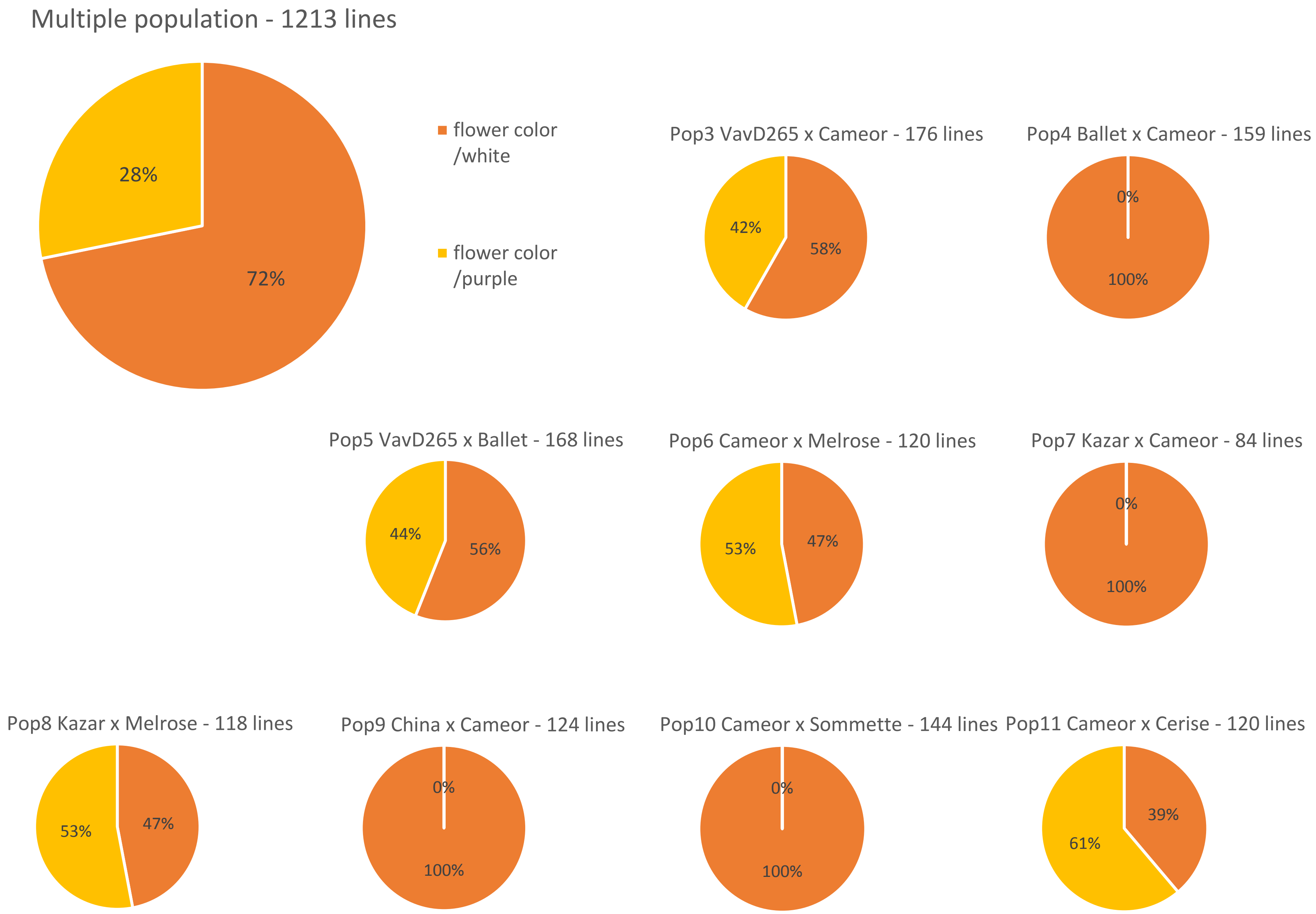

Internode length

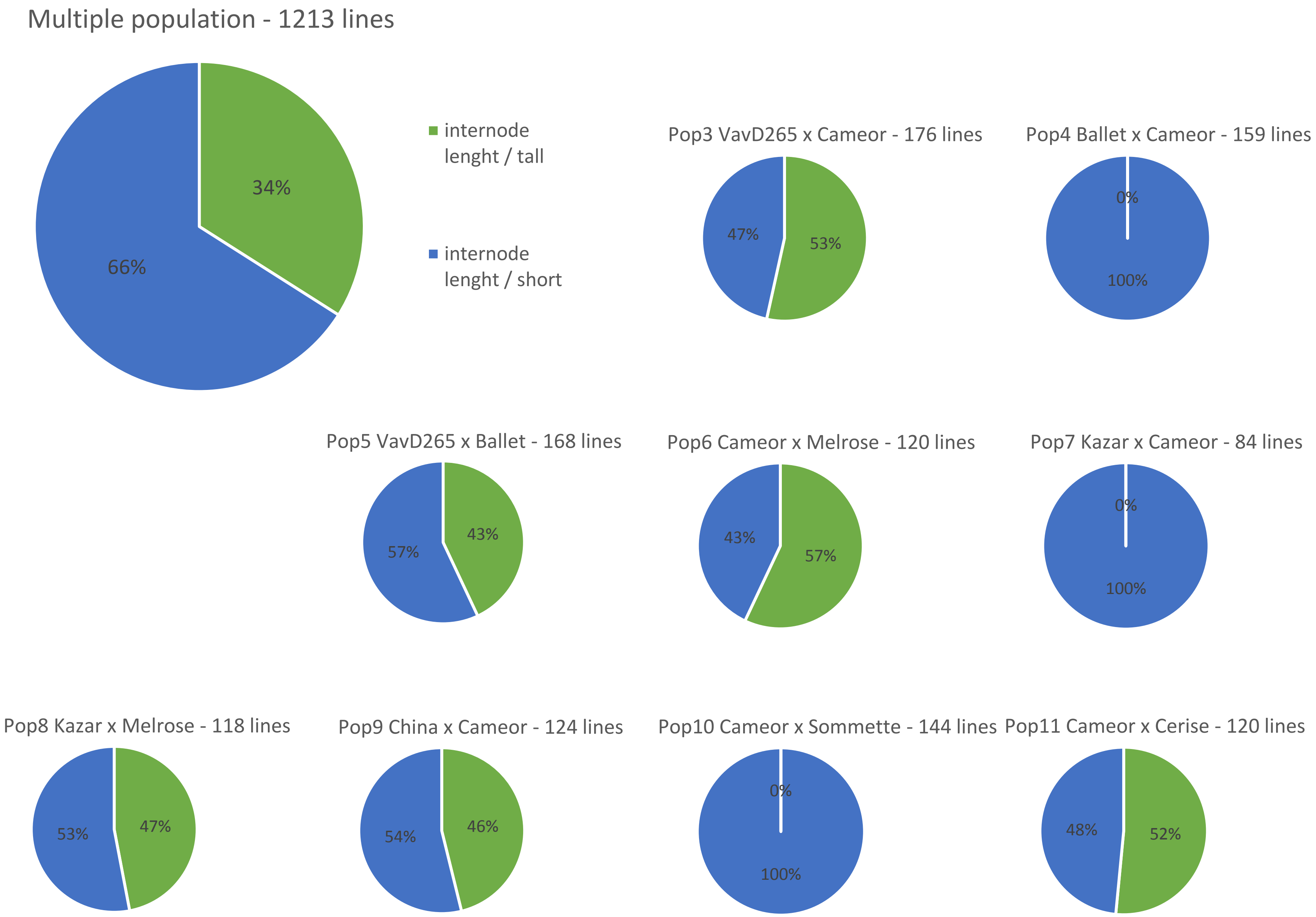

Hilum color

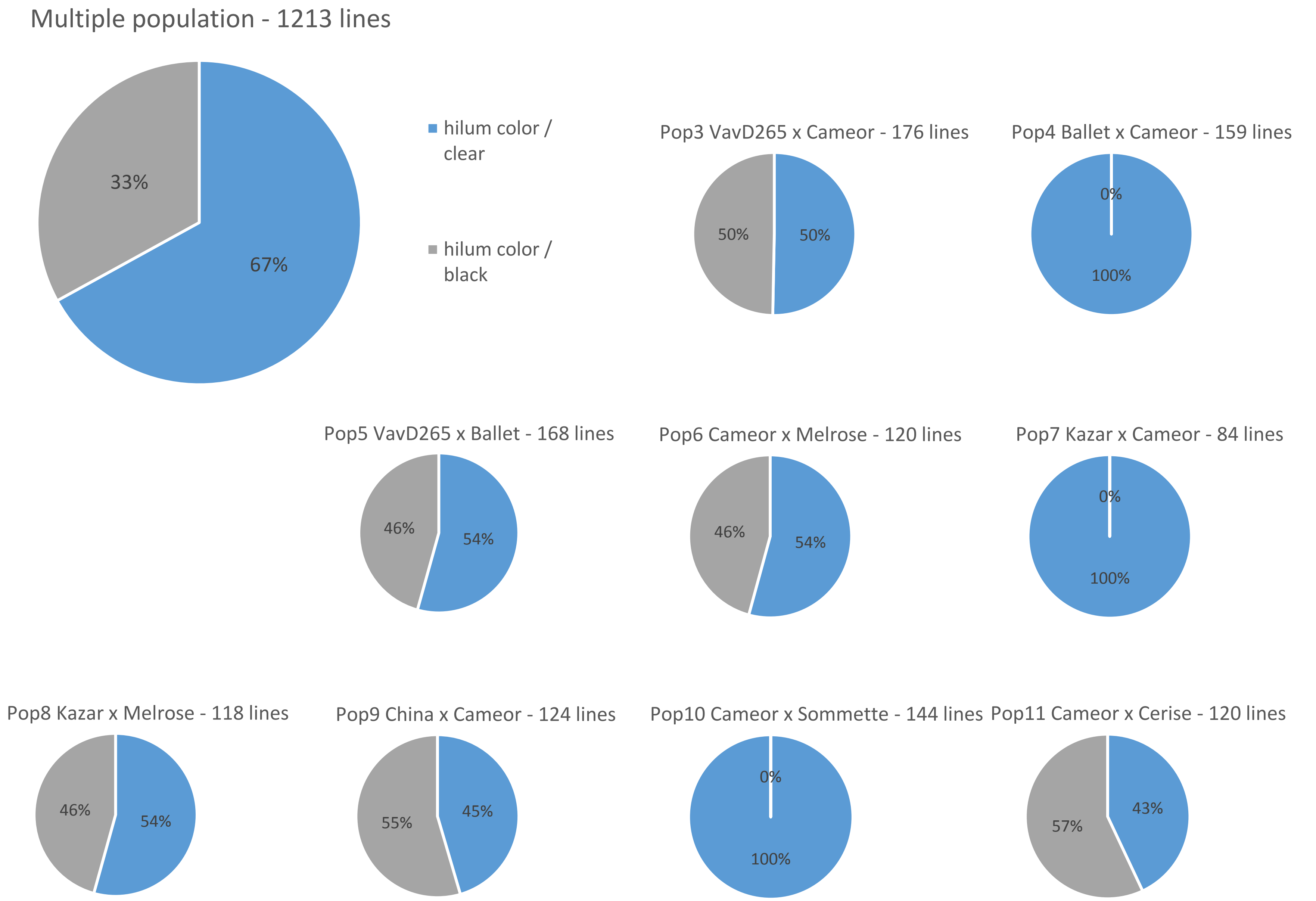

Multiple population - 1213 lines
33%
67%
hilum color / clear
hilum color / black
Pop3 VavD265 x Cameor - 176 lines
50%
50%
Pop4 Ballet x Cameor - 159 lines
0%
100%
Pop5 VavD265 x Ballet - 168 lines
46%
54%
Pop6 Cameor x Melrose - 120 lines
46%
54%
Pop7 Kazar x Cameor - 84 lines
0%
100%
Pop8 Kazar x Melrose - 118 lines
46%
54%
Pop9 China x Cameor - 124 lines
55%
45%
Pop10 Cameor x Sommette - 144 lines
0%
100%
Pop11 Cameor x Cerise - 120 lines
57%
43%

Cotyledon color

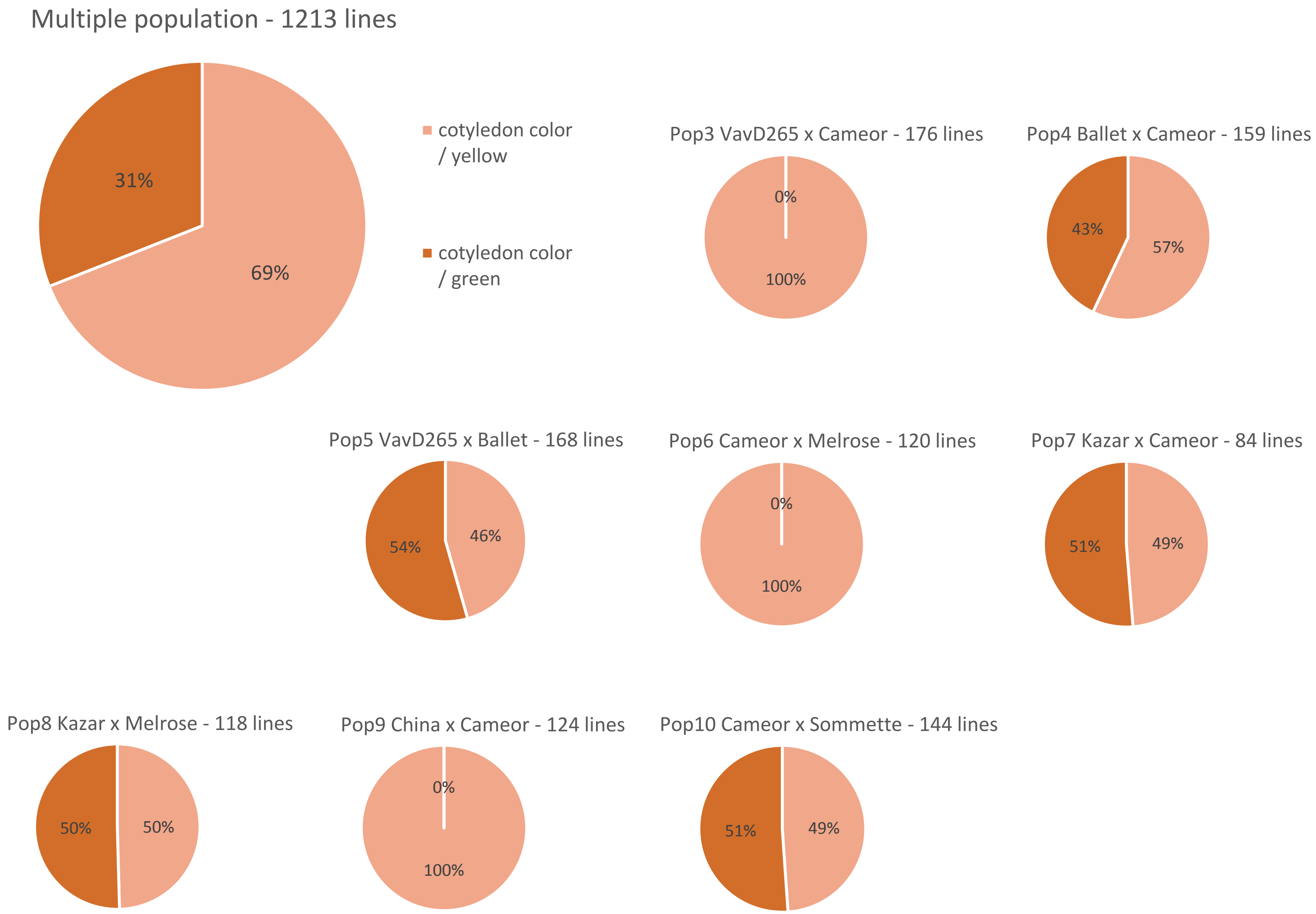

Internode length – multi-population field 2011

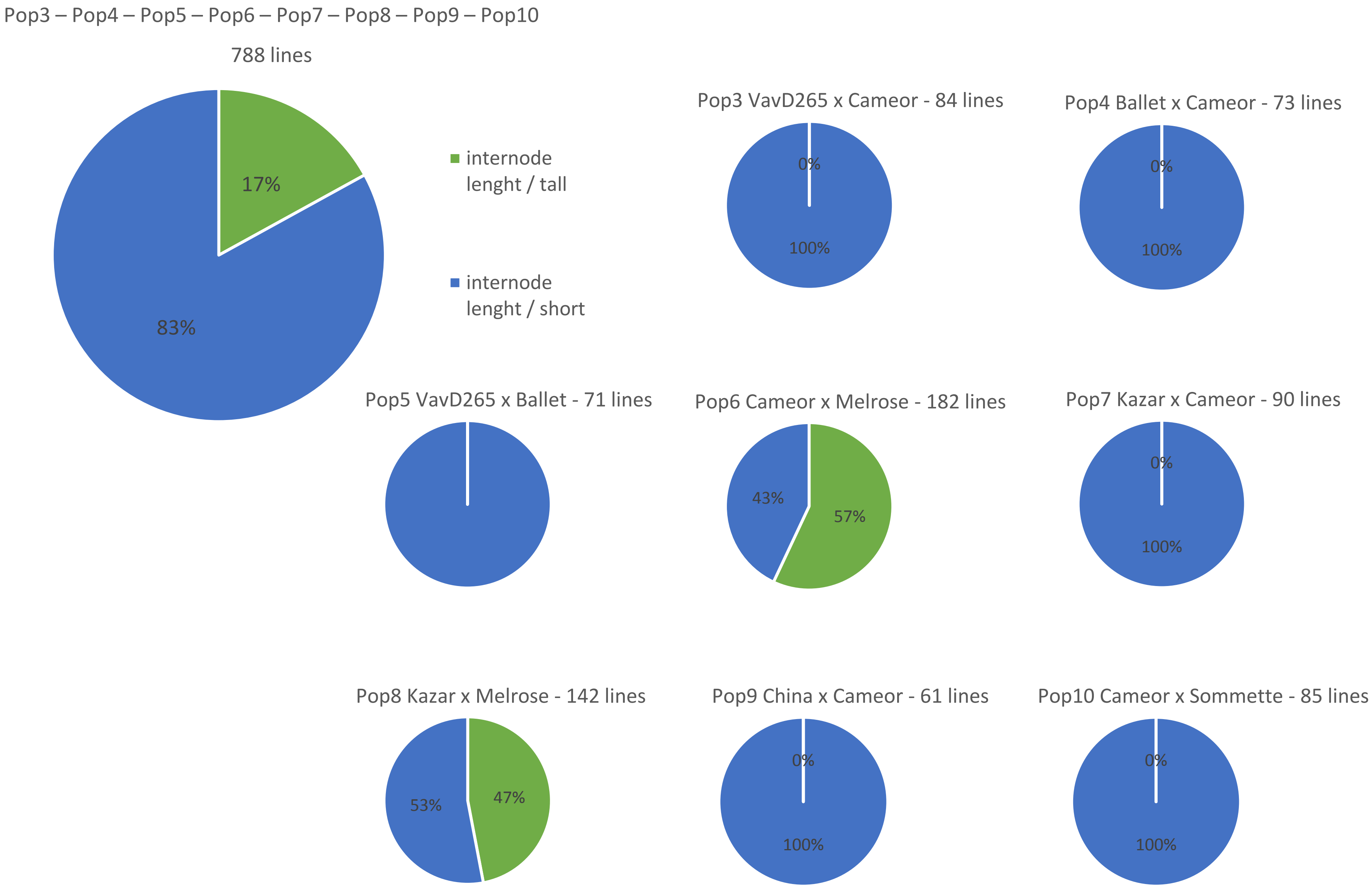

Supplement: Supplementary file 3 — Supplementary Figure 3. [file 41598_2020_72548_MOESM3_ESM.pdf]
