# Supplementary material for: Meta-analysis of QTL reveals the genetic control of yield-related traits and seed protein content in pea
Source: Sci Rep. 2020 Sep 28;10:15925. doi: 10.1038/s41598-020-72548-9 (PMC7522997; doi:10.1038/s41598-020-72548-9)
Supplement: Supplementary file 4 — Supplementary Table 1. [file 41598_2020_72548_MOESM4_ESM.pdf]

Meta-analysis of QTL reveals the genetic control of yield-related traits and seed protein content in pea

Anthony Klein[1*], Hervé Houtin[1], Céline Rond-Coissieux[1], Myriam Naudet-Huart[1], Michael Touratier[1], Pascal Marget[2,1] and Judith Burstin[1]

[1] Agroécologie, AgroSup Dijon, INRAE, Univ. Bourgogne, Univ. Bourgogne Franche-Comté, F-21000 Dijon, France

[2] INRAE, UE 0115 DIJ Domaine Expérimental d'Epoisses. Centre de recherche Bourgogne-Franche-Comté, F-21110 Breteniere, France

*** Correspondence:**
anthony.klein@inrae.fr

Table S1 : Mean parental and recombinant inbred values of Pop3 to Pop11, standard deviation, heritabilities and significance of genotype effect measured between 2004 and 2011 at INRAE Dijon

Population name	Trial [a]	Trait [b]	Caméor	VavD265	Mean Pop3	StdDev	Minimum	Maximum	h^2 [c]	Probability [d]
Pop3	2004	SN	94.3	130.7	122.7	32.6	66.4	233.7	0.56	0.01
Pop3	2006	SN	85.3	126.1	99.6	22.2	43.2	169.1	0.80	< 0.0001
Pop3	2011	SN	89.3	133.8	98.8	26.9	50.1	196.2	0.82	< 0.0001
Pop3	2004	SPC	25.0	25.9	25.3	1.4	21.2	29.1	0.41	0.04
Pop3	2006	SPC	28.4	29.9	28.8	1.5	25.6	32.2	0.93	< 0.0001
Pop3	2011	SPC	24.3	23.8	24.3	1.4	20.5	27.8	0.89	< 0.0001
Pop3	2004	SW	19.7	23.8	23.1	6.2	9.6	39.4	0.47	0.04
Pop3	2006	SW	18.3	23.9	20.3	3.9	10.5	29.0	0.71	< 0.0001
Pop3	2011	SW	19.2	23.8	19.0	5.1	11.1	39.8	0.84	< 0.0001
Pop3	2004	TSW	209.0	182.0	189.6	28.7	119.0	267.0	0.92	< 0.0001
Pop3	2006	TSW	212.0	190.0	206.7	27.1	156.7	286.6	0.94	< 0.0001
Pop3	2011	TSW	215.0	174.7	194.0	28.1	138.2	264.4	0.98	< 0.0001

Population name	Trial [a]	Trait [b]	Caméor	Ballet	Mean Pop4	StdDev	Minimum	Maximum	h^2 [c]	Probability
Pop4	2004	SN	94.3	73.1	99.6	22.4	54.4	161.9	0.60	0.007
Pop4	2006	SN	85.3	76.6	83.8	17.5	43.1	132.5	0.71	< 0.0001
Pop4	2011	SN	89.3	72.8	85.5	21.6	46.5	141.6	0.81	< 0.0001
Pop4	2004	SPC	25.0	22.1	23.0	1.4	19.2	26.2	0.64	0.003
Pop4	2006	SPC	28.4	23.7	25.6	1.4	22.4	29.5	0.81	< 0.0001
Pop4	2011	SPC	24.2	20.1	22.2	1.4	18.7	26.1	0.90	< 0.0001
Pop4	2004	SW	19.7	18.5	22.3	5.5	8.3	38.5	0.63	0.004
Pop4	2006	SW	18.3	20.1	20.4	4.4	10.6	29.2	0.64	< 0.0001
Pop4	2011	SW	19.2	16.7	19.1	4.6	10.3	28.8	0.82	< 0.0001
Pop4	2004	TSW	209.0	251.0	226.7	27.4	162.0	305.9	0.92	< 0.0001
Pop4	2006	TSW	212.0	262.0	245.6	25.2	180.0	312.6	0.81	< 0.0001
Pop4	2011	TSW	215.0	227.3	223.9	27.5	144.5	281.3	0.98	< 0.0001

Population name	Trial [a]	Trait [b]	Ballet	VavD265	Mean Pop5	StdDev	Minimum	Maximum	h^2 [c]	Probability
Pop5	2004	SN	73.1	130.7	120.1	33.8	54.6	232.9	0.65	0.003
Pop5	2011	SN	72.8	133.8	96.2	21.9	46.8	165.8	0.82	< 0.0001
Pop5	2004	SPC	22.1	25.9	24.7	1.3	21.4	27.6	0.72	0.0005
Pop5	2011	SPC	20.1	23.8	23.2	1.1	20.7	25.7	0.88	< 0.0001
Pop5	2004	SW	18.5	23.8	26.5	7.3	10.9	47.9	0.58	0.01
Pop5	2011	SW	16.7	23.8	19.8	5.2	10.1	38.1	0.85	< 0.0001
Pop5	2004	TSW	251.0	182.0	222.0	27.2	160.0	300.1	0.85	< 0.0001
Pop5	2011	TSW	227.3	174.7	207.4	27.3	135.9	263.3	0.98	< 0.0001

Population name	Trial [a]	Trait [b]	Caméor	Melrose	Mean Pop6	StdDev	Minimum	Maximum	h^2 [c]	Probability
Pop6	2011	SN	89.3	141.1	137.0	42.5	12.7	270.7	0.92	< 0.0001
Pop6	2011	SPC	24.3	23.2	24.1	2.3	19.0	30.1	0.93	< 0.0001
Pop6	2011	SW	19.2	13.7	19.5	6.1	6.7	37.9	0.90	< 0.0001
Pop6	2011	TSW	215.0	99.7	138.5	23.4	90.6	204.0	0.98	< 0.0001

Population name	Trial [a]	Trait [b]	Caméor	Kazar	Mean Pop7	StdDev	Minimum	Maximum	h^2 [c]	Probability
Pop7	2011	SN	89.3	125.2	104.0	23.0	58.7	159.0	0.82	< 0.0001
Pop7	2011	SPC	24.3	21.6	22.9	1.1	20.7	25.6	0.85	< 0.0001
Pop7	2011	SW	19.2	20.3	18.0	4.5	6.3	30.8	0.84	< 0.0001
Pop7	2011	TSW	215.0	165.6	173.9	21.0	131.4	234.5	0.98	< 0.0001

Population name	Trial [a]	Trait [b]	Kazar	Melrose	Mean Pop8	StdDev	Minimum	Maximum	h^2 [c]	Probability
Pop8	2011	SN	125.2	141.1	147.5	58.1	9.9	300.0	0.95	< 0.0001
Pop8	2011	SPC	21.6	23.2	23.0	1.9	18.8	29.0	0.91	< 0.0001
Pop8	2011	SW	20.3	13.7	19.4	8.9	1.7	43.7	0.94	< 0.0001
Pop8	2011	TSW	165.6	99.7	127.2	22.8	78.6	180.8	0.99	< 0.0001

Population name	Trial [a]	Trait [b]	Caméor	China	Mean Pop9	StdDev	Minimum	Maximum	h^2 [c]	Probability
Pop9	2008	SN	111.2	232.2	202.1	78.7	57.7	391.3	0.98	< 0.0001

Pop9	2009	SN	203.9	159.2	225.1	124.4	23.8	586.4	0.97	< 0.0001
Pop9	2010	SN	102.9	193.1	233.1	95.4	31.3	585.4	0.75	< 0.0001
Pop9	2011	SN	89.3	79.8	83.2	31.4	11.3	168.7	0.88	< 0.0001
Pop9	2008	SPC	24.4	24.7	24.3	2.1	19.3	29.9	0.76	< 0.0001
Pop9	2009	SPC	26.0	26.5	24.6	2.0	20.0	30.3	0.71	< 0.0001
Pop9	2010	SPC	24.5	23.3	24.0	1.8	19.7	29.1	0.78	< 0.0001
Pop9	2011	SPC	24.3	25.9	23.8	1.5	21.0	26.5	0.91	< 0.0001
Pop9	2008	SW	24.8	43.4	35.7	12.3	16.9	65.6	0.98	< 0.0001
Pop9	2009	SW	47.1	34.1	44.0	24.0	6.0	119.1	0.99	< 0.0001
Pop9	2010	SW	23.4	33.3	37.2	17.5	5.1	102.5	0.75	< 0.0001
Pop9	2011	SW	19.2	14.5	14.1	5.5	5.4	31.2	0.91	< 0.0001
Pop9	2008	TSW	205.6	177.4	185.2	26.2	130.8	258.7	0.97	< 0.0001
Pop9	2009	TSW	229.4	202.2	193.6	26.5	101.4	248.2	0.87	< 0.0001
Pop9	2010	TSW	217.9	172.2	174.1	20.4	122.3	224.6	0.76	< 0.0001
Pop9	2011	TSW	215.0	176.9	167.5	29.0	96.1	232.7	0.99	< 0.0001

Population name	Trial [a]	Trait [b]	Caméor	Sommette	Mean Pop10	StdDev	Minimum	Maximum	h^2 [c]	Probability
Pop10	2008*	SN	*na*	*na*	24.4	7.1	8.0	43.6	0.84	< 0.0001
Pop10	2009*	SN	19.3	13.1	19.9	9.3	6.3	44.3	0.90	< 0.0001
Pop10	2011	SN	89.3	38.2	108.7	34.8	36.5	199.7	0.90	< 0.0001
Pop10	2008*	SPC	*na*	*na*	26.0	1.9	21.3	30.2	0.77	< 0.0001
Pop10	2009*	SPC	*na*	*na*	23.5	1.9	19.1	28.5	0.71	< 0.0001
Pop10	2011	SPC	24.3	19.9	21.5	1.4	18.3	25.0	0.89	< 0.0001
Pop10	2008*	SW	*na*	*na*	6.1	1.9	1.0	10.1	0.87	< 0.0001
Pop10	2009*	SW	4.4	3.4	4.8	2.3	1.2	10.7	0.84	< 0.0001
Pop10	2011	SW	19.2	9.2	23.5	7.5	10.6	45.4	0.92	< 0.0001
Pop10	2008*	TSW	*na*	*na*	248.5	21.7	193.6	313.4	0.91	< 0.0001
Pop10	2009*	TSW	225.7	251.2	239.0	26.1	175.1	327.1	0.84	< 0.0001
Pop10	2011	TSW	215.0	231.8	218.0	19.1	184.3	317.3	0.97	< 0.0001

Population name	Trial [a]	Trait [b]	Caméor	Cerise	Mean Pop11	StdDev	Minimum	Maximum	h^2 [c]	Probability
Pop11	2008*	SN	*na*	18.0	33.2	15.8	13.0	118.0	0.26	0.16

Pop11	2011*	SN	39.3	35.3	53.8	23.2	10.0	118.5	0.53	< 0.0001
Pop11	2008*	SW	*na*	8.2	10.2	4.4	4.1	30.7	0.21	0.22
Pop11	2011*	SW	8.5	18.1	17.9	7.3	5.0	41.4	0.59	< 0.0001
Pop11	2008*	TSW	*na*	419.6	314.0	42.5	205.5	420.3	0.31	0.13
Pop11	2011*	TSW	217.0	492.2	347.4	54.9	202.6	493.5	0.78	< 0.0001

[a] phenotyping in the fields environments at INRAE Dijon, Domaine d'Epoisses, * phenotyping in glasshouse at INRAE Dijon

[b] *SN* seed number per plant, *SPC* seed protein content (% of seed dry weight), *SW* seed weight per plant (g), *TSW* thousand seed weight (g)

[c] heritability [$h^2 = 1-(1/F)$] (F : value from Fisher test)

[d] Significance of genotype effect, *P*-value from ANOVA test.

na: missing value
