# Supplementary material for: Meta-analysis of QTL reveals the genetic control of yield-related traits and seed protein content in pea
Source: Sci Rep. 2020 Sep 28;10:15925. doi: 10.1038/s41598-020-72548-9 (PMC7522997; doi:10.1038/s41598-020-72548-9)
Supplement: Supplementary file 5 — Supplementary Table 2. [file 41598_2020_72548_MOESM5_ESM.pdf]

Meta-analysis of QTL reveals the genetic control of yield-related traits and seed protein content in pea

Anthony Klein[1*], Hervé Houtin[1], Céline Rond-Coissieux[1], Myriam Naudet-Huart[1], Michael Touratier[1], Pascal Marget[2,1] and Judith Burstin[1]

[1] Agroécologie, AgroSup Dijon, INRAE, Univ. Bourgogne, Univ. Bourgogne Franche-Comté, F-21000 Dijon, France

[2] INRAE, UE 0115 DIJ Domaine Expérimental d'Epoisses. Centre de recherche Bourgogne-Franche-Comté, F-21110 Breteniere, France

*** Correspondence:**
anthony.klein@inrae.fr

Table S2 : Genotype, environment and genotype-by-environment interaction effects on the seed number per plant (SN), seed weight per plant (SW), thousand seed weight (TSW) and seed protein content (SPC) measured in field trials at INRAE Dijon.

Population name	Environment	Trait	*P-value*	
Pop3	2004/2006/2011	SN	Population	<0.0001
			Environment	<0.0001
			Population x Environment	0.003
Pop3	2004/2006/2011	SW	Population	<0.0001
			Environment	<0.0001
			Population x Environment	0.001
Pop3	2004/2006/2011	TSW	Population	<0.0001
			Environment	<0.0001
			Population x Environment	<0.0001
Pop3	2004/2006/2011	SPC	Population	<0.0001
			Environment	<0.0001
			Population x Environment	<0.0001
Pop4	2004/2006/2011	SN	Population	<0.0001
			Environment	<0.0001
			Population x Environment	0.1
Pop4	2004/2006/2011	SW	Population	<0.0001
			Environment	<0.0001
			Population x Environment	0.3
Pop4	2004/2006/2011	TSW	Population	<0.0001
			Environment	<0.0001
			Population x Environment	0.4
Pop4	2004/2006/2011	SPC	Population	<0.0001
			Environment	<0.0001
			Population x Environment	0.3
Pop5	2004/2011	SN	Population	<0.0001
			Environment	<0.0001
			Population x Environment	0.005
Pop5	2004/2011	SW	Population	<0.0001
			Environment	<0.0001
			Population x Environment	0.003
Pop5	2004/2011	TSW	Population	<0.0001
			Environment	<0.0001
			Population x Environment	<0.0001
Pop5	2004/2011	SPC	Population	<0.0001
			Environment	<0.0001
			Population x Environment	<0.0001
Pop9	2008/2009/2010/2011	SN	Population	<0.0001
			Environment	<0.0001
			Population x Environment	<0.0001
Pop9	2008/2009/2010/2011	SW	Population	<0.0001
			Environment	<0.0001

			Population x Environment	<0.0001
Pop9	2008/2009/2010/2011	TSW	Population	<0.0001
			Environment	<0.0001
			Population x Environment	<0.0001
Pop9	2008/2009/2010/2011	SPC	Population	<0.0001
			Environment	0.1
			Population x Environment	<0.0001
