# Supplementary material for: Meta-analysis of QTL reveals the genetic control of yield-related traits and seed protein content in pea
Source: Sci Rep. 2020 Sep 28;10:15925. doi: 10.1038/s41598-020-72548-9 (PMC7522997; doi:10.1038/s41598-020-72548-9)
Supplement: Supplementary file 6 — Supplementary Table 3. [file 41598_2020_72548_MOESM6_ESM.pdf]

Meta-analysis of QTL reveals the genetic control of yield-related traits and seed protein content in pea

Anthony Klein[1*], Hervé Houtin[1], Céline Rond-Coissieux[1], Myriam Naudet-Huart[1], Michael Touratier[1], Pascal Marget[2,1] and Judith Burstin[1]

[1] Agroécologie, AgroSup Dijon, INRAE, Univ. Bourgogne, Univ. Bourgogne Franche-Comté, F-21000 Dijon, France

[2] INRAE, UE 0115 DIJ Domaine Expérimental d'Epoisses. Centre de recherche Bourgogne-Franche-Comté, F-21110 Breteniere, France

*** Correspondence:**
anthony.klein@inrae.fr

Table S3 : QTL parameters detected in multi-population Pop3 to Pop11 from 2004 to 2011 at INRAE Dijon

Linkage group	Chromosome	QTL position (cM) [a]	QTL identification [b]	Pvalue [c]	R^2 [d]	Confidence interval (cM) [e]			Allelic value [f]															
						max position	min	max	VavD265		Cameor		Ballet		Melrose		Kazar		China		Sommette		Cerise	
LG1	Chr2	0.0	SPC06	5.38	0.09	0.00	0.00	4.61	VavD265_multicrossVavD265CameorBallet	0.28	Cameor_multicrossVavD265CameorBallet	-0.16	Ballet_multicrossVavD265CameorBallet	-0.13	-	-	-	-	-	-	-	-	-	-
LG1	Chr2	2.3	TSW11	18.05	0.13	2.30	0.62	3.59	VavD265_multicrossVavD265CameorBalletKazarChinaSommetteCeriseMelrose	11.99	Cameor_multicrossVavD265CameorBalletKazarChinaSommetteCeriseMelrose	-4.73	Ballet_multicrossVavD265CameorBalletKazarChinaSommetteCeriseMelrose	-6.99	Melrose_multicrossVavD265CameorBalletKazarChinaSommetteCeriseMelrose	-7.27	Kazar_multicrossVavD265CameorBalletKazarChinaSommetteCeriseMelrose	-4.65	China_multicrossVavD265CameorBalletKazarChinaSommetteCeriseMelrose	-9.39	Sommette_multicrossVavD265CameorBalletKazarChinaSommetteCeriseMelrose	-0.43	Cerise_multicrossVavD265CameorBalletKazarChinaSommetteCeriseMelrose	21.47
LG1	Chr2	2.8	TSW04	38.44	0.32	2.30	1.27	3.24	VavD265_multicrossVavD265CameorBallet	12.21	Cameor_multicrossVavD265CameorBallet	-5.16	Ballet_multicrossVavD265CameorBallet	-7.05	-	-	-	-	-	-	-	-	-	-
LG1	Chr2	2.9	TSW06	20.02	0.29	2.90	1.78	4.06	VavD265_multicrossVavD265CameorBallet	12.62	Cameor_multicrossVavD265CameorBallet	-6.13	Ballet_multicrossVavD265CameorBallet	-6.49	-	-	-	-	-	-	-	-	-	-
LG1	Chr2	4.4	SPC04	8.72	0.08	4.40	0.00	6.56	VavD265_multicrossVavD265CameorBallet	0.30	Cameor_multicrossVavD265CameorBallet	-0.16	Ballet_multicrossVavD265CameorBallet	-0.14	-	-	-	-	-	-	-	-	-	-
LG1	Chr2	4.9	SN04	9.58	0.09	4.90	0.00	7.05	VavD265_multicrossVavD265CameorBallet	-6.46	Cameor_multicrossVavD265CameorBallet	2.73	Ballet_multicrossVavD265CameorBallet	3.73	-	-	-	-	-	-	-	-	-	-
LG1	Chr2	15.2	TSW06	9.57	0.15	15.20	12.94	17.54	VavD265_multicrossVavD265CameorBallet	-4.30	Cameor_multicrossVavD265CameorBallet	-4.59	Ballet_multicrossVavD265CameorBallet	8.89	-	-	-	-	-	-	-	-	-	-
LG1	Chr2	25.5	TSW08	5.77	0.08	25.50	24.06	29.63	-	-	Cameor_multicrossCameorChinaSommetteCerise	-4.90	-	-	-	-	-	-	China_multicrossCameorChinaSommetteCerise	8.16	Sommette_multicrossCameorChinaSommetteCerise	-2.55	Cerise_multicrossCameorChinaSommetteCerise	-0.71
LG1	Chr2	26.1	TSW04	4.85	0.05	26.10	11.54	32.80	VavD265_multicrossVavD265CameorBallet	-1.65	Cameor_multicrossVavD265CameorBallet	-2.38	Ballet_multicrossVavD265CameorBallet	4.03	-	-	-	-	-	-	-	-	-	-
LG1	Chr2	69.6	TSW08	4.40	0.06	69.60	61.64	93.20	-	-	Cameor_multicrossCameorChinaSommetteCerise	-3.81	-	-	-	-	-	-	China_multicrossCameorChinaSommetteCerise	-3.72	Sommette_multicrossCameorChinaSommetteCerise	4.85	Cerise_multicrossCameorChinaSommetteCerise	2.68
LG1	Chr2	73.5	SN09	5.02	0.10	73.30	71.22	78.52	-	-	Cameor_multicrossCameorChinaSommette	15.52	-	-	-	-	-	-	China_multicrossCameorChinaSommette	-30.32	Sommette_multicrossCameorChinaSommette	14.80	-	-
LG1	Chr2	75.4	TSW09	6.80	0.12	75.40	67.98	78.10	-	-	Cameor_multicrossCameorChinaSommette	-5.46	-	-	-	-	-	-	China_multicrossCameorChinaSommette	2.06	Sommette_multicrossCameorChinaSommette	3.40	-	-
LG1	Chr2	83.1	SW06	5.53	0.09	82.70	78.72	88.54	VavD265_multicrossVavD265CameorBallet	0.30	Cameor_multicrossVavD265CameorBallet	0.55	Ballet_multicrossVavD265CameorBallet	-0.84	-	-	-	-	-	-	-	-	-	-
LG1	Chr2	85.4	SN04	8.18	0.08	85.40	79.60	87.95	VavD265_multicrossVavD265CameorBallet	3.84	Cameor_multicrossVavD265CameorBallet	2.10	Ballet_multicrossVavD265CameorBallet	-5.94	-	-	-	-	-	-	-	-	-	-
LG1	Chr2	85.9	SW04	9.34	0.09	85.90	82.19	88.34	VavD265_multicrossVavD265CameorBallet	0.73	Cameor_multicrossVavD265CameorBallet	0.64	Ballet_multicrossVavD265CameorBallet	-1.37	-	-	-	-	-	-	-	-	-	-
LG2	Chr6	39.2	SW04	4.66	0.04	39.20	35.98	45.69	VavD265_multicrossVavD265CameorBallet	-0.69	Cameor_multicrossVavD265CameorBallet	-0.26	Ballet_multicrossVavD265CameorBallet	0.95	-	-	-	-	-	-	-	-	-	-
LG2	Chr6	42.9	SW06	10.39	0.16	42.90	38.24	46.13	VavD265_multicrossVavD265CameorBallet	-0.68	Cameor_multicrossVavD265CameorBallet	-0.70	Ballet_multicrossVavD265CameorBallet	1.38	-	-	-	-	-	-	-	-	-	-
LG2	Chr6	43.7	SN06	6.53	0.10	43.70	37.25	46.89	VavD265_multicrossVavD265CameorBallet	-3.44	Cameor_multicrossVavD265CameorBallet	-2.40	Ballet_multicrossVavD265CameorBallet	5.84	-	-	-	-	-	-	-	-	-	-
LG2	Chr6	44.6	SW11	4.28	0.04	44.60	26.39	51.42	VavD265_multicrossVavD265CameorBalletKazarChinaSommetteCeriseMelrose	0.03	Cameor_multicrossVavD265CameorBalletKazarChinaSommetteCeriseMelrose	-0.54	Ballet_multicrossVavD265CameorBalletKazarChinaSommetteCeriseMelrose	0.30	Melrose_multicrossVavD265CameorBalletKazarChinaSommetteCeriseMelrose	1.74	Kazar_multicrossVavD265CameorBalletKazarChinaSommetteCeriseMelrose	-0.04	China_multicrossVavD265CameorBalletKazarChinaSommetteCeriseMelrose	0.05	Sommette_multicrossVavD265CameorBalletKazarChinaSommetteCeriseMelrose	-0.37	Cerise_multicrossVavD265CameorBalletKazarChinaSommetteCeriseMelrose	-1.16
LG2	Chr6	51.5	TSW04	7.30	0.07	51.50	45.34	56.76	VavD265_multicrossVavD265CameorBallet	-2.34	Cameor_multicrossVavD265CameorBallet	-2.19	Ballet_multicrossVavD265CameorBallet	4.52	-	-	-	-	-	-	-	-	-	-
LG2	Chr6	55.6	TSW08	8.37	0.11	55.60	47.77	74.01	-	-	Cameor_multicrossCameorChinaSommetteCerise	-6.20	-	-	-	-	-	-	China_multicrossCameorChinaSommetteCerise	-2.27	Sommette_multicrossCameorChinaSommetteCerise	2.74	Cerise_multicrossCameorChinaSommetteCerise	5.73
LG2	Chr6	57.4	TSW09	6.90	0.13	57.40	51.44	73.50	-	-	Cameor_multicrossCameorChinaSommette	-4.88	-	-	-	-	-	-	China_multicrossCameorChinaSommette	-0.20	Sommette_multicrossCameorChinaSommette	5.08	-	-
LG2	Chr6	87.4	TSW11	9.45	0.08	87.40	84.17	93.18	VavD265_multicrossVavD265CameorBalletKazarChinaSommetteCeriseMelrose	-5.72	Cameor_multicrossVavD265CameorBalletKazarChinaSommetteCeriseMelrose	-2.66	Ballet_multicrossVavD265CameorBalletKazarChinaSommetteCeriseMelrose	-9.27	Melrose_multicrossVavD265CameorBalletKazarChinaSommetteCeriseMelrose	1.87	Kazar_multicrossVavD265CameorBalletKazarChinaSommetteCeriseMelrose	11.31	China_multicrossVavD265CameorBalletKazarChinaSommetteCeriseMelrose	0.53	Sommette_multicrossVavD265CameorBalletKazarChinaSommetteCeriseMelrose	-2.29	Cerise_multicrossVavD265CameorBalletKazarChinaSommetteCeriseMelrose	6.22
LG2	Chr6	98.4	SW11	4.01	0.04	98.40	79.80	102.96	VavD265_multicrossVavD265CameorBalletKazarChinaSommetteCeriseMelrose	-0.38	Cameor_multicrossVavD265CameorBalletKazarChinaSommetteCeriseMelrose	0.14	Ballet_multicrossVavD265CameorBalletKazarChinaSommetteCeriseMelrose	-0.35	Melrose_multicrossVavD265CameorBalletKazarChinaSommetteCeriseMelrose	0.42	Kazar_multicrossVavD265CameorBalletKazarChinaSommetteCeriseMelrose	2.33	China_multicrossVavD265CameorBalletKazarChinaSommetteCeriseMelrose	-0.76	Sommette_multicrossVavD265CameorBalletKazarChinaSommetteCeriseMelrose	-1.36	Cerise_multicrossVavD265CameorBalletKazarChinaSommetteCeriseMelrose	-0.05
LG3	Chr5	4.2	TSW04	7.02	0.07	3.50	0.00	13.28	VavD265_multicrossVavD265CameorBallet	-3.04	Cameor_multicrossVavD265CameorBallet	4.18	Ballet_multicrossVavD265CameorBallet	-1.13	-	-	-	-	-	-	-	-	-	-
LG3	Chr5	10.1	TSW06	7.10	0.11	10.10	4.39	12.92	VavD265_multicrossVavD265CameorBallet	-2.50	Cameor_multicrossVavD265CameorBallet	3.97	Ballet_multicrossVavD265CameorBallet	-1.46	-	-	-	-	-	-	-	-	-	-
LG3	Chr5	21.0	SW08	16.50	0.20	20.80	16.96	24.70	-	-	Cameor_multicrossCameorChinaSommetteCerise	-1.64	-	-	-	-	-	-	China_multicrossCameorChinaSommetteCerise	4.11	Sommette_multicrossCameorChinaSommetteCerise	-1.75	Cerise_multicrossCameorChinaSommetteCerise	-0.72
LG3	Chr5	24.1	SN08	29.27	0.32	24.10	21.10	25.58	-	-	Cameor_multicrossCameorChinaSommetteCerise	-11.38	-	-	-	-	-	-	China_multicrossCameorChinaSommetteCerise	29.28	Sommette_multicrossCameorChinaSommetteCerise	-11.28	Cerise_multicrossCameorChinaSommetteCerise	-6.63
LG3	Chr5	24.1	SPC11	10.27	0.09	24.10	15.30	26.95	VavD265_multicrossVavD265CameorBalletMelroseKazarChinaSommette	-0.11	Cameor_multicrossVavD265CameorBalletMelroseKazarChinaSommette	-0.12	Ballet_multicrossVavD265CameorBalletMelroseKazarChinaSommette	-0.10	Melrose_multicrossVavD265CameorBalletMelroseKazarChinaSommette	0.59	Kazar_multicrossVavD265CameorBalletMelroseKazarChinaSommette	-0.33	China_multicrossVavD265CameorBalletMelroseKazarChinaSommette	0.05	Sommette_multicrossVavD265CameorBalletMelroseKazarChinaSommette	0.04	-	-
LG3	Chr5	25.1	TSW08	10.21	0.13	25.10	18.04	29.28	-	-	Cameor_multicrossCameorChinaSommetteCerise	4.57	-	-	-	-	-	-	China_multicrossCameorChinaSommetteCerise	-8.15	Sommette_multicrossCameorChinaSommetteCerise	11.07	Cerise_multicrossCameorChinaSommetteCerise	-7.49
LG3	Chr5	25.6	TSW11	29.81	0.19	25.60	23.74	27.69	VavD265_multicrossVavD265CameorBalletKazarChinaSommetteCeriseMelrose	5.06	Cameor_multicrossVavD265CameorBalletKazarChinaSommetteCeriseMelrose	6.77	Ballet_multicrossVavD265CameorBalletKazarChinaSommetteCeriseMelrose	3.97	Melrose_multicrossVavD265CameorBalletKazarChinaSommetteCeriseMelrose	-9.98	Kazar_multicrossVavD265CameorBalletKazarChinaSommetteCeriseMelrose	7.97	China_multicrossVavD265CameorBalletKazarChinaSommetteCeriseMelrose	-9.54	Sommette_multicrossVavD265CameorBalletKazarChinaSommetteCeriseMelrose	7.99	Cerise_multicrossVavD265CameorBalletKazarChinaSommetteCeriseMelrose	-12.23
LG3	Chr5	26.4	SN09	12.63	0.23	26.40	24.38	29.10	-	-	Cameor_multicrossCameorChinaSommette	-25.75	-	-	-	-	-	-	China_multicrossCameorChinaSommette	51.31	Sommette_multicrossCameorChinaSommette	-25.56	-	-
LG3	Chr5	26.4	SN11	7.52	0.06	26.40	20.82	28.00	VavD265_multicrossVavD265CameorBalletKazarChinaSommetteCeriseMelrose	1.50	Cameor_multicrossVavD265CameorBalletKazarChinaSommetteCeriseMelrose	-4.40	Ballet_multicrossVavD265CameorBalletKazarChinaSommetteCeriseMelrose	-0.65	Melrose_multicrossVavD265CameorBalletKazarChinaSommetteCeriseMelrose	-10.36	Kazar_multicrossVavD265CameorBalletKazarChinaSommetteCeriseMelrose	3.57	China_multicrossVavD265CameorBalletKazarChinaSommetteCeriseMelrose	4.28	Sommette_multicrossVavD265CameorBalletKazarChinaSommetteCeriseMelrose	-0.61	Cerise_multicrossVavD265CameorBalletKazarChinaSommetteCeriseMelrose	6.67
LG3	Chr5	26.4	SW11	12.66	0.09	26.40	23.98	27.37	VavD265_multicrossVavD265CameorBalletKazarChinaSommetteCeriseMelrose	0.45	Cameor_multicrossVavD265CameorBalletKazarChinaSommetteCeriseMelrose	-0.32	Ballet_multicrossVavD265CameorBalletKazarChinaSommetteCeriseMelrose	-0.19	Melrose_multicrossVavD265CameorBalletKazarChinaSommetteCeriseMelrose	-2.88	Kazar_multicrossVavD265CameorBalletKazarChinaSommetteCeriseMelrose	0.41	China_multicrossVavD265CameorBalletKazarChinaSommetteCeriseMelrose	-0.14	Sommette_multicrossVavD265CameorBalletKazarChinaSommetteCeriseMelrose	0.32	Cerise_multicrossVavD265CameorBalletKazarChinaSommetteCeriseMelrose	2.36
LG3	Chr5	27.8	SW09	8.41	0.15	27.80	23.74	30.78	-	-	Cameor_multicrossCameorChinaSommette	-3.79	-	-	-	-	-	-	China_multicrossCameorChinaSommette	7.38	Sommette_multicrossCameorChinaSommette	-3.59	-	-
LG3	Chr5	27.8	TSW09	7.75	0.14	27.80	23.23	31.54	-	-	Cameor_multicrossCameorChinaSommette	1.66	-	-	-	-	-	-	China_multicrossCameorChinaSommette	-11.36	Sommette_multicrossCameorChinaSommette	9.70	-	-
LG3	Chr5	56.0	SPC10	4.62	0.15	56.00	39.08	57.54	-	-	Cameor_crossChinaCameor	0.28	-	-	-	-	-	-	China_crossChinaCameor	-0.28	-	-	-	-
LG3	Chr5	61.2	SPC08	7.03	0.12	61.20	53.15	64.61	-	-	Cameor_multicrossCameorChinaSommette	0.42	-	-	-	-	-	-	China_multicrossCameorChinaSommette	-0.09	Sommette_multicrossCameorChinaSommette	-0.34	-	-
LG3	Chr5	66.4	SPC06	4.60	0.08	66.40	61.02	71.46	VavD265_multicrossVavD265CameorBallet	0.23	Cameor_multicrossVavD265CameorBallet	0.10	Ballet_multicrossVavD265CameorBallet	-0.33	-	-	-	-	-	-	-	-	-	-
LG3	Chr5	73.3	SW06	5.02	0.08	73.30	60.75	78.33	VavD265_multicrossVavD265CameorBallet	-0.51	Cameor_multicrossVavD265CameorBallet	-0.43	Ballet_multicrossVavD265CameorBallet	0.94	-	-	-	-	-	-	-	-	-	-
LG3	Chr5	102.7	TSW04	7.69	0.07	102.70	101.15	108.86	VavD265_multicrossVavD265CameorBallet	-2.46	Cameor_multicrossVavD265CameorBallet	-2.43	Ballet_multicrossVavD265CameorBallet	4.89	-	-	-	-	-	-	-	-	-	-
LG3	Chr5	127.9	SPC06	12.73	0.20	127.90	125.08	130.66	VavD265_multicrossVavD265CameorBallet	-0.41	Cameor_multicrossVavD265CameorBallet	0.28	Ballet_multicrossVavD265CameorBallet	0.13	-	-	-	-	-	-	-	-	-	-
LG3	Chr5	128.2	SPC11	7.93	0.07	128.20	124.48	131.63	VavD265_multicrossVavD265CameorBalletMelroseKazarChinaSommette	-0.37	Cameor_multicrossVavD265CameorBalletMelroseKazarChinaSommette	0.24	Ballet_multicrossVavD265CameorBalletMelroseKazarChinaSommette	0.04	Melrose_multicrossVavD265CameorBalletMelroseKazarChinaSommette	-0.21	Kazar_multicrossVavD265CameorBalletMelroseKazarChinaSommette	0.48	China_multicrossVavD265CameorBalletMelroseKazarChinaSommette	0.09	Sommette_multicrossVavD265CameorBalletMelroseKazarChinaSommette	-0.27	-	-
LG3	Chr5	131.3	SN08	37.90	0.39	131.30	130.89	132.13	-	-	Cameor_multicrossCameorChinaSommetteCerise	-12.30	-	-	-	-	-	-	China_multicrossCameorChinaSommetteCerise	32.34	Sommette_multicrossCameorChinaSommetteCerise	-12.79	Cerise_multicrossCameorChinaSommetteCerise	-7.25
LG3	Chr5	131.3	SPC10	6.84	0.22	131.30	122.40	134.12	-	-	Cameor_crossChinaCameor	0.35	-	-	-	-	-	-	China_crossChinaCameor	-0.35	-	-	-	-
LG3	Chr5	131.3	TSW08	6.01	0.08	131.30	121.71	134.40	-	-	Cameor_multicrossCameorChinaSommetteCerise	4.03	-	-	-	-	-	-	China_multicrossCameorChinaSommetteCerise	-0.29	Sommette_multicrossCameorChinaSommetteCerise	5.21	Cerise_multicrossCameorChinaSommetteCerise	-8.95
LG3	Chr5	131.8	SN10	5.59	0.18	131.80	115.53	134.29	-	-	Cameor_crossChinaCameor	-22.60	-	-	-	-	-	-	China_crossChinaCameor	22.60	-	-	-	-
LG3	Chr5	131.8	SW06	20.64	0.29	132.30	130.63	133.81	VavD265_multicrossVavD265CameorBallet	1.86	Cameor_multicrossVavD265CameorBallet	-0.93	Ballet_multicrossVavD265CameorBallet	-0.93	-	-	-	-	-	-	-	-	-	-
LG3	Chr5	131.8	SW08	26.37	0.30	131.30	130.69	132.49	-	-	Cameor_multicrossCameorChinaSommetteCerise	-2.05	-	-	-	-	-	-	China_multicrossCameorChinaSommetteCerise	4.88	Sommette_multicrossCameorChinaSommetteCerise	-1.98	Cerise_multicrossCameorChinaSommetteCerise	-0.85
LG3	Chr5	131.8	SW10	6.28	0.21	131.80	130.33	134.40	-	-	Cameor_crossChinaCameor	-4.05	-	-	-	-	-	-	China_crossChinaCameor	4.05	-	-	-	-
LG3	Chr5	132.3	SN04	24.97	0.21	132.30	130.98	133.46	VavD265_multicrossVavD265CameorBallet	10.87	Cameor_multicrossVavD265CameorBallet	-6.48	Ballet_multicrossVavD265CameorBallet	-4.39	-	-	-	-	-	-	-	-	-	-
LG3	Chr5	132.3	SN11	27.21	0.17	132.30	131.18	134.11	VavD265_multicrossVavD265CameorBalletKazarChinaSommetteCeriseMelrose	20.71	Cameor_multicrossVavD265CameorBalletKazarChinaSommetteCeriseMelrose	-3.72	Ballet_multicrossVavD265CameorBalletKazarChinaSommetteCeriseMelrose	1.55	Melrose_multicrossVavD265CameorBalletKazarChinaSommetteCeriseMelrose	18.29	Kazar_multicrossVavD265CameorBalletKazarChinaSommetteCeriseMelrose	-9.75	China_multicrossVavD265CameorBalletKazarChinaSommetteCeriseMelrose	-19.72	Sommette_multicrossVavD265CameorBalletKazarChinaSommetteCeriseMelrose	-8.72	Cerise_multicrossVavD265CameorBalletKazarChinaSommetteCeriseMelrose	1.34
LG3	Chr5	132.3	SW04	30.85	0.26	132.30	131.14	133.29	VavD265_multicrossVavD265CameorBallet	2.61	Cameor_multicrossVavD265CameorBallet	-1.32	Ballet_multicrossVavD265CameorBallet	-1.28	-	-	-	-	-	-	-	-	-	-
LG3	Chr5	132.8	SW11	16.13	0.11	132.80	130.89	134.19	VavD265_multicrossVavD265CameorBalletKazarChinaSommetteCeriseMelrose	4.21	Cameor_multicrossVavD265CameorBalletKazarChinaSommetteCeriseMelrose	-0.80	Ballet_multicrossVavD265CameorBalletKazarChinaSommetteCeriseMelrose	-0.17	Melrose_multicrossVavD265CameorBalletKazarChinaSommetteCeriseMelrose	2.15	Kazar_multicrossVavD265CameorBalletKazarChinaSommetteCeriseMelrose	-1.69	China_multicrossVavD265CameorBalletKazarChinaSommetteCeriseMelrose	-2.78	Sommette_multicrossVavD265CameorBalletKazarChinaSommetteCeriseMelrose	-1.82	Cerise_multicrossVavD265CameorBalletKazarChinaSommetteCeriseMelrose	0.89
LG3	Chr5	132.8	TSW04	10.59	0.10	132.30	129.78	134.40	VavD265_multicrossVavD265CameorBallet	5.10	Cameor_multicrossVavD265CameorBallet	-0.52	Ballet_multicrossVavD265CameorBallet	-4.58	-	-	-	-	-	-	-	-	-	-
LG3	Chr5	133.5	SN06	20.92	0.29	133.50	130.97	134.40	VavD265_multicrossVavD265CameorBallet	9.28	Cameor_multicrossVavD265CameorBallet	-5.05	Ballet_multicrossVavD265CameorBallet	-4.23	-	-	-	-	-	-	-	-	-	-
LG4	Chr4	6.8	SW08	4.84	0.07	6.50	3.36	9.64	-	-	Cameor_multicrossCameorChinaSommetteCerise	0.69	-	-	-	-	-	-	China_multicrossCameorChinaSommetteCerise	-2.21	Sommette_multicrossCameorChinaSommetteCerise	1.02	Cerise_multicrossCameorChinaSommetteCerise	0.50
LG4	Chr4	18.4	SPC04	4.64	0.04	18.40	6.02	21.70	VavD265_multicrossVavD265CameorBallet	0.19	Cameor_multicrossVavD265CameorBallet	-0.17	Ballet_multicrossVavD265CameorBallet	-0.01	-	-	-	-	-	-	-	-	-	-
LG4	Chr4	22.8	SPC11	6.21	0.06	22.80	12.38	28.14	VavD265_multicrossVavD265CameorBalletMelroseKazarChinaSommette	0.09	Cameor_multicrossVavD265CameorBalletMelroseKazarChinaSommette	-0.11	Ballet_multicrossVavD265CameorBalletMelroseKazarChinaSommette	0.11	Melrose_multicrossVavD265CameorBalletMelroseKazarChinaSommette	0.57	Kazar_multicrossVavD265CameorBalletMelroseKazarChinaSommette	0.18	China_multicrossVavD265CameorBalletMelroseKazarChinaSommette	-0.35	Sommette_multicrossVavD265CameorBalletMelroseKazarChinaSommette	-0.50	-	-
LG4	Chr4	22.8	TSW06	5.44	0.09	22.80	19.68	24.97	VavD265_multicrossVavD265CameorBallet	-2.49	Cameor_multicrossVavD265CameorBallet	-2.50	Ballet_multicrossVavD265CameorBallet	4.99	-	-	-	-	-	-	-	-	-	-
LG4	Chr4	49.3	SPC06	5.78	0.09	49.30	44.94	53.32	VavD265_multicrossVavD265CameorBallet	0.40	Cameor_multicrossVavD265CameorBallet	0.04	Ballet_multicrossVavD265CameorBallet	-0.38	-	-	-	-	-	-	-	-	-	-
LG4	Chr4	57.2	SPC04	7.64	0.07	57.20	48.39	66.99	VavD265_multicrossVavD265CameorBallet	-0.03	Cameor_multicrossVavD265CameorBallet	0.25	Ballet_multicrossVavD265CameorBallet	-0.23	-	-	-	-	-	-	-	-	-	-
LG4	Chr4	77.4	TSW11	9.99	0.08	77.40	73.25	85.23	VavD265_multicrossVavD265CameorBalletKazarChinaSommetteCeriseMelrose	-2.69	Cameor_multicrossVavD265CameorBalletKazarChinaSommetteCeriseMelrose	-0.33	Ballet_multicrossVavD265CameorBalletKazarChinaSommetteCeriseMelrose	4.43	Melrose_multicrossVavD265CameorBalletKazarChinaSommetteCeriseMelrose	-3.11	Kazar_multicrossVavD265CameorBalletKazarChinaSommetteCeriseMelrose	-5.47	China_multicrossVavD265CameorBalletKazarChinaSommetteCeriseMelrose	-7.04	Sommette_multicrossVavD265CameorBalletKazarChinaSommetteCeriseMelrose	-2.26	Cerise_multicrossVavD265CameorBalletKazarChinaSommetteCeriseMelrose	16.48
LG4	Chr4	83.6	TSW04	7.29	0.07	83.60	73.08	88.21	VavD265_multicrossVavD265CameorBallet	-4.31	Cameor_multicrossVavD265CameorBallet	0.87	Ballet_multicrossVavD265CameorBallet	3.44	-	-	-	-	-	-	-	-	-	-
LG4	Chr4	84.1	SPC06	5.97	0.10	84.10	76.57	93.44	VavD265_multicrossVavD265CameorBallet	0.15	Cameor_multicrossVavD265CameorBallet	-0.24	Ballet_multicrossVavD265CameorBallet	0.08	-	-	-	-	-	-	-	-	-	-
LG4	Chr4	105.4	SPC11	4.34	0.04	105.40	102.00	114.70	VavD265_multicrossVavD265CameorBalletMelroseKazarChinaSommette	0.17	Cameor_multicrossVavD265CameorBalletMelroseKazarChinaSommette	0.25	Ballet_multicrossVavD265CameorBalletMelroseKazarChinaSommette	0.11	Melrose_multicrossVavD265CameorBalletMelroseKazarChinaSommette	-0.33	Kazar_multicrossVavD265CameorBalletMelroseKazarChinaSommette	-0.14	China_multicrossVavD265CameorBalletMelroseKazarChinaSommette	-0.30	Sommette_multicrossVavD265CameorBalletMelroseKazarChinaSommette	0.23	-	-
LG5	Chr3	10.9	TSW04	5.88	0.06	10.40	0.00	21.86	VavD265_multicrossVavD265CameorBallet	0.56	Cameor_multicrossVavD265CameorBallet	-3.69	Ballet_multicrossVavD265CameorBallet	3.13	-	-	-	-	-	-	-	-	-	-
LG5	Chr3	21.1	TSW06	8.57	0.14	21.10	11.86	23.52	VavD265_multicrossVavD265CameorBallet	0.16	Cameor_multicrossVavD265CameorBallet	-4.23	Ballet_multicrossVavD265CameorBallet	4.08	-	-	-	-	-	-	-	-	-	-
LG5	Chr3	55.9	SPC06	8.13	0.13	55.90	48.74	58.33	VavD265_multicrossVavD265CameorBallet	0.11	Cameor_multicrossVavD265CameorBallet	0.23	Ballet_multicrossVavD265CameorBallet	-0.35	-	-	-	-	-	-	-	-	-	-
LG5	Chr3	96.7	TSW04	6.68	0.06	97.20	90.63	113.30	VavD265_multicrossVavD265CameorBallet	0.10	Cameor_multicrossVavD265CameorBallet	-3.82	Ballet_multicrossVavD265CameorBallet	3.73	-	-	-	-	-	-	-	-	-	-
LG5	Chr3	110.6	TSW06	7.97	0.13	113.30	104.56	113.30	VavD265_multicrossVavD265CameorBallet	-0.14	Cameor_multicrossVavD265CameorBallet	-4.04	Ballet_multicrossVavD265CameorBallet	4.18	-	-	-	-	-	-	-	-	-	-
LG5	Chr3	113.3	SPC06	11.17	0.17	113.30	110.80	113.30	VavD265_multicrossVavD265CameorBallet	0.15	Cameor_multicrossVavD265CameorBallet	-0.33	Ballet_multicrossVavD265CameorBallet	0.17	-	-	-	-	-	-	-	-	-	-
LG6	Chr1	13.9	SN11	4.05	0.04	13.90	9.71	19.71	VavD265_multicrossVavD265CameorBalletKazarChinaSommetteCeriseMelrose	4.33	Cameor_multicrossVavD265CameorBalletKazarChinaSommetteCeriseMelrose	2.56	Ballet_multicrossVavD265CameorBalletKazarChinaSommetteCeriseMelrose	-1.41	Melrose_multicrossVavD265CameorBalletKazarChinaSommetteCeriseMelrose	0.27	Kazar_multicrossVavD265CameorBalletKazarChinaSommetteCeriseMelrose	-8.55	China_multicrossVavD265CameorBalletKazarChinaSommetteCeriseMelrose	-7.20	Sommette_multicrossVavD265CameorBalletKazarChinaSommetteCeriseMelrose	8.70	Cerise_multicrossVavD265CameorBalletKazarChinaSommetteCeriseMelrose	1.31
LG6	Chr1	47.7	TSW11	7.97	0.07	47.70	43.61	51.27	VavD265_multicrossVavD265CameorBalletKazarChinaSommetteCeriseMelrose	1.99	Cameor_multicrossVavD265CameorBalletKazarChinaSommetteCeriseMelrose	0.99	Ballet_multicrossVavD265CameorBalletKazarChinaSommetteCeriseMelrose	2.50	Melrose_multicrossVavD265CameorBalletKazarChinaSommetteCeriseMelrose	-7.19	Kazar_multicrossVavD265CameorBalletKazarChinaSommetteCeriseMelrose	-6.39	China_multicrossVavD265CameorBalletKazarChinaSommetteCeriseMelrose	-8.33	Sommette_multicrossVavD265CameorBalletKazarChinaSommetteCeriseMelrose	0.16	Cerise_multicrossVavD265CameorBalletKazarChinaSommetteCeriseMelrose	16.28
LG6	Chr1	49.6	SPC10	3.64	0.11	49.60	42.36	62.80	-	-	Cameor_crossChinaCameor	-0.25	-	-	-	-	-	-	China_crossChinaCameor	0.25	-	-	-	-
LG6	Chr1	81.4	TSW06	5.81	0.10	81.30	70.20	87.94	VavD265_multicrossVavD265CameorBallet	-5.74	Cameor_multicrossVavD265CameorBallet	-0.41	Ballet_multicrossVavD265CameorBallet	6.15	-	-	-	-	-	-	-	-	-	-
LG6	Chr1	94.2	TSW10	4.31	0.14	94.20	77.98	101.00	-	-	Cameor_ChinaCameor	-3.75	-	-	-	-	-	-	China_ChinaCameor	3.75	-	-	-	-
LG7	Chr7	10.1	SPC10	4.25	0.13	10.10	0.00	17.37	-	-	Cameor_crossChinaCameor	0.26	-	-	-	-	-	-	China_crossChinaCameor	-0.26	-	-	-	-
LG7	Chr7	20.8	SN08	4.87	0.07	20.80	17.77	24.33	-	-	Cameor_multicrossCameorChinaSommetteCerise	-4.29	-	-	-	-	-	-	China_multicrossCameorChinaSommetteCerise	11.23	Sommette_multicrossCameorChinaSommetteCerise	-4.38	Cerise_multicrossCameorChinaSommetteCerise	-2.56
LG7	Chr7	20.8	SPC08	6.83	0.12	20.80	19.44	22.69	-	-	Cameor_multicrossCameorChinaSommette	0.38	-	-	-	-	-	-	China_multicrossCameorChinaSommette	-0.46	Sommette_multicrossCameorChinaSommette	0.08	-	-
LG7	Chr7	45.2	TSW09	4.78	0.09	45.20	41.99	61.17	-	-	Cameor_multicrossCameorChinaSommette	0.17	-	-	-	-	-	-	China_multicrossCameorChinaSommette	-7.05	Sommette_multicrossCameorChinaSommette	6.88	-	-
LG7	Chr7	56.7	TSW11	8.23	0.07	56.70	54.46	64.01	VavD265_multicrossVavD265CameorBalletKazarChinaSommetteCeriseMelrose	-10.07	Cameor_multicrossVavD265CameorBalletKazarChinaSommetteCeriseMelrose	3.66	Ballet_multicrossVavD265CameorBalletKazarChinaSommetteCeriseMelrose	-4.09	Melrose_multicrossVavD265CameorBalletKazarChinaSommetteCeriseMelrose	-1.53	Kazar_multicrossVavD265CameorBalletKazarChinaSommetteCeriseMelrose	-4.17	China_multicrossVavD265CameorBalletKazarChinaSommetteCeriseMelrose	0.18	Sommette_multicrossVavD265CameorBalletKazarChinaSommetteCeriseMelrose	7.51	Cerise_multicrossVavD265CameorBalletKazarChinaSommetteCeriseMelrose	8.51
LG7	Chr7	58.1	SN06	6.68	0.11	58.10	56.08	61.39	VavD265_multicrossVavD265CameorBallet	3.24	Cameor_multicrossVavD265CameorBallet	-3.51	Ballet_multicrossVavD265CameorBallet	0.27	-	-	-	-	-	-	-	-	-	-
LG7	Chr7	58.1	TSW06	8.76	0.14	58.60	52.84	59.42	VavD265_multicrossVavD265CameorBallet	-3.26	Cameor_multicrossVavD265CameorBallet	4.28	Ballet_multicrossVavD265CameorBallet	-1.02	-	-	-	-	-	-	-	-	-	-
LG7	Chr7	58.6	TSW04	17.24	0.16	58.60	58.00	59.00	VavD265_multicrossVavD265CameorBallet	-4.88	Cameor_multicrossVavD265CameorBallet	6.78	Ballet_multicrossVavD265CameorBallet	-1.90	-	-	-	-	-	-	-	-	-	-
LG7	Chr7	71.4	SN11	9.97	0.08	71.40	70.00	82.13	VavD265_multicrossVavD265CameorBalletKazarChinaSommetteCeriseMelrose	5.41	Cameor_multicrossVavD265CameorBalletKazarChinaSommetteCeriseMelrose	1.35	Ballet_multicrossVavD265CameorBalletKazarChinaSommetteCeriseMelrose	9.42	Melrose_multicrossVavD265CameorBalletKazarChinaSommetteCeriseMelrose	-7.56	Kazar_multicrossVavD265CameorBalletKazarChinaSommetteCeriseMelrose	2.37	China_multicrossVavD265CameorBalletKazarChinaSommetteCeriseMelrose	8.31	Sommette_multicrossVavD265CameorBalletKazarChinaSommetteCeriseMelrose	-21.46	Cerise_multicrossVavD265CameorBalletKazarChinaSommetteCeriseMelrose	2.15
LG7	Chr7	75.7	SPC08	7.01	0.12	75.70	70.20	100.45	-	-	Cameor_multicrossCameorChinaSommette	-0.36	-	-	-	-	-	-	China_multicrossCameorChinaSommette	-0.06	Sommette_multicrossCameorChinaSommette	0.41	-	-
LG7	Chr7	77.8	SW11	8.78	0.07	77.80	70.08	82.34	VavD265_multicrossVavD265CameorBalletKazarChinaSommetteCeriseMelrose	0.34	Cameor_multicrossVavD265CameorBalletKazarChinaSommetteCeriseMelrose	0.49	Ballet_multicrossVavD265CameorBalletKazarChinaSommetteCeriseMelrose	1.97	Melrose_multicrossVavD265CameorBalletKazarChinaSommetteCeriseMelrose	-0.68	Kazar_multicrossVavD265CameorBalletKazarChinaSommetteCeriseMelrose	-0.20	China_multicrossVavD265CameorBalletKazarChinaSommetteCeriseMelrose	1.24	Sommette_multicrossVavD265CameorBalletKazarChinaSommetteCeriseMelrose	-3.98	Cerise_multicrossVavD265CameorBalletKazarChinaSommetteCeriseMelrose	0.81
LG7	Chr7	88.6	TSW04	4.89	0.05	88.60	79.88	92.95	VavD265_multicrossVavD265CameorBallet	-1.34	Cameor_multicrossVavD265CameorBallet	-2.49	Ballet_multicrossVavD265CameorBallet	3.83	-	-	-	-	-	-	-	-	-	-

[a] QTL position from the first marker of the linkage group (in cM Haldane)

[b] *SN* seed number per plant, *SPC* seed protein content (% of seed dry weight), *SW* seed weight per plant (g), *TSW* thousand seed weight (g), *04* 2004, *06* 2006, *08* 2008, *09* 2009, *10* 2010, *11* 2011

[c] *P* peak value at the QTL position for each variable

[d] Phenotypic variance explained by each QTL

[e] Position of the lower and upper ends of the QTL confidence intervals from the first marker of the linkage group (in cM Haldane)

[f] Allelic value of parental lines
