# Supplementary material for: Meta-analysis of QTL reveals the genetic control of yield-related traits and seed protein content in pea
Source: Sci Rep. 2020 Sep 28;10:15925. doi: 10.1038/s41598-020-72548-9 (PMC7522997; doi:10.1038/s41598-020-72548-9)
Supplement: Supplementary file 7 — Supplementary Table 4. [file 41598_2020_72548_MOESM7_ESM.pdf]

Meta-analysis of QTL reveals the genetic control of yield-related traits and seed protein content in pea

Anthony Klein[1*], Hervé Houtin[1], Céline Rond-Coissieux[1], Myriam Naudet-Huart[1], Michael Touratier[1], Pascal Marget[2,1] and Judith Burstin[1]

[1] Agroécologie, AgroSup Dijon, INRAE, Univ. Bourgogne, Univ. Bourgogne Franche-Comté, F-21000 Dijon, France

[2] INRAE, UE 0115 DIJ Domaine Expérimental d'Epoisses. Centre de recherche Bourgogne-Franche-Comté, F-21110 Breteniere, France

*** Correspondence:**
anthony.klein@inrae.fr

Table S4 : QTL prediction to metaQTL for seed weight (SW), seed number (SN), thousand seed weight (TSW) and seed protein content (SPC) from Pop3 to Pop11 between 2004 and 2011 at INRAE Dijon. For each linkage group, position (in cM Haldane) and confidence interval (CI) of metaQTL are indicated. The QTL prediction membership of metaQTL (mQTL) are calculated from 0 to 1.

LG1

	Position (cM)	*Weight*	*Distance*	*CI(95%) (cM)*
mQTL1.1	2.66	0.40	6.96	2.19
mQTL1.2	15.24	0.07	2.82	4.92
mQTL1.3	25.54	0.13	9.39	5.66
mQTL1.4	74.03	0.19	2.42	6.08
mQTL1.5	85.14	0.21	-	1.47

QTL Position - CI(95%) - Predicted Memberships mQTL1.1 --> mQTL1.5

QTL	*QTL position (cM)*	*CI(95%) (cM)*	*mQTL position (cM)*	*mQTL1.1*	*mQTL1.2*	*mQTL1.3*	*mQTL1.4*	*mQTL1.5*
SPC06-0.0	0.00	4.63	2.66	1	0	0	0	0
SN04-4.9	4.90	7.06	2.66	1	0	0	0	0
TSW06-2.9	2.90	2.27	2.66	1	0	0	0	0
TSW04-2.8	2.80	1.96	2.66	1	0	0	0	0
TSW11-2.3	2.30	2.98	2.66	1	0	0	0	0
SPC04-4.4	4.40	6.55	2.66	1	0	0	0	0
TSW06-15.2	15.20	4.59	15.24	0	1	0	0	0
TSW04-26.1	26.10	21.25	24.82	0	0.07	0.93	0	0
TSW08-25.5	25.50	5.57	25.54	0	0	1	0	0
SN09-73.5	73.50	7.29	74.03	0	0	0	1	0
TSW09-75.4	75.40	10.11	74.03	0	0	0	1	0
TSW08-69.6	69.60	31.56	75.92	0	0	0	0.83	0.17
SW06-83.1	83.10	9.84	85.14	0	0	0	0	1
SW04-85.9	85.90	6.15	85.14	0	0	0	0	1
SN04-85.4	85.40	8.35	85.14	0	0	0	0	1

LG2

	Position (cM)	*Weight*	*Distance*	*CI(95%) (cM)*
mQTL2.1	42.30	0.44	1.71	5.06
mQTL2.2	52.70	0.34	5.51	9.45
mQTL2.3	88.97	0.22	-	4.98

QTL Position - CI(95%) - Predicted Memberships mQTL2.1 --> mQTL2.3

QTL	*QTL position (cM)*	*CI(95%) (cM)*	*mQTL position (cM)*	*mQTL2.1*	*mQTL2.2*	*mQTL2.3*
SW06-42.9	42.90	7.88	42.30	1	0	0
SW04-39.2	39.20	9.72	42.30	1	0	0
SN06-43.7	43.70	9.64	42.30	1	0	0
SW11-44.6	44.60	25.05	45.11	0.73	0.27	0
TSW08-55.6	55.60	26.22	51.04	0.16	0.84	0
TSW09-57.4	57.40	22.07	52.18	0.05	0.95	0
TSW04-51.5	51.50	11.41	52.60	0.01	0.99	0
TSW11-87.4	87.40	9.45	88.97	0	0	1

SW11-98.4	98.40	23.17	88.97	0	0	1

LG3

	Position (cM)	Weight	Distance	CI(95%) (cM)
mQTL3.1	24.73	0.38	9.49	1.69
mQTL3.2	64.32	0.12	9.17	6.60
mQTL3.3	102.70	0.03	11.90	7.70
mQTL3.4	131.80	0.47	-	0.03

QTL Position - CI(95%) - Predicted Memberships mQTL3.1 --> mQTL3.4

QTL	*QTL position (cM)*	*CI(95%) (cM)*	*mQTL position (cM)*	*mQTL3.1*	*mQTL3.2*	*mQTL3.3*	*mQTL3.4*
TSW11-25.6	25.60	4.00	24.73	1	0	0	0
TSW09-27.8	27.80	8.31	24.73	1	0	0	0
SW09-27.8	27.80	7.06	24.73	1	0	0	0
SW11-26.4	26.40	3.37	24.73	1	0	0	0
SW08-21	21.00	7.72	24.73	1	0	0	0
SPC11-24.1	24.10	11.64	24.73	1	0	0	0
SN08-24.1	24.10	4.47	24.73	1	0	0	0
SN11-26.4	26.40	7.17	24.73	1	0	0	0
TSW08-25.1	25.10	11.25	24.73	1	0	0	0
TSW04-4.2	4.20	13.29	24.73	1	0	0	0
TSW06-10.1	10.10	8.55	24.73	1	0	0	0
SN09-26.4	26.40	4.70	24.73	1	0	0	0
SPC06-66.4	66.40	10.43	64.32	0	1	0	0
SPC08-61.2	61.20	11.45	64.32	0	1	0	0
SW06-73.3	73.30	17.56	64.32	0	1	0	0
SPC10-56.0	56.00	18.46	64.32	0	1	0	0
TSW04-102.7	102.70	7.72	102.70	0	0	1	0
SN08-131.3	131.30	1.25	131.80	0	0	0	1
SW06-131.8	131.80	3.18	131.80	0	0	0	1
SN10-131.8	131.80	18.74	131.80	0	0	0	1
SW04-132.3	132.30	2.16	131.80	0	0	0	1
SW10-131.8	131.80	4.08	131.80	0	0	0	1
TSW04-132.8	132.80	4.63	131.80	0	0	0	1
SPC11-128.2	128.20	7.13	131.80	0	0	0	1
SN11-132.3	132.30	2.94	131.80	0	0	0	1
SN04-132.3	132.30	2.47	131.80	0	0	0	1
SW08-131.8	131.80	1.80	131.80	0	0	0	1
SN06-133.5	133.50	3.45	131.80	0	0	0	1
SPC10-131.3	131.30	11.72	131.80	0	0	0	1
SPC06-127.9	127.90	5.57	131.80	0	0	0	1
TSW08-131.3	131.30	12.70	131.80	0	0	0	1
SW11-132.8	132.80	3.29	131.80	0	0	0	1

LG4

	Position (cM)	Weight	Distance	CI(95%) (cM)
mQTL4.1	6.82	0.10	4.43	6.30
mQTL4.2	22.39	0.30	6.08	4.80
mQTL4.3	50.63	0.20	5.93	7.64
mQTL4.4	80.94	0.30	4.91	8.36

mQTL4.5	105.40	0.10	-	10.50

QTL Position - CI(95%) - Predicted Memberships mQTL4.1 --> mQTL4.5

QTL	*QTL position (cM)*	*CI(95%) (cM)*	*mQTL position (cM)*	*mQTL4.1*	*mQTL4.2*	*mQTL4.3*	*mQTL4.4*	*mQTL4.5*
SW08-6.8	6.80	6.27	6.82	1	0	0	0	0
SPC04-18.4	18.40	15.68	22.23	0.01	0.99	0	0	0
SPC11-22.8	22.80	15.76	22.39	0	1	0	0	0
TSW06-22.8	22.80	5.29	22.39	0	1	0	0	0
SPC06-49.3	49.30	8.39	50.63	0	0	1	0	0
SPC04-57.2	57.20	18.58	50.63	0	0	1	0	0
SPC06-84.1	84.10	16.86	80.94	0	0	0	1	0
TSW11-77.4	77.40	12.47	80.94	0	0	0	1	0
TSW04-83.6	83.60	15.13	80.94	0	0	0	1	0
SPC11-105.4	105.40	12.70	105.40	0	0	0	0	1

LG5

	Position (cM)	*Weight*	*Distance*	*CI(95%) (cM)*
mQTL5.1	18.84	0.33	7.51	10.29
mQTL5.2	55.90	0.17	15.07	9.59
mQTL5.3	112.91	0.50	-	0.37

QTL Position - CI(95%) - Predicted Memberships mQTL5.1 --> mQTL5.3

QTL	*QTL position (cM)*	*CI(95%) (cM)*	*mQTL position (cM)*	*mQTL5.1*	*mQTL5.2*	*mQTL5.3*
TSW04-10.9	10.90	21.87	18.84	1	0	0
TSW06-21.1	21.10	11.68	18.84	1	0	0
SPC06-55.9	55.90	9.60	55.90	0	1	0
TSW04-96.7	96.70	22.66	112.91	0	0	1
TSW06-110.6	110.60	8.74	112.91	0	0	1
SPC06-113.3	113.30	2.51	112.91	0	0	1

LG6

	Position (cM)	*Weight*	*Distance*	*CI(95%) (cM)*
mQTL6.1	13.90	0.20	7.70	10.41
mQTL6.2	47.95	0.40	6.04	7.36
mQTL6.3	86.17	0.40	-	12.84

QTL Position - CI(95%) - Predicted Memberships mQTL6.1 --> mQTL6.3

QTL	*QTL position (cM)*	*CI(95%) (cM)*	*mQTL position (cM)*	*mQTL6.1*	*mQTL6.2*	*mQTL6.3*
SN11-13.9	13.90	10.00	13.90	1	0	0
TSW11-47.7	47.70	7.88	47.95	0	1	0
SPC10-49.6	49.60	20.42	47.95	0	1	0
TSW06-81.4	81.40	17.72	86.17	0	0	1
TSW10-94.2	94.20	23.01	86.17	0	0	1

LG7

	Position (cM)	*Weight*	*Distance*	*CI(95%) (cM)*
mQTL7.1	20.51	0.25	11.76	2.87
mQTL7.2	58.52	0.43	3.24	0.96
mQTL7.3	74.66	0.23	2.39	8.38
mQTL7.4	88.38	0.09	-	10.93

QTL Position - CI(95%) - Predicted Memberships mQTL7.1 --> mQTL7.4

QTL	*QTL position (cM)*	*CI(95%) (cM)*	*mQTL position (cM)*	*mQTL7.1*	*mQTL7.2*	*mQTL7.3*	*mQTL7.4*
SPC08-20.8	20.80	3.25	20.51	1	0	0	0
SPC10-10.1	10.10	17.37	20.51	1	0	0	0
SN08-20.8	20.80	6.55	20.51	1	0	0	0
TSW09-45.2	45.20	19.17	58.52	0	1	0	0
TSW06-58.1	58.10	6.59	58.52	0	1	0	0
TSW11-56.7	56.70	9.72	58.52	0	1	0	0
TSW04-58.6	58.60	0.98	58.52	0	1	0	0
SN06-58.1	58.10	5.29	58.52	0	1	0	0
SPC08-75.7	75.70	30.26	73.82	0	0.12	0.8	0.08
SW11-77.8	77.80	12.23	74.66	0	0	1	0
SN11-71.4	71.40	12.19	74.66	0	0	1	0
TSW04-88.6	88.60	13.05	88.38	0	0	0	1
