# Supplementary material for: Meta-analysis of QTL reveals the genetic control of yield-related traits and seed protein content in pea
Source: Sci Rep. 2020 Sep 28;10:15925. doi: 10.1038/s41598-020-72548-9 (PMC7522997; doi:10.1038/s41598-020-72548-9)
Supplement: Supplementary file 8 — Supplementary Table 5. [file 41598_2020_72548_MOESM8_ESM.pdf]

Meta-analysis of QTL reveals the genetic control of yield-related traits and seed protein content in pea

Anthony Klein[1*], Hervé Houtin[1], Céline Rond-Coissieux[1], Myriam Naudet-Huart[1], Michael Touratier[1], Pascal Marget[2,1] and Judith Burstin[1]

[1] Agroécologie, AgroSup Dijon, INRAE, Univ. Bourgogne, Univ. Bourgogne Franche-Comté, F-21000 Dijon, France

[2] INRAE, UE 0115 DIJ Domaine Expérimental d'Epoisses. Centre de recherche Bourgogne-Franche-Comté, F-21110 Breteniere, France

*** Correspondence:**
anthony.klein@inrae.fr

Table S5 : List of genes in pea metaQTL and homologous in soybean. For each pea metaQTL, peak gene and QTL references are identified. Soybean genes were researched from SoyBase[36] (http://www.soybase.org)

MetaQTL	Chromosome/ Linkage group	Peak position - peak gene (Kreplak *et al.*)[23]		annotation (Kreplak *et al.*)[23]	Position start - gene at start (Kreplak *et al.*)[23]		Position end - gene at end (Kreplak *et al.*)[23]		Number of genes in confidence interval (Kreplak *et al.*)[23]	Predicted metaQTL position (cM)	Meta QTL 95% genetic confidence interval (cM)	Flanking markers (Tayeh *et al*. - Table S10)[20]	Traits assignment to MetaQTL (Caméor allele effect sign)	QTL references in pea	Peak gene soybean homolog (SoyBase[36])	Gene model name correspondence (SoyBase[36])	Gene model in 50 kb region (SoyBase[36])	Marker in 50 kb region around gene (SoyBase[36])	QTL in 50 kb region around gene (SoyBase[36])	QTL in soybean Qi *et al.*[34]	QTL in soybean Karikari *et al.*[37]	QTL in soybean Li *et al.*[38]	QTL in soybean Zhang *et al*.[39]	QTL in soybean Zhang *et al*.[40]
mQTL1.1	chr2LG1	2980750	*Psat2g004160*	Protein kinase domain	454467	*Psat2g000640*	5774049	*Psat2g006680*	141	2.66	2.19	PsCam016921_10518_200 ; PsCam042632_26683_1732	SN04+, SPC04-, SPC04, SPC06-, TSW04-, TSW06-, TSW11-	SW - Moreau *et al*.[12] SPC - Gali *et al*.[13]	*Glyma02g47670.1*	*Glyma.02g306500*	*Gm02:48103548..48104600*					qProt-2-1 *Gm02_BLOCK_3424 1156 34302885*		
mQTL1.2	chr2LG1	23203491	*Psat2g021920*	Unknown gene	14937233	*Psat2g016080*	31547380	*Psat2g026280*	235	15.24	4.92	PsCam044143_28060_1723 ; PsCam000692 604 684	TSW06-		*Glyma02g45500.1*	*Glyma.02g285600*	*Gm02:46655788..46660367*							
mQTL1.3	chr2LG1	56661835	*Psat2g037640*	LIM domain	32549163	*Psat2g027160*	80822825	*Psat2g046440*	445	25.54	5.66	PsCam001049_894_1330 ; PsCam040389 25143 344	TSW04-, TSW08-		*Glyma02g41920.1*	*Glyma.02g251800*	*Gm02:43901083..43905853*							
mQTL1.4	chr2LG1	387065449	*Psat2g156040*	Pre-rRNA-processing protein TSR2	372862478	*Psat2g145000*	401022163	*Psat2g166320*	448	74.03	6.08	PsCam036267_21415_2674 ; PsCam046088 29624 710	SN09+, TSW08-, TSW09-	TSW - Burstin *et al*.[7]	*Glyma19g01230.1*	*Glyma.19g009300*	*Gm19:881893..883973*							
mQTL1.5	chr2LG1	411495507	*Psat2g172600*	Amino-transferase class IV	409802804	*Psat2g171720*	413236825	*Psat2g174040*	56	85.14	1.47	PsCam049764_32384_3938 ; PsCam047748 30645 563	SN04+, SW04+, SW06+	SN, SW, SPC - Burstin *et al.*[7]	*Glyma01g40420.1*	*Glyma.01g196300*	*Gm01:53062250..53067395*							
mQTL2.1	chr6LG2	114863224	*Psat6g082080*	Unknown gene	71913567	*Psat6g064200*	157742624	*Psat6g097360*	751	42.3	5.06	PsCam045557_29214_1582 ; PsCam037747 22816 1252	SN06-, SW04-, SW06-, SW11-	SW - Tar'an *et al.*[5]	*Glyma19g35380.1*	*Glyma.19g170400*	*Gm19:43115371..43118724*		GWAS Seed weight 9-g5.2 *(Gm19:43116996)*					
mQTL2.2	chr6LG2	189681633	*Psat6g111440*	Unknown gene	88985653	*Psat6g070840*	290738342	*Psat6g148120*	1761	52.7	9.45	PsCam034889_20236_728 ; PsCam050914 33467 786	TSW04-, TSW08-, TSW09-		*Glyma10g06070.1*	*Glyma.10g053300*	*Gm10:4820870..4822851*		Seed yield 28-9, 25-5, Seed protein 36-40					
mQTL2.3	chr6LG2	388681088	*Psat6g193240*	Plant organelle RNA recognition domain	369699084	*Psat6g183960*	404311257	*Psat6g203560*	457	88.97	4.98	PsCam050254_32847_1148 ; PsCam053661 35501 2019	SW11+, TSW11-		*Glyma10g37010.1*	*Glyma.10g225700*	*Gm10:45624849..45626090*							
mQTL3.1	chr5LG3	61462435	*Psat5g032160*	MIR domain	56764094	*Psat5g030080*	66228363	*Psat5g035840*	135	24.73	1.69	PsCam001506_1254_232 ; PsCam039566_24482_378	SN08-, SN09-, SN11-, SPC11-, SW08-, SW09-, SW11-, TSW04+, TSW06+, TSW08+, TSW09+, TSW11+	TSW - Burstin *et al.*[7] TSW - Gali *et al.*[13] TSW, SW - Klein *et al.*[10]	*Glyma04g06580.1*	*Glyma.04g061900*	*Gm04:5091573..5094607*			MQTL7,8,9,10	qPro-4-1	qProt-4-1 *Gm04_BLOCK_2157 397_2345500* ; qProt-4-2 *Gm04_8725710* ; qProt-4-3 *Gm04_BLOCK_3998 7192 40167695*		GqPC4-1 (34743951); GqPC4-2: *Glyma.04G157500 (46200673)*
mQTL3.2	chr5LG3	215141118	*Psat5g121200*	Unknown gene	191691654	*Psat5g107640*	239064211	*Psat5g133080*	583	64.32	6.6	PsCam036453_21593_1599 ; PsCam038194 23240 124	SPC06+, SPC08+, SPC10+, SW06-	SW, SPC - Gali *et al*.[13]	*Glyma01g03920.1*	*Glyma.01g032400*	*Gm01:3405114..3411252*				qPro-1-I	qProt-1-1 *Gm01 50257226*	qPro1-1	
mQTL3.3	chr5LG3	467013019	*Psat5g233240*	Unknown gene	422221988	*Psat5g207040*	511578964	*Psat5g257360*	1172	102.7	7.7	PsCam006936_5161_789 ; PsCam038944_23937_299	TSW04-	TSW - Burstin *et al.*[7] SW, TSW - Gali *et al*.[13]	*#N/A*									
mQTL3.4	chr5LG3	567365719	*Psat5g299720*	2OG-Fe(II) oxygenase superfamily	566380665	*Psat5g299280*	568296029	*Psat5g299920*	17	131.8	0.03	PsCam034779_20141_1638 ; PsCam053662_35502_3991	SN04-, SN06-, SN08-, SN10-, SN11-, SPC06+, SPC010+, SPC11+, SW04-, SW06-, SW08-, SW10-, SW11-, TSW04-, TSW08+	SN, SW, TSW - Burstin *et al.*[7] SW - Gali *et al.*[13] SW, SPC - Klein *et al*.[10]	*Glyma15g01500.1*	*Glyma.15g012100*	*Gm15:964961..966487*			MQTL12,13,14		qProt-15-1 *Gm15_3633885* ; qProt-15-2 *Gm15 31011761*	qPro15-1	
mQTL4.1	chr4LG4	11038786	*Psat4g009760*	Intracellular non-membrane-bounded organelle	1743557	*Psat4g001960*	20433499	*Psat4g014520*	290	6.82	6.3	PsCam056984_37672_88 ; PsCam008438_5952_431	SW08+	SW - Gali *et al.*[13]	*Glyma08g11250.1*	*Glyma.08g106800*	*Gm08:8221536..8221919*	Satt632	Seed weight 36-10, Seed protein 30-4					
mQTL4.2	chr4LG4	42061945	*Psat4g028480*	Xanthine/uracil/vitamin C permease	30049059	*Psat4g021160*	54024236	*Psat4g036960*	364	22.39	4.8	PsCam005173_3921_366 ; PsCam038342_23380_122	SPC04-, SPC11-, TSW06-	TSW - Gali *et al*.[13]	*Glyma13g17960.1*	*Glyma.13g119200*	*Gm13:23194349..23196671*				qPro-13-1 ; qPro-13-2	qProt-13-1 *Gm13_BLOCK_2883 529 3082036*		GqPC13: *Glyma13G123500 (23091289)*
mQTL4.3	chr4LG4	147602594	*Psat4g084600*	2OG-Fe(II) oxygenase superfamily	117446584	*Psat4g071040*	178101781	*Psat4g094840*	546	50.63	7.64	PsCam043089_27118_711 ; PsCam059345_39539_779	SPC04+, SPC06+		*Glyma17g17810.1*	*Glyma.17g163900*	*Gm17:14630769..14636634*	BARC-051665-11191	Seed protein 36-17; Seed yield 31-1, 36-1 Seed protein 26-2					
mQTL4.4	chr4LG4	271800566	*Psat4g139360*	SRP54-type protein + GTPase domain	229359999	*Psat4g123400*	315404846	*Psat4g161720*	876	80.94	8.36	PsCam044314_28211_1843 ; PsCam035946_21100_283	SPC06-, TSW04+, TSW11-		*Glyma17g06400.1*	*Glyma.17g056500*	*Gm17:4284008..4289540*				qPro-17-1	qProt-17-1 *Gm17_BLOCK_6574 500 6577175*		
mQTL4.5	chr4LG4	369227093	*Psat4g181120*	BURP domain	320544838	*Psat4g164800*	417776284	*Psat4g205800*	922	105.4	10.5	PsCam001458_1216_3409 ; PsCam046293_29776_939	SPC11+		*Glyma08g24780.1*	*Glyma.08g230600*	*Gm08:18879210..18881761*	BARC-027788-06671	Seed Ala 1-2; Seed Pro 1-1; Seed Ser 1-1; Seed weight 7-1	MQTL2	qPro-8-1	qProt-8-1 *Gm08_BLOCK_1623 7107_16278243* ; qProt-8-2 *Gm08_BLOCK_4202 6048 42200505*		
mQTL5.1	chr3LG5	40364479	*Psat3g017840*	Unknown gene	13496546	*Psat3g004680*	67064585	*Psat3g031920*	640	18.84	10.29	PsCam037375_22461_2049 ; PsCam044994 28731 4247	TSW04-, TSW06-		*Glyma07g06730.1*	*Glyma.07g061300*	*Gm07:5436276..5440341*							
mQTL5.2	chr3LG5	176232116	*Psat3g085120*	MatE	144455569	*Psat3g068320*	208140583	*Psat3g105520*	855	55.9	9.59	PsCam034798_20158_236 ; PsCam012541_8505_720	SPC06+	SPC - Burstin *et al.*[7] SPC, Gali *et al.*[14]	*Glyma19g29860.1*	*Glyma.19g120200*	*Gm19:37773990..37778474*				qPro-19-1	qProt-19-1 *Gm19_37500961* ; qProt-19-2 *Gm19_BLOCK_3888 6258 38944167*		GqPC19: *Glyma19G236600 (47615818)*
mQTL5.3	chr3LG5	435352873	*Psat3g206400*	Alpha/beta hydrolase fold	434277272	*Psat3g205320*	436339177	*Psat3g207360*	51	112.91	0.37	PsCam009698_6488_1702 ; PsCam004880_3686_1478	SPC06-, TSW04-, TSW06-	TSW, SN, SPC - Burstin *et al.*[7]	*Glyma20g24780.1*	*Glyma.20g113800*	*Gm20:35570468..35571526*	BARC-029461-06196	Seed protein 48-1, 36-26; GWAS Seed set 1 G53.1; Seed weight 37-11; Seed set 5-47	MQTL18	qPro-20-1	qProt-20-1 *Gm20_5531497* ; qProt-20-2 *Gm20_BLOCK_2711 1387_27111623* ; qProt-20-3 *Gm20_BLOCK_3099 5685_31177423* ; qProt-20-4 *Gm20_BLOCK_4328 8485 43465351*	qPro20-1	
mQTL6.1	chr1LG6	33786851	*Psat1g024160*	Ammonium Transporter Family	3521432	*Psat1g002480*	63895910	*Psat1g041160*	884	13.9	10.41	PsCam042987_27022_1291 ; PsCam023473_13286_428	SN11+	SW - Gali *et al*.[13,14]	*Glyma10g26690.1*	*Glyma.10g132300*	*Gm10:35668982..35670484*							
mQTL6.2	chr1LG6	179602535	*Psat1g101120*	AP2 domain	118331306	*Psat1g074560*	240558639	*Psat1g122920*	1079	47.95	7.36	PsCam035272_20467_4972 ; PsCam043936_27889_1794	SPC10-, TSW11+	SW, TSW - Gali *et al.*[13] TSW - Gali *et al.*[12]	*Glyma16g27950.1*	*Glyma.16g164800*	*Gm16:32378037..32380353*	BARC-028369-05854	Seed set 5-34; Seed yield 31-9		qPro-16-1			
mQTL6.3	chr1LG6	339253643	*Psat1g187800*	Glutaredoxin	311313139	*Psat1g162520*	367259508	*Psat1g218360*	1292	86.17	12.84	PsCam050644_33215_1066 ; PsCam037643_22718_391	TSW06-, TSW10-	SW, TSW - Gali *et al.*[13] SW - Gali *et al.*[14] SPC - Tar'an *et al.*[5] SPC - Burstin *et al.*[7]	*Glyma15g10350.1*	*Glyma.15g096700*	*Gm15:7511876..7512184*	BARC-025753-05037	Seed protein 4-5, 4-6; Seed weight 11.2					
mQTL7.1	chr7LG7	60542282	*Psat7g036360*	SBP domain	51210245	*Psat7g031600*	69196947	*Psat7g040040*	199	20.51	2.87	PsCam020840_11620_1382 ; PsCam044103_28028_519	SN08-, SPC08+, SPC10+	SPC - Gali *et al.*[13]	*Glyma05g38180.1*	*Glyma.05g204100*	*Gm05:38768940..38771734*	BARC-020301-04546	Seed protein 9-1, 7-1, 34-1, cqSeed protein-014 ; Seed yeild 20-1, 15-1	MQTL1				GqPC5: *Glyma.05G070600 (5637601)*
mQTL7.2	chr7LG7	206761590	*Psat7g125120*	PLAC8 family	202181919	*Psat7g122680*	211451196	*Psat7g127960*	126	58.52	0.96	PsCam051665_34135_1538 ; PsCam000487_426_1043	SN06-, TSW04+, TSW06+, TSW09+, TSW11+	TSW, SW - Burstin *et al.*[7] SN - Klein *et al*.[10]	*Glyma02g15020.1*	*Glyma.02g133200*	*Gm02:13767171..13769267*							
mQTL7.3	chr7LG7	345409046	*Psat7g184840*	Probable lipid transfer	319631535	*Psat7g166760*	371178765	*Psat7g195480*	668	74.66	8.38	PsCam056507_37317_287 ; PsCam027452_16087_1460	SPC08-, SN11+, SW11+		*Glyma11g12810.1*	*Glyma.11g120400*	*Gm11:9187099..9187413*			MQTL3				GqPC11-1 (24782059) ; GqPC11-2: *Glyma11G234600 (37461551)*
mQTL7.4	chr7LG7	409725211	*Psat7g206160*	PPR repeat	359853588	*Psat7g190640*	459632407	*Psat7g229640*	884	88.38	10.93	PsCam050926_33478_1100 ; PsCam042627 26678 74	TSW04-		*Glyma11g13010.1*	*Glyma.11g122100*	*Gm11:9318009..9319472*							
