# Supplementary material for: Meta-analysis of QTL reveals the genetic control of yield-related traits and seed protein content in pea
Source: Sci Rep. 2020 Sep 28;10:15925. doi: 10.1038/s41598-020-72548-9 (PMC7522997; doi:10.1038/s41598-020-72548-9)
Supplement: Supplementary file 9 — Supplementary Table 6. [file 41598_2020_72548_MOESM9_ESM.pdf]

Meta-analysis of QTL reveals the genetic control of yield-related traits and seed protein content in pea

Anthony Klein[1*], Hervé Houtin[1], Céline Rond-Coissieux[1], Myriam Naudet-Huart[1], Michael Touratier[1], Pascal Marget[2,1] and Judith Burstin[1]

[1] Agroécologie, AgroSup Dijon, INRAE, Univ. Bourgogne, Univ. Bourgogne Franche-Comté, F-21000 Dijon, France

[2] INRAE, UE 0115 DIJ Domaine Expérimental d'Epoisses. Centre de recherche Bourgogne-Franche-Comté, F-21110 Breteniere, France

*** Correspondence:**
anthony.klein@inrae.fr

Table S6 : Passport data and phenotypic information related to the parental lines of mapping population

Species/Subspecies	Accession name	INRAE code	Cultivation status	end of use	Foliage type	Flower color	Internode length	Hilum color	Cotyledon color
Pisum sativum subsp. *sativum*	Ballet	DCG0390	Cultivar	Dry pea	afila	white	short	clear	green
Pisum sativum subsp. *sativum*	Cameor	DCG0251	Cultivar	Garden pea	leaflet	white	short	clear	yellow
Pisum sativum subsp. *sativum*	Cerise	DCG0354	Germplasm	Fodder pea	leaflet	purple	tall	black	yellow
Pisum sativum subsp. *sativum*	China	DCG0033	Landrace	Fodder pea	leaflet	white	tall	black	yellow
Pisum sativum subsp. *sativum*	Kazar	DCG0227	Cultivar	Dry pea	leaflet	white	short	clear	green
Pisum sativum subsp. *sativum*	Melrose	VFD0106	Cultivar	Fodder pea	leaflet	purple	tall	black	yellow
Pisum sativum subsp. *sativum*	Sommette	DCG0249	Cultivar	Garden pea	leaflet	white	short	clear	green
Pisum sativum subsp. *sativum*	VavD265	DCG0265	Landrace	Fodder pea	leaflet	purple	tall	black	yellow
