# Supplementary material for: Meta-analysis of QTL reveals the genetic control of yield-related traits and seed protein content in pea
Source: Sci Rep. 2020 Sep 28;10:15925. doi: 10.1038/s41598-020-72548-9 (PMC7522997; doi:10.1038/s41598-020-72548-9)
Supplement: Supplementary file 10 — Supplementary Legends. [file 41598_2020_72548_MOESM10_ESM.docx]

**Figure S1** : Frequency distribution of Pop3 to Pop11 lines adjusted means observed between 2004 and 2011 at INRAE Dijon in the field trials Domaine d’Epoisses and in glasshouse (*g*). The arrowheads indicate the mean value of parental lines: *Ca* Caméor ; *Vav*, VavD265 ; *Ba*, Ballet ; *Mel*, Melrose ; *Ka*, Kazar ; *Ch*, China ; *So*, Sommette ; *Ce*, Cerise. *SN* seed number per plant, *SW* seed weight per plant (g), TSW thousand seed weight (g) and SPC seed protein content (% of seed dry weight).

**Figure S2** : Pearson correlation coefficients between phenotypic traits observed in Pop3 to Pop11 for each environment from 2004 to 2011 in the filed trails and in glasshouse (*.*) at INRAE Dijon. *SN* seed number per plant, *SW* seed weight per plant (g), *TSW* thousand seed weight (g) and *SPC* seed protein content (% of seed dry weight). *, ** and *** significant correlation at the *P*<0.05, *P*<0.01 and *P*<0.001 probability level, respectively.

**Figure S3** : Phenotypic variability of RIL populations for foliage type, flower color, internode length, hilum color and cotyledon color, and for internode length in the subpopulation tested in the 2011 field trial.

**Table S1** : Mean parental and recombinant inbred values of Pop3 to Pop11, standard deviation, heritabilities and significance of genotype effect measured between 2004 and 2011 at INRAE Dijon. ^a^ phenotyping in the fields at INRAE Dijon, Domaine d’Epoisses, * phenotyping in glasshouse at INRAE Dijon. ^b^ *SN* seed number per plant, *SPC* seed protein content (% of seed dry weight), *SW* seed weight per plant (g), *TSW* thousand seed weight (g). ^c^ heritability [h² = 1-(1/F)] (F : value from Fisher test), ^d^ Significance of genotype effect, *P*-value from ANOVA test. *na*: missing value.

**Table S2** : Genotype, environment and genotype-by-environment interaction effects for the seed number per plant (SN), seed weight per plant (SW), thousand seed weight (TSW) and seed protein content (SPC) measured in field trials at INRAE Dijon.

**Table S3** : QTL parameters detected in multi-population Pop3 to Pop11 from 2004 to 2011. ^a^ QTL position from the first marker of the linkage group (in cM Haldane), ^b^ *SN* seed number per plant, *SPC* seed protein content (% of seed dry weight), *SW* seed weight per plant (g), *TSW* thousand seed weight (g), *04* 2004, *06* 2006, *08* 2008, *09* 2009, *10* 2010, *11* 2011, ^c^ *P* peak value at the QTL position for each variable, ^d^ Phenotypic variance explained by each QTL, ^e^ Position of the lower and upper ends of the QTL confidence intervals from the first marker of the linkage group (in cM Haldane), ^f^ Allelic value of parental lines.

**Table S4** : QTL prediction to metaQTL for seed weight (SW), seed number (SN), thousand seed weight (TSW) and seed protein content (SPC) from Pop3 to Pop11 between 2004 and 2011. For each linkage group, position (in cM Haldane) and confidence interval (CI) of metaQTL are indicated. The QTL prediction membership of metaQTL (mQTL) are calculated from 0 to 1.

**Table S5** : List of genes in pea metaQTL and homologus in soybean. For each pea metaQTL, peak gene and QTL references are identified. Soybean genes were searched from SoyBase^36^ (http://www.soybase.org)

**Table S6** : Passport data and phenotypic information related to the parental lines of mapping population.
